# BiPSim: a flexible and generic stochastic simulator for polymerization processes

**DOI:** 10.1038/s41598-021-92833-5

**Published:** 2021-07-08

**Authors:** Stephan Fischer, Marc Dinh, Vincent Henry, Philippe Robert, Anne Goelzer, Vincent Fromion

**Affiliations:** 1 INRAE, MaIAGE, Université Paris-Saclay, Jouy-en-Josas, France; 2 INRIA Paris, Paris Cedex 12, France

**Keywords:** Stochastic modelling, Software, Cellular noise

## Abstract

Detailed whole-cell modeling requires an integration of heterogeneous cell processes having different modeling formalisms, for which whole-cell simulation could remain tractable. Here, we introduce BiPSim, an open-source stochastic simulator of template-based polymerization processes, such as replication, transcription and translation. BiPSim combines an efficient abstract representation of reactions and a constant-time implementation of the Gillespie’s Stochastic Simulation Algorithm (SSA) with respect to reactions, which makes it highly efficient to simulate large-scale polymerization processes stochastically. Moreover, multi-level descriptions of polymerization processes can be handled simultaneously, allowing the user to tune a trade-off between simulation speed and model granularity. We evaluated the performance of BiPSim by simulating genome-wide gene expression in bacteria for multiple levels of granularity. Finally, since no cell-type specific information is hard-coded in the simulator, models can easily be adapted to other organismal species. We expect that BiPSim should open new perspectives for the genome-wide simulation of stochastic phenomena in biology.

## Introduction

The amount of single cell data available is rapidly increasing, providing an unprecedented access to the heterogeneity of phenotypes within a population of cells^1,2^. This heterogeneity largely stems from the stochastic nature of cellular processes such as transcription and translation and, more importantly, of their interactions^3^. Whole-cell simulations are promising avenues for understanding how complex interactions between cellular processes can shape the mapping between genotype and phenotype in a stochastic context^4,5^. However, the full stochastic simulation of a whole cell remains intractable^6,7^, since it would necessitate the simulation of more than $10^{14}$–$10^{16}$ (random) events per replication cycle^8,9^. Instead, whole-cell simulations rely on hybrid schemes^10–15^, where abundant biological species are handled with an ordinary differential equation scheme, whereas low-abundance species are described by stochastic differential equations. For example, in the whole-cell simulator for the organism *Mycoplasma genitalium*^4^, the cell is decomposed into modules operating together, each module corresponding to a specific cellular processes such as metabolism or protein translation. The bottleneck due to the computational tractability was by-passed by simulating each module differently, e.g., the metabolism by a deterministic model, non-metabolic processes such as the protein translation by a simplified and well-established stochastic model. Having different simulation modules in interaction is an important step towards whole-cell simulation, as it allows to focus modeling efforts on one cell process at a time.

Although the *M. genitalium* simulator is an incontestable milestone in whole-cell modeling, process descriptions were hard-coded, which complicates the adaptation of the simulator to other organisms. An alternative to integrated whole-cell models is to rely on models that follow a standardized representation and can be inter-operated^16^. For example, metabolic processes can be represented as a network of reactions^17^ described by the Systems Biology Markup Language (SBML)^18^. SBML is a flexible format where every compartment, every chemical, every reaction is defined in a format that is both biochemically informative and easy to parse for simulation. SBML models can be simulated by different types of solvers, such as constraint-based models^19–21^ or stochastic simulators such as COPASI^22^, which proposes deterministic solvers based on ordinary differential equations (ODEs) and several implementations of Gillespie’s Stochastic Simulation Algorithm (SSA)^23^ for efficient stochastic simulation. However, a SBML file containing all reactions involved in detailed non-metabolic processes (such as the transcription of all genes nucleotides per nucleotide, the translation of all mRNAs codon per codon as in^4^) would contain several gigabytes of information with at least $4 \times 10^6$ reactions for *Escherichia coli* (more than 4000 genes with average length 1 kb)^24^. Generating, storing and loading such a file in any software is unrealistic.

Such combinatorial complexity typically arises in non-metabolic processes because reactants can adopt multiple states, such as the phosphorylation of protein residues. If each state is modeled as a separate chemical species, biochemical reactions must be duplicated, as each reaction only applies to one state. Rule-based languages^25,26^ such as BNGL^27^, Kappa^28^, or rxncon^29^, circumvent this problem by defining rules that apply to multiple species (including species involved in a complex) or states that match a pre-defined pattern. These languages allow to define complex regulatory events or transduction processes with a minimal set of rules. Another alternative to reduce the number of reactions are meta-languages such as Antimony^30^ and PySB^31^, which define reaction modules that can later be imported or expanded into an SBML or a rule-based model. However, for simulation, rule-based models must be converted into standardized reactions. In network-based approaches, rules are used to exhaustively enumerate possible reactions and reactants^32^. For example, in the Fc$\epsilon$RI signaling model^33^, 24 rules become 58,276 reactions^34^. These reactions can then be stored in a standardized format, such as SBML, or a solver-specific format, such as CPS for COPASI. In network-free approaches^28,35–37^, reactants are represented as individual particles; the matching of rules with particles allows to determine which reactions can be performed dynamically. While network-free approaches avoid the need to exhaustively list reactions and species, they tend to be less efficient than network-based approaches^34^. Particle-based representations are time and memory-intensive, particularly for large systems^35^. The Hybrid Particle/Population (HPP) model^35^, integrated in the BioNetGen framework^36^, offers an interesting trade-off: while reactants are modeled as particles by default, the user can lump related particles (usually having a small number of internal states and affected by small number of rules) into populations, enabling memory and time savings.

While rule-based models provide concise representations for regulatory events, template-based polymerization processes, such as transcription, translation, or DNA replication, currently lack strong representations and simulation standards, with difficulties arising from gene specificity (e.g., reactions involved in transcription are different for every gene), and dependency on spatial information (e.g., what nucleotide to use for the next step of transcription depends on the position of the polymerase)^21,24^. Indeed, the relevant biological annotations, such as the position of promoters or terminators, are provided as global positions along the DNA or RNA and cannot be easily handled by rules, which are based on local motifs. As a result, transcription and translation are never explicitly modeled as polymerization processes, instead only the regulation of transcription and translation is considered^16,38^. The polymerization is represented in a one-step model, which makes it difficult to accurately represent sequence-dependent regulation based on known biological annotations, such as the position of T-box regulation sites^39^. While the detailed description of transcription and translation involves millions of possible states, all polymerization processes share a common pattern: the step-by-step addition of new elements specified by a template sequence defining the polymer in production. Ideally, we would like to represent these processes in a format that provides the composability of SBML-based models, but have more freedom in the type of reactions that we can represent, such that reactions related to the same process (e.g., translation) do not need to be represented more than once (e.g., once per protein)^40^.

In this paper, we introduce BiPSim, a flexible stochastic simulation framework for template-based polymerization processes designed to serve as an intermediate between rule-based models and SBML-based models. Like SBML-based models, cell processes are declared as a set of reactions in input files (limited hard-coded information about the cell), but BiPSim defines new classes of molecules and reactions that handle sequence-based processes. Like rule-based approaches, only a limited number of reactions (one reaction per type of nucleotide or amino acid) is necessary to describe sequence-based processes in generic terms. To ensure maximal scalability, BiPSim uses a hybrid simulation scheme similar to HPP, where freely diffusing chemicals and template sequences are modeled as populations, while chemicals that are bound to a sequence are represented as lightweight particles. For these particles, the set of realizable reactions is computed on the run, depending on the current pool of chemicals and their current location along the template sequence. BiPSim reactions are simple enough to be easily understood (e.g. chemical reaction, binding, translocation) but abstract enough to represent complex processes at various levels of description with a limited number of reactions. All reactions are integrated by a standard Gillespie approach, facilitating extension by adding new reactions or combining multiple sets of reactions. We show how BiPSim can be used to define and simulate processes involved in gene expression (replication/transcription/translation) at various levels of detail. We show that fully stochastic simulations of a cell cycle remain tractable for a detailed version of the gene expression processes, as simulations rely on a constant-time implementation of Gillespie’s SSA^41^ which compares very favorably with more standard implementations of the exact Gillespie’s SSA. Finally, we exemplify how processes can be extended, simulating the injection of a drug targeting the elongation phase of translation and the annotation of a T-box regulation process for the *tyrS* gene.

## Results

### BiPSim description

BiPSim is an open-source command-line tool for the stochastic simulation of sequence-based processes based on the Gillespie algorithm. The software is implemented in C++, runs on Linux/Mac and is licensed under the GNU GPL licence.

### Reactant/reaction types

**Figure 1:**
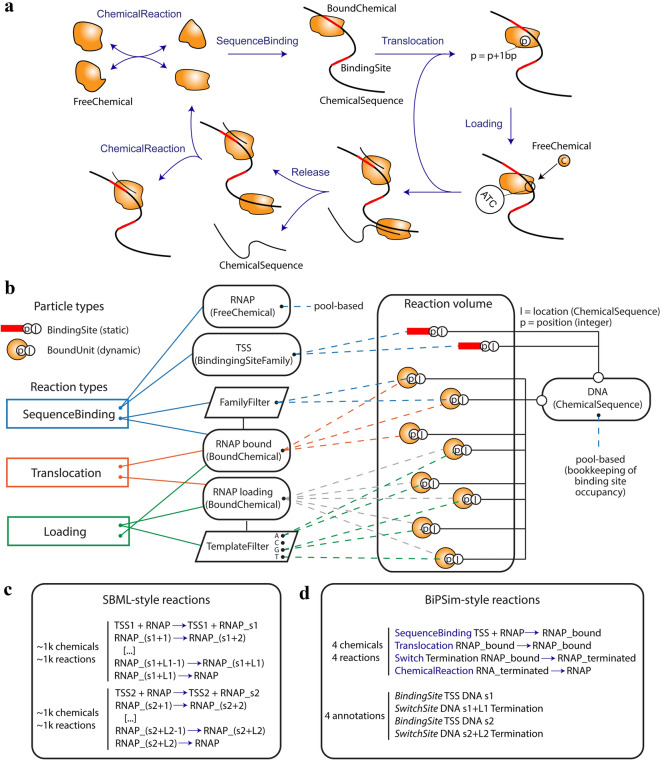
BiPSim defines a set of generic reactions handling polymerization processes. (**a**) BiPSim reactions and reactants were designed to facilitate description of sequence-based processes. (**b**) Illustration of BiPSim’s hybrid simulation scheme for a simplified transcription process involving a binding step, a translocation step and a loading step. The reaction volume contains all the particles that are created dynamically during the simulation (e.g., a $\texttt{BoundUnits}$ is created when a $\texttt{FreeChemical}$ binds to a $\texttt{ChemicalSequence}$ through the $\texttt{SequenceBinding}$ reaction). Colored lines indicate which particle is susceptible to participate in which reactions, and how this information flows from particles to reactions through a bookmarking process involving $\texttt{BoundChemicals}$ and $\texttt{Filters}$. (**c**) Simplified submodel of transcription based on chemical reactions, defining only the translocation and termination subprocesses. In order to record the position of the RNA polymerase, we must create one chemical and one reaction per state. The number of reactions rises very quickly (typically thousands of reactions per RNA), in particular if we want to define these processes for multiple RNAs or we consider adding more detailed steps. (**d**) Same simplified model as c, but with BiPSim reactions. Because BiPSim uses individual-based modelling to represent RNA polymerases ($\texttt{BoundChemicals}$), their position is stored as an attribute of the chemical and does not need to be specified in any of the reactions. The set of reactions is only defined once: when more detailed steps are added, they automatically apply to all RNAs. *TSS* transcription start site, *TTS* transcription termination site, *RNAP* RNA polymerase.

BiPSim relies on a set of computer classes managing both chemical and sequence-based reactions efficiently (Fig. 1). In order to design the classes of BiPSim reactions and reactants, we first reviewed the molecular mechanisms that compose different cell processes. We then introduced a minimal set of reactants (e.g. chemicals, sequences, binding sites) and reactions types suitable to describe most known cell processes, especially sequence-based biochemical reactions (e.g. binding, translocation, loading). Each reaction is associated with a rate proportional to the molecule number of each reactant (see Supplementary File 1).

Briefly, the ChemicalReaction represents a standard (potentially reversible) reaction applied to freely diffusing chemicals (named FreeChemical). SequenceBinding represents the binding of a freely diffusing FreeChemical to a ChemicalSequence (e.g. DNA or RNA) through an unoccupied BindingSite, creating an instance of a BoundChemical (implemented using particle-based modeling). A Translocation moves a specific instance of a BoundChemical along its ChemicalSequence; the movement occurs according to a predefined step (typically 1 nucleotide or 3 nucleotides). A Loading consumes and releases FreeChemicals depending on the sequence that is being read by a specific particle of a BoundChemical (typically loading of a nucleotide or a charged-tRNA). The previous reactions can be combined arbitrarily, enabling binding of multiple instances of BoundChemicals to the same ChemicalSequence, potentially through multiple BindingSites. A Release reaction creates a new ChemicalSequence that depends on the BindingSite and current position of a BoundChemical (e.g. an RNA product corresponding to a specific promoter/terminator combination). A ChemicalReaction can also transform an instance of a BoundChemical into freely diffusing FreeChemicals, implying that the BoundChemical is detached from its ChemicalSequence.

In Fig. 1c,d, we show a toy example where the detailed transcription of two mRNAs is described either using chemical reactions or BiPSim reactions. Using only chemical reactions, we need to define 2000 reactions and 2000 chemicals corresponding to every position of the polymerase along the DNA. In BiPSim, that position is stored in instances of BoundChemicals, so we need only four reactions, describing conditions for binding, translocation and release of the polymerase, and four chemicals, representing the binding and termination sites. If we consider all polymerization processes, switching to the BiPSim formalism results in a drastic reduction of the number of reactions to handle (see Table 1). For instance, a full description of the replication process would necessitate $38 \times 10^6$ chemical reactions while the same process is described by only 11 BiPSim reactions (Table 1).

### Hybrid simulation based on pools and particles

The sequence-based reactions defined by BiPSim are based on lightweight BoundUnit particles (Fig. 1b). Although the use of particles is reminiscent of rule-based simulation, BiPSim is not based on rules, trading genericity for simulation efficiency. Instead, sequence-based reactions and reactants implement a bookmarking system to evaluate reaction propensities dynamically.

Technically, every time a FreeChemical binds to a BindingSite, a particle is created. This particle stores two internal states: its location (the ChemicalSequence it bound to) and its position (along the sequence)(Fig. 1b). When a sequence-related reaction occurs, it may change or access the internal state of a particle. For example, a Translocation reaction changes the position of the particle, while the Loading reaction accesses the position of the particle to know what bases it is currently “reading” along the sequence (Fig. 1a). Sequence-based reactions also change the external state of BoundUnits. For example, after loading a chemical, a polymerase may enter its translocation state. In this case, the corresponding particle is detached from the BoundChemical representing polymerases in loading phase and re-attached to the BoundChemical representing polymerases in translocation phase (Fig. 1d).

Compared to rule-based models, all particles have the same representation (internal states for location and position) and are unable to form complexes. Another key difference is that, in order to compute propensity, there is no pattern matching between particles and rules. Instead, sequence-based reactions and reactants use various bookmarking mechanisms to map particles with the reactions in which they can participate. For example, in the simplified transcription process shown in Fig. 1b, all particles attached to the “RNAP bound” BoundChemical are susceptible to participate in Translocation. SequenceBinding and Loading apply to subsets of particles from a given BoundChemical, e.g., particles that are still on the binding site they originally bound to (thus susceptible to unbind) or particles that are currently reading the “T” base (thus susceptible to load a ATP nucleotide). These subsets are bookmarked using the FamilyFilter and TemplateFilter classes. The simplicity of the bookmarking mechanisms and the particle representation enables extremely efficient simulation of sequence-based reactions. The update of internal states and the re-assignment of particles to BoundChemicals is negligible compared to the propensity update and reaction selection steps, making sequence-based reactions almost as efficient as pool-based reactions involving FreeChemicals. Furthermore, since all particles have the same representation, they can be recycled throughout the simulation, avoiding expensive memory allocation costs.

### Limited biological hard-coded information

Simulated models are completely defined by input files, which declare all molecules and reactions. While the classes of reactions and reactants necessarily constrain the types of processes that can be represented, the simulation system starts empty and all biological processes must be completely defined from scratch. We used a text format for input files where declaration of reactions can be easily interpreted as classical biochemical descriptions (Fig. 1). The reactions can be easily combined, allowing for different levels of descriptions within a single simulation, as we will show in a later example, where 5% of genes follow detailed description of transcription and translation, and the remaining 95% follow aggregated descriptions of the same processes.

### BiPSim structure and simulation algorithm

**Figure 2:**
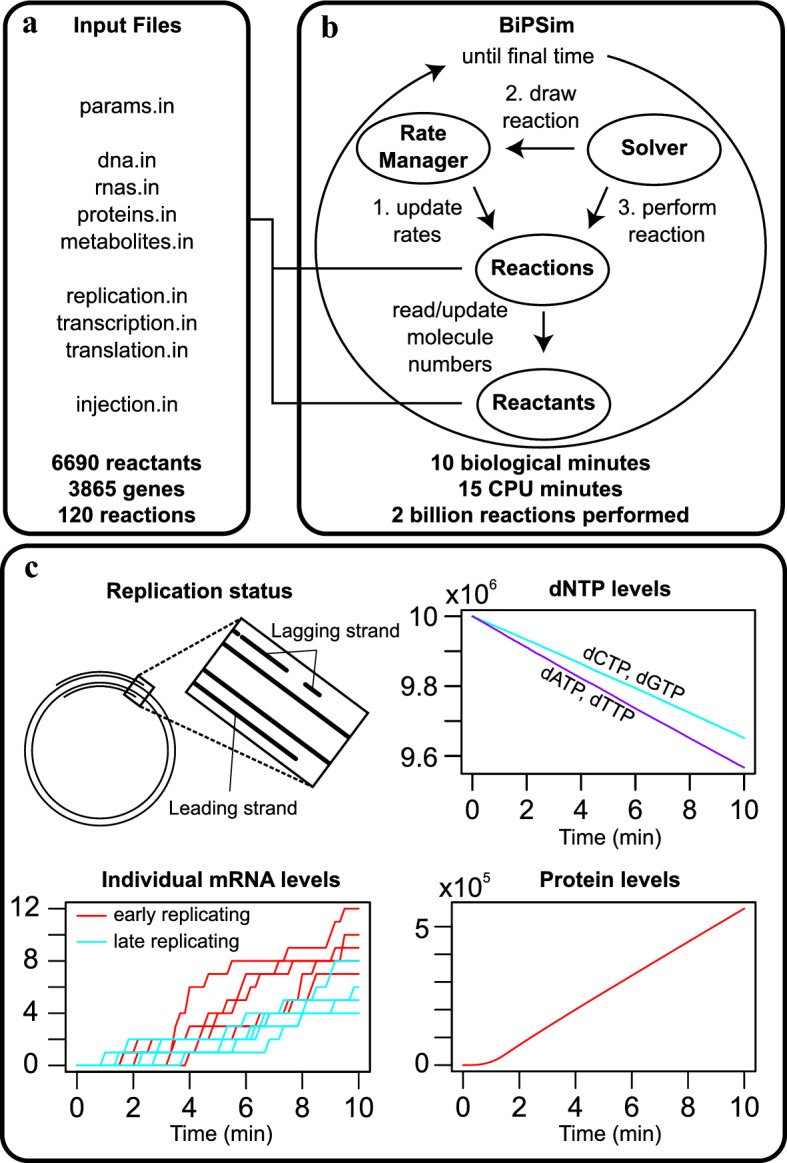
Simulation of genome-wide bacterial gene expression. (**a**) Input files describing detailed models for replication, translation and transcription. (**b**) BiPSim workflow. The simulator instantiates the reactants and reactions declared in the input files. The solver runs an iterative loop in which reaction rates are updated and the next reaction is drawn according to the Gillespie algorithm. (**c**) Snapshot of simulation results obtained after 20% of the simulated cell cycle. Vertical axes display molecule numbers. Top left: replication status and zoom on one of the replication forks. Top right: dNTP consumption for DNA replication reflects the AT bias in chromosome composition of *B. subtilis*. Bottom left: mRNA levels of five genes located near the origin of replication (red) and of five genes that have not been replicated yet (blue). Bottom right: total protein production.

BiPSim contains three main modules: (1) The Input module instantiates reactants and reactions according to input files. (2) In the Simulation module, a Solver controls the following simulation loop (Fig. 2B): (2a) A RateManager updates reaction rates when necessary; (2b) The RateManager selects a reaction with a probability proportional to its rate; (2c) The reaction event is performed: numbers of reactants are updated. (3) The Output module writes a user-defined selection of simulation results to text files.

BiPSim includes three implementations of variants of Gillespie’s exact algorithm^15,42^ (selecting the next reaction to be performed): the direct method^23^ (complexity *O*(*R*), where *R* is the number of reactions [*O*(*f*(*n*)) indicates that the running time is a linear function of *f*(*n*) where *n* is the parameter of interest. *O*(1) means that the running time is independent of the size of the input.)], a binary search method^43^ (complexity *O*(*logR*)), and a composition rejection method^41^ (complexity *O*(1)). By default, it uses the latter algorithm, whose computational effort does not depend on the number of reactions (see Supplementary File 1), allowing for an efficient simulation of large systems. We further validated that BiPSim’s implementations are correct by comparing simulation outcomes with theoretical predictions (see Supplementary File 2).

**Table 1:** Number of reactions necessary to define detailed models of processes using either simple chemical reactions or BiPSim reactions.

Process	Chemical reactions	BiPSim reactions	BiPSim annotations
Replication	38,000,000	11	2
Transcription	8,000,000	10	8000
Translation	10,000,000	38	16,000

For BiPSim reactions, it is necessary to provide annotations about gene products, typically transcription start site and transcription termination site for RNAs, ribosome binding site and stop site for proteins.

### Performance of BipSim

We use simple models to evaluate the performance and consistency of BiPSim. In this first set of simulations, we check that the number of mRNAs and proteins produced are consistent across different levels of aggregation. We assume that initiation rates are the same for all proteins and mRNAs, in order to easily visualize consistency within and across aggregation levels: protein numbers should reflect the number of mRNA variants that carry them.

**Figure 3:**
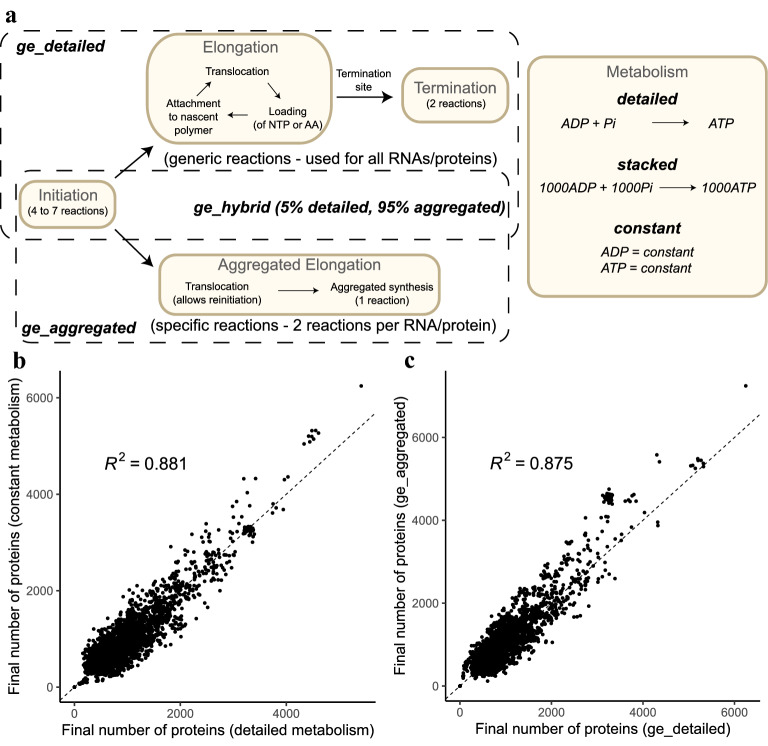
(**a**) Reactions used to represent transcription and translation. The initiation subprocess is the same in all simulations. By default, transcription and translation follow detailed elongation (base per base and codon per codon) and termination. In parallel, aggregated elongation reactions can be used to synthesize RNAs or proteins in 2 reactions. They create a shortcut for a given RNA or protein. (**b**) Final number of proteins for ge_detailed using either constant or detailed metabolism. Each dot here corresponds to a *B. subtilis* gene, e.g. *dnaA* or *sigA*. (**c**) Final number of proteins for ge_detailed and ge_aggregated using constant metabolism.

We used BiPSim to simulate temporal replication and genome-wide gene expression (transcription and translation) in bacteria for three levels of granularity in the process description (Fig. 2, Sup. Fig. 1). We used three levels of description for chain elongation in transcription and translation (Fig. 3a):

- ge_detailed: nucleotide per nucleotide and codon per codon,
- ge_aggregated: by a single aggregated reaction,
- ge_hybrid: by a combination of ge_detailed for 5% of mRNAs and 12% of proteins, and ge_aggregated for the remaining mRNA and protein species.

In all simulations, replication was simulated base per base. Moreover, the steps of initiation and termination for transcription and translation are identical in the three descriptions. Finally, both messengers and proteins are not degraded and accumulate throughout the cell cycle.

We collected gene sequence features for *Bacillus subtilis* (DNA, 2612 transcription units, 3896 proteins, promoters, terminators, ribosome binding sequences, etc.), realistic parameters from literature (see Methods) and generated the input files of BiPSim using scripts (see Fig. 2a). Models ge_detailed, ge_aggregated and ge_hybrid contained 60, 12,300 and 13,034 reactions respectively. The input files containing the full list of reactions are provided with the BiPSim archive.

#### Performance tests

In all our models, we included energetic molecules such as ATP and GTP, for example for the loading of amino acids onto tRNAs, translation initiation or ribosome translocation (Supplementary Figure 1). Theoretically, these metabolites need to be regenerated by the metabolic network, which we did not simulate. Therefore, we kept the concentrations of energetic molecules such as ATP and GTP constant. However, to study the impact of including metabolic reactions in the reaction network, we considered three methods for metabolite renewal, where we simulate the recycling of byproducts such as AMP and GMP (Fig. 3a):

- Detailed: pseudo metabolic reactions are used to regenerate metabolites one by one.
- Stacked: pseudo metabolic reactions are used to regenerate metabolites by stacks of 100 or 1000 molecules.
- Constant: all metabolites have a constant concentration throughout the simulation.

We ran all simulations using the same initial numbers of metabolites and proteins (Supplementary Table 1) for one cell cycle. On a standard computer architecture, BiPSim simulates up to 2,500,000 reaction events per second. The runtime for one cell cycle ranged from 1 min for the most aggregated model (ge_aggregated) to approximately 1 h for the most detailed model (ge_detailed), while the hybrid model (ge_hybrid) took approximately 5 min to complete (see Table 2).

**Table 2:** BiPSim run times for the gene expression models.

Models	Metabolic reactions	Run time (one cycle)	Reactions/s
ge_detailed	Detailed	1 h 4 m 57 s	2,567,503
	Stacked	35 m 20 s	1,849,735
	Constant	30 m 33 s	2,126,666
ge_hybrid	Stacked	5 m 48 s	1,648,895
	Constant	4 m 29 s	1,978,002
ge_aggregated	Stacked	1 m 37 s	1,219,402
	Constant	1 m 6 s	787,128

The models define the granularity of the simulations (aggregated, detailed or hybrid processes). The column “Metabolic reactions” indicates which of the three simulation strategies was used to integrate metabolic reactions (e.g., regeneration of ATP).

A snapshot at 20% of the simulated cell cycle confirmed that DNA replication, dNTP consumption, mRNA and protein production were progressing at the expected rate and reflected known biological properties of the genome, such as the GC ratio (Fig. 2c). In all simulations, around 3 million proteins are produced in one cell cycle, which is consistent with the number of proteins produced during the *E. coli* cell cycle^44^. What is more, there was a strong consistency in the number of proteins produced across genes, independently of the model used for metabolism (Fig. 3b, $R^2 = 0.88$) or the level of aggregation (Fig. 3c, $R^2 = 0.88$). These results suggest that BiPSim successfully manipulates and integrates different levels of description, offering a trade-off between run time and the possibility to describe a subset of processes at a very detailed level. Moreover, the three cases for metabolite renewal also illustrate the capability of BipSim to couple the stochastic simulator and the metabolism, either as a set of chemical reactions within the stochastic simulator, either externally as inputs.

Finally, another facet of BiPSim’s flexibility is illustrated in Supplementary file 2. By varying only the abundance of the active form of the replication initiation factor (DnaA-ATP), BiPSim was capable of simulating multifork bacterial replication^44^.

#### Extension of the gene expression model with tetracycline injection

**Figure 4:**
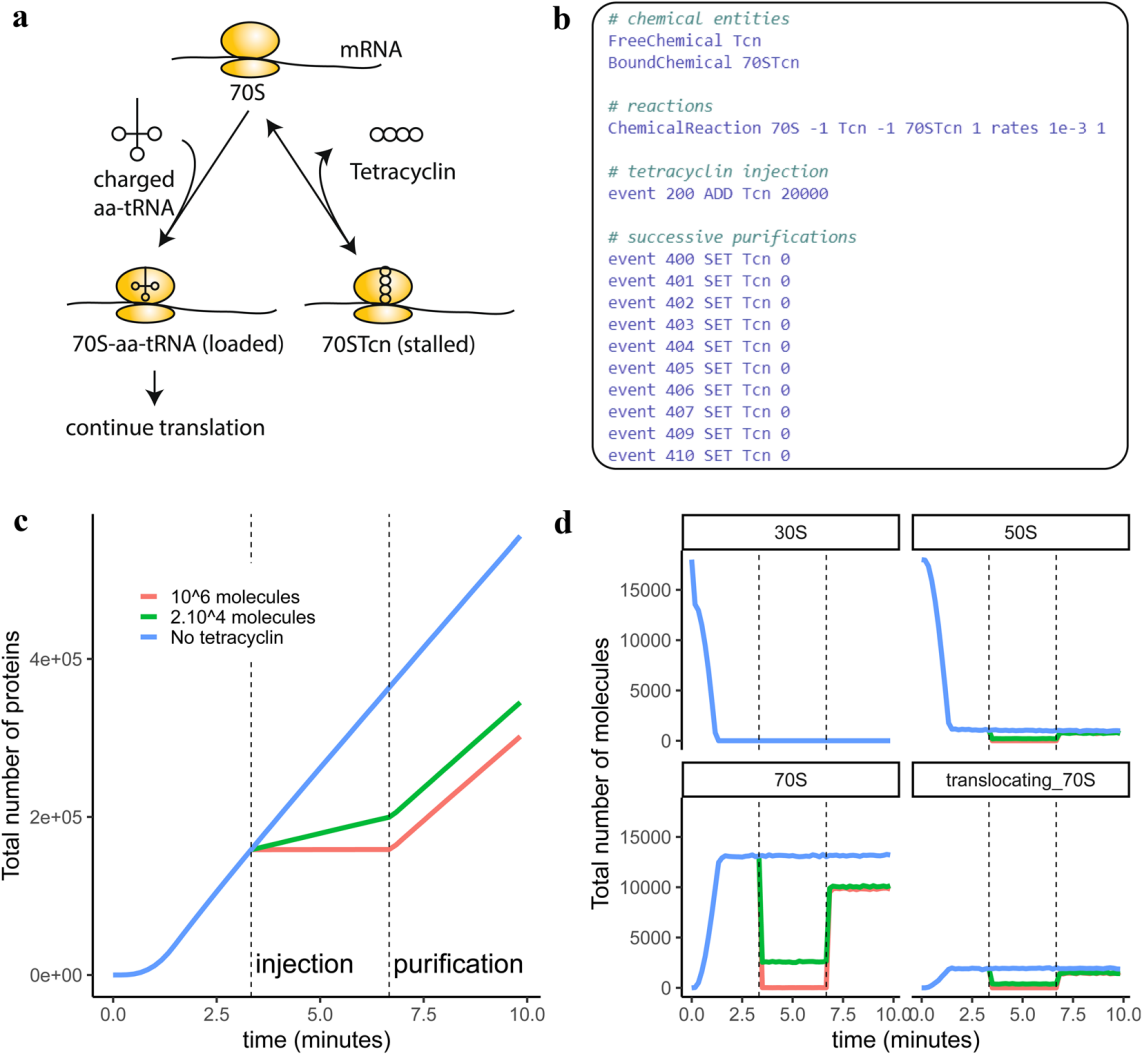
Extension of genome-wide expression model with injection of tetracycline. (**a**) Tetracycline competes with the charged-tRNA recruitment by the ribosome, decreasing translation rates for all proteins. (**b**) The ge_detailed model is extended by adding one file representing tetracycline injection, all other files remaining unchanged. (**c**) Simulated impact of tetracycline injection and purification on the total protein number. (**d**) Simulated impact of tetracycline injection on the different forms of ribosomes: 30S and 50S (freely diffusing), 70S (loading), and translocating_70S (translocation).

One of the key features of using a Gillespie based formalism is that models can be easily extended by adding only a few reactions. To illustrate this point, we simulated injections and purifications of tetracycline by extending the ge_detailed model. Tetracycline is an antibiotics that inhibits translation elongation by competing with charged-tRNAs during 70S ribosome loading (Fig. 4a). In order to simulate the action of tetracycline, we added a new file to the ge_detailed model, which is shown in its entirety in Fig. 4b (all other files are unchanged). The new file defines the new chemicals, the interaction between tetracycline and the 70S ribosome, as well as series of events, which can be used to define injection of tetracycline at $t=200$ s and purification events starting at $t=400$ s.

As expected, the competition of tetracycline with charged-tRNAs for ribosome loading slows down protein production, with higher tetracycline concentrations resulting in higher inhibition of protein production (Fig. 4c). In particular, because of the low dissociation rates of 70S ribosomes from tetracycline, only a limited number of ribosomes are available for loading new charged-tRNAs (Fig. 4d). After several rounds of purifications, some of the tetracycline remained in the cell (only free tetracycline is washed away, the bound form remains), which has a long-lasting effect on the pool of available ribosomes (Fig. 4d).

#### Extension of the gene expression model with T-box regulation

**Figure 5:**
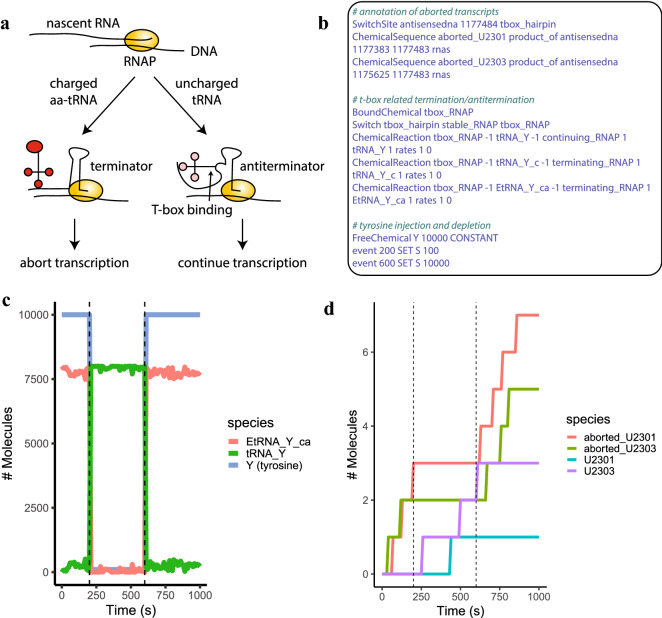
Extension of genome-wide expression model with T-box regulation. (**a**) Schematic illustrating T-box regulation. (**b**) Reactions and chemicals added to the ge_hybrid to implement T-box regulation, all other files remaining unchanged. (**c**) Evolution of the number of tyrosine and tyrosine-related tRNAs over time. Tyrosine shortage is simulated by depleting tyrosine at t $=$ 200 s and re-injecting it at t $=$ 600 s. (**d**) Evolution of the cumulative number of *tyrS*-carrying transcripts over time.

Transcription and translation regulatory mechanisms can be represented using BiPSim’s Switch and SwitchSite. In the gene expression models, transcription termination sites are considered as a Switch: when such sites are reached by the RNA polymerase, it automatically switches from the elongation state to the termination state. The individual sites where these switches occur are gene-dependent and annotated using SwitchSites. To further illustrate how these two entities can be used to annotate and implement regulatory events, we consider the example of T-box regulation on the tyrosyl-tRNA synthetase gene (*tyrS*), where the T-box motif was first identified^45,46^. T-box regulation acts through the binding of tyrosine tRNAs to the leader region of the *tyrS* RNA. This mechanism leads to abortive transcription through an early terminator that can be counter-acted by the formation of an antiterminator in the presence of uncharged tRNAs (Fig. 5a).

To implement T-box regulation, we extended the ge_hybrid model as follows. First, we added the *tyrS* transcription units to the pool of transcription units that need to be simulated step-by-step, which allows to implement the regulatory site through the Switch mechanism. Then, we added a new file to the model containing: (a) the annotation of the regulatory site, (b) the reactions occurring when a chemical reaches the regulatory site, (c) events allowing to simulate tyrosine shortage (Fig. 5b). We annotate the position of the T-box regulatory site as a SwitchSite, along with the corresponding aborted transcripts. We then define that when an RNA polymerase (RNAP) meets any T-box annotated site, it should change its state to “tbox_RNAP”. If the tbox_RNAP binds to an uncharged tyrosine-tRNA, the RNAP resumes transcription; if it binds a charged tyrosine-tRNA, transcription is aborted. As expected, while tyrosine is abundant and tyrosine-tRNAs are in a charged state, transcription of the *tyrS* genes is aborted (Fig. 5c,d). Shortly after the shortage of tyrosine, transcription of *tyrS* proceeds normally. This process is reversed once tyrosine is re-injected.

#### Comparison of performance with other stochastic simulators

In this section, we compare BiPSim’s implementation of the Stochastic Simulation Algorithm (SSA) with implementations from other simulation tools. Because different tools have different applications, we emphasize that we focus on performance in a general setting, i.e., models that can be described in SBML format and can thus be simulated by numerous simulation tools. As there are no sequence-related models involved, all models have been implemented in BiPSim using the pool-based FreeChemicals and ChemicalReactions and none of the particle-based chemical entities or reactions.

**Figure 6:**
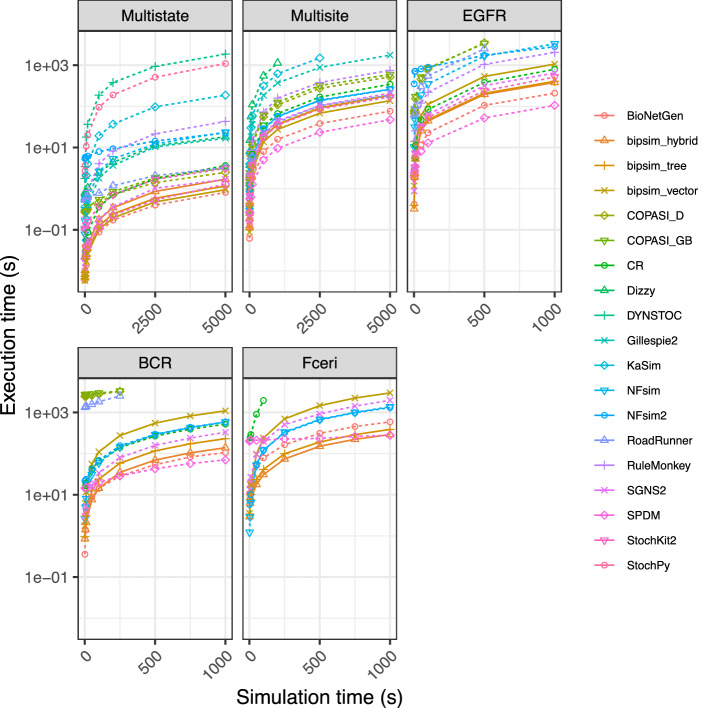
Comparison of BiPSim run times on a benchmark with popular Gillespie-based implementations. The benchmark contains 5 models of increasing combinatorial complexity (Multi-state: 8 reactions, Multi-site: 288 reactions, EGFR: 3749 reactions, BCR: 24,388 reactions, Fc$\epsilon$RI: 58,276 reactions). The performance of the 3 BiPSim implementations are shown as full lines, the performance of other implementations is shown as dashed lines.

First, we computed BiPSim’s performance on a recent benchmark^34^ comparing 16 implementations of the SSA over five simulation models with increasing combinatorial complexity. In the original publications, the authors concluded that, even in datasets with high combinatorial complexity, network-based implementations (exhaustive representation of reactant states and reactions) outperformed network-free implementations (particle-based) for large numbers of molecules. We found that BiPSim performed on par with the best network-based implementations (Fig. 6). The only methods that occasionally outscaled BiPSim were BioNetGen and SPDM, which use the Sorting Direct Method (SDM) implementation. This SSA strategy identifies reactions with high prevalence, which proved to be a favorable strategy in use cases where a small set of reactions is extremely recurrent. As we will see below, the performance of a given implementation strategy depends on the simulated use case, so it is only partially indicative of the strength of the implementation. If we match BiPSim with equivalent implementation strategy, we find that the direct method (“bipsim_vector”) clearly outscaled COPASI’s direct method (“COPASI_D”), while the hybrid method (“bipsim_hybrid”) clearly outscaled SSAlib’s composition-rejection implementation (CR).

Note that all the BiPSim simulations in the benchmark are based on pool-based reactions (ChemicalReaction in BiPSim). Given that network-free implementations proved to be inefficient in the original benchmark, one may naturally worry that performance drops when particle-based reactions are used. However, we found that the number of reactions per second between the gene expression models (780k–2M reactions/s) and this benchmark (200k–3M reactions/s) was similar, suggesting that BiPSim efficiently integrates both pool-based and particle-based reactions. We carefully tailored our particle-based reactions to retain an efficiency that is similar to pool-based reactions. More specifically, we implemented particles that only contain minimal information and can be recycled over time, while particle-based reactions are used to implicitly group similar reactions together (Sup. File 1).

Next, we investigated the asymptotic behavior of the SSA implementations on simple linear pathways (n-cascades) with increasing length, which more closely resemble polymerization-like processes. An n-cascade is a linear pathway characterized by its length (number of chemical reactions) and the number of molecules for the initial chemical of the cascade. For our use-cases (Table 3), every n-cascade simulated needed exactly 1,000,000 reaction events until all chemicals have reached the end of the pathway. For this use case, we compared BiPSim with COPASI^22^, BioNetGen^27,36^ and NFSim^37^, three of the most well established biochemical network simulators.

**Table 3:** Time in seconds needed to simulate n-cascades.

Number of reactions	Number of molecules	BiPSim (direct)	BiPSim (tree)	BiPSim (hybrid)	BioNetGen (SDM)	BioNetGen (NFSim)	COPASI (Gibson and Bruck)
10	100,000	0.1	0.1	0.1	0.1	1.7	0.3
100	10,000	0.2	0.1	0.1	0.3	0.9	0.5
1000	1000	1.2	0.2	0.2	123	2.7	4
10,000	100	11	0.3	0.2	218,000	98	567
50,000	20	55	0.8	0.7	–	1977	5473
100,000	10	110	1.4	1.2	–	8487	–
1,000,000	1	1100	13	12	–	–	–

An n-cascade is a linear pathway characterized by the number of reactions and the number of molecules for the initial chemical of the cascade. Every n-cascade simulated needs exactly 1,000,000 reactions until all chemicals have reached the end of the pathway. A dash indicates no measurement, due to prohibitive run time (BioNetGen) or failure to convert SBML model (COPASI).

We found that BiPSim’s implementations dramatically outscaled COPASI and BioNetGen’s implementations (Table 3). For an n-casacade with 50,000 reactions, BioNetGen’s pool-based implementation never completed, while BioNetGen’s network-based implementation and COPASI’s implementation took approximately 100x longer than BiPSim’s linear-time implementation of the SSA (direct method) and 1000$\times$ longer than BipSim’s optimized implementations (binary tree and composition–rejection methods). Contrary to the previous benchmark, NFSim’s network-free implementation outperformed COPASI and BioNetGen’s network-based implementations, but performed only marginally better than COPASI. Overall, these results suggest that differences in implementation can vary widely in performance for large systems of reactions, and that the exact SSA may be efficiently applied to large systems if it is carefully tailored to the application.

As expected from theoretical perspectives, BiPSim’s implementation of the composition-rejection SSA^41^ is essentially constant time (Table 3), although for a larger number of reactions, the loading time of the system of reactions linearly increases, reaching approximately 10s for the n-cascade with 1,000,000 reactions. In comparison, BiPSim and BioNetGen’s implementation of the direct method^23^ and COPASI’s implementation of the Gibson and Bruck’s next reaction method^47^ behaved linearly, as expected from their theoretical *O*(*n*) complexity. More surprisingly, BiPSim’s implementation of the binary tree method, which has a theoretical $O(\log n)$ complexity, yielded similar results to the composition–rejection method. Note that in other applications presented in this paper, we favored the composition–rejection method over the binary tree, as it was significantly more efficient. BioNetGen’s SDM implementation had a surprising behavior, becoming dramatically slower at a high number of reactions. Our understanding is that, in the case of n-cascades, the most prevalent reactions keep changing over time, which is not properly handled by the Sorting Direct Method implementation.

## Discussion

In this paper, we developed BiPSim, an open-source stochastic simulator of sequence-based cell processes. BiPSim defines novel sequence-based reactions and reactants that allow to define template-based sequence simulation processes such as replication, transcription and translation at a high level detail with a limited number of reactions. BiPSim’s design relies on three principles: (1) maximize the number of sequence-based processes that can be represented, in particular through the use of sequence annotations, (2) minimize the number of new reaction and reactant types to limit the amount of hard-coded information, (3) ensure that all new reactions types can be performed efficiently. BiPSim uses an efficient hybrid simulation scheme based on pools, to represent freely diffusing chemicals, and lightweight particles, to represent sequence-bound chemicals. Combined with a constant-time implementation of the Gillespie’s Stochastic Simulation Algorithm with respect to reactions^41^, BiPSim is able to simulate large-scale sequence-based processes stochastically. As we have shown on the three gene expression models, BiPSim could describe different cell processes with a few types of reactants/reactions only, store the description in few input files of reasonable size (see Supplementary File 1), and finally simulate the process behavior stochastically in an efficient way (1 h runtime on a standard computer for one cell cycle for the most detailed model).

The efficiency of BiPSim, combined with the parsimony of rule-based models, suggests that major non-metabolic processes (transcription, translation, regulation and signaling)^16^ can be represented stochastically. In our benchmarks, we obtained significant speed-ups (up to 10,000$\times$) of runtime for as few as hundreds of reactions. State-of-the-art implementations of the SSA, such as the algorithm used by BiPSim, are instrumental in defining new strategies for the simulation of single cells. They allow for simple and efficient simulations of hybrid strategies, enabling the mix of aggregated processes (resulting in a high number of reactions with long reaction rates) and more detailed processes relying on individual-based modeling (such as the ge_hybrid model shown previously, containing multiple generic reactions with short reaction rates) without further consideration of numerical difficulties, due to the exact nature of the SSA. Furthermore, because rule-based models and BiPSim share a common simulation core (the SSA), we can imagine hybrid strategies where sequence-based processes are simulated using BiPSim reaction classes, while regulatory processes are simulating using rule-based models.

Beyond the performance issues of the simulation algorithms, BiPSim shows that the use of an adequate description of cellular processes (in particular for non-metabolic molecular processes) is central, especially in the context of the development of whole cell simulators. Nevertheless, we expect that new types of reactions or reactants (and thus new classes) may be required. However, since the design of BiPSim is modular, adding new reactions and/or reactants should require a limited number of BiPSim modifications (see Supplementary File 1). Finally, our description of cellular process is not specific to prokaryotic cells, which makes BiPSim suitable to simulate also eukaryotic cell processes.

BiPSim provides concise and efficient descriptions of polymerization processes through the implementation of a hybrid pool/particle modeling scheme. However, because BiPSim is not rule based, it shares the same limitations as network-based approaches, in particular regarding combinatorial regulatory events. While we illustrated the simplicity of the representation of T-box regulation, other processes, such as the combinatorial control of gene expression^48^, may result in duplicated reactions in BiPSim. Note that the problem lies in the concision and flexibility of model definitions, rather than computational efficiency (BiPSim efficiently simulates large reaction systems). BiPSim’s design focused on concise representations of sequence-based processes, which can theoretically be combined with rule-based models in future work. On another note, while BiPSim monitors binding site occupancy to correctly compute binding rates, the current version does not implement interactions between bound chemicals, such as the collisions between RNA polymerases^49^ or ribosomes^50^. Finally, $\texttt{ChemicalSequence}$s are considered immutable, which allows to efficiently represent them as a pool. However, processes changing the nature of the sequence, such as point mutations or the degradation of sequences by internal cutting (for example through endonucleases from the degradosome complex^51,52^), generates a combinatorial complexity that must be represented by duplicating the definition of sequences or subsequences.

We expect that BiPSim should open new perspectives for the genome-wide simulation of stochastic phenomena in sequence-based processes. For instance, the impact of specific genomic features such as pauses in transcription or translation elongation, regulatory sites of mRNAs, can be finely simulated for one gene only as well as genome-wide. BiPSim offers the ability to handle multi-level descriptions of processes simultaneously. A few set of processes can be finely detailed while others are coarsely described. BiPSim thus offers the capability of tuning a compromise between simulation speed and model granularity.

BiPSim models, rule-based models and SBML models can all be simulated using the same core simulator, paving the way to a modular representation of whole cells^16^. They enable the fine representation of a wide variety of cellular processes, including transcription, translation and signaling processes. However, cellular processes such as the metabolism or transcription regulation are better represented using different types of models. The key question then is how to combine Gillespie-based models with models based on another solver strategy, such as ODE models, constraint-based models^19–21^ and boolean models^17^. While hybrid schemes have been proposed^10–12^, the whole-cell modeling field would greatly benefit from a dedicated and standardized solving strategy combining all the popular solvers, and where a wide variety of models can be plugged in. Such a representation would enable exceptional freedom in model choices, as modules would be interchangeable, and in granularity, enabling to efficiently combine coarse-grained representations for intensive processes and fine-grained representations for specific signaling, regulation or sequence-based events.

## Methods

### BiPSim implementation

BiPSim is an open-source command-line tool implemented in C++, designed to run on UNIX-based operating systems (Linux and Mac). Along with traditional chemical reactions, BiPSim defines new types of reactions and reactants that can be simulated using Gillespie’s Stochastic Simulation Algorithm (SSA)^23^. New types of reactions and reactants are implemented as separate C++ classes. Reaction classes inherit from a common interface which can be queried by the solver to determine reaction propensities and executing reactions, enabling seamless integration with traditional implementations of the SSA. Similarly, reactant classes share common properties, such as the possibility to add or remove instances of the reactant. While freely diffusing chemicals are simple pools of molecules, sequence-bound molecules are represented individually as particles.

A typical simulation goes through an initialization phase and a solving phase. During initialization, user-defined reactions and reactants are loaded in the simulation system, along with initial conditions. During solving, the solver loops through SSA steps: (1) update reaction propensities, (2) select a reaction, (3) execute the selected reactions. If the selected reaction only involves freely diffusing chemicals, BiPSim behaves similarly to any network-based simulator: the quantities of the affected molecules are updated. However, if a sequence-bound reactant is involved, a random particle from this reactant is drawn. A reaction may change the internal state (position) of the particle. Alternatively, the particle may become a new type of sequenced-bound reactant. In this case, the particle is detached from its current reactant and re-attached to the new reactant type. Some sequence-based reactions only apply to a subset of particles from a given reactant, which are monitored through the use of filters (Fig. 1d). The solver loop is repeated until the end time of the simulation is reached.

Further details about the implementation and interaction of BiPSim classes are provided in Supplementary Files 1 and 2.

### Gene expression models

We generated three gene expression models for *Bacillus subtilis* containing replication, transcription and translation processes. We reviewed the literature to define step-wise models for each process, along with relevant reactants and cofactors (Supplementary Figure 1). We generated the first model (ge_detailed) by representing the polymerization processes nucleotide-by-nucleotide and codon-by-codon. This model contains two major parts. The first major part (files replication.in, transcription.in, translation.in) contains sequence-based reactions that describe how the polymerases and ribosomes move along their template, which chemical they need to load depending on the motif they are reading (nucleotide or codon), and how the polymerization stops when a stopping site is encountered. This description is generic and applies to any *Bacillus subtilis* gene. The second major part (files dna.in, rnas.in, proteins.in) includes all gene-specific sequence annotations, such as promoters, transcription start and termination sites (one set of annotations per gene).

The two other models are simplifications of the ge_detailed model, obtained by aggregating transcription and translation sequence-based reactions as a single reaction for all genes (ge_aggregated) or a subset of genes (ge_hybrid, 95% of transcription units chosen at random). More specifically, the initial binding process of the polymerase is still sequence-based. The particle is then translocation to an arbitrary position within the gene (+50 bases compared to the binding site) in order to make the binding site available to other polymerases. The gene product is then generated in one step and the polymerase is released from its template sequence. As a result, there are two additional reactions per gene represented using the aggregated process. These reactions have been generated using a custom Python script and added to transcription.in and translation.in. All sequence annotations and other reactions in the system remained identical.

### Gene expression models: sequence information

Sequence information for *Bacillus subtilis* was taken from^53^: DNA sequences, position of promoters, transcription starting sites, terminators, ribosome binding sequences (RBS), start and stop codons. A Transcription Unit (TU) can contain several genes, rRNAs or tRNAs. Conversely, a gene can belong to several TUs. The total number of mRNAs in the cell is given by the sum of TUs carrying genes. Models ge_detailed, ge_aggregated and ge_hybrid use native TUs.

### Gene expression models: parameters

#### Models ge_detailed, ge_aggregated and ge_hybrid

Parameters were set to match known values from *B. subtilis* measurements^54^ when available. Otherwise, we used *Escherichia coli* values for a doubling time close to chromosome replication time, that is $\tau \simeq 40$–50 min^44^.

#### Replication

Global replication rate is known to be around 750 bp/s. For simplicity, we set translocation as the limiting step of replication, other steps were set to occur approximately 10–100 times faster. Translocation rate was set to 750 s$^{-1}$, polymerization (attachment of nucleotide) was set to 10, 000 s$^{-1}$ and loading rates were set to 1000–10,000 s$^{-1}$ (exact rates depend on dNTP abundance).

For the lagging strand, the length of Okazaki fragments is known to be around 1000 bp^55,56^. This means that, on average, the replication fork recruits a new DNA polymerase every 1000 bp. Because replication advances at 750 bps/s, a new polymerase is recruited every 1.33 s. The number of DNA polymerases and the recruitment rate were set according to the following mathematical relation

$$\begin{aligned} |DNAP_{free}|r_{recruitment} = 3/4 \end{aligned}$$

We set the number of DNA polymerases to 10 copies per cell^57,58^. We estimate $DNAP_{free}$ to be around 5 or 6 (deducing DNAPs already engaged in polymerization). As a result, we set the recruitment rate to 0.1$s^{-1}$.

#### Initiation and termination of transcription and translation

Initiation and termination were arbitrarily set to last around 1 s. Promoters (resp. ribosome binding sites) all have the same strength (recruitment rate of RNA polymerases resp. ribosomes). Note that initiation time is negligible compared to elongation time. These rates do not affect the total protein production rate.

#### Transcription elongation

Transcription rate is known to be around 50 nucleotides/s. For simplicity, we set translocation as the limiting step of transcription, other steps were set to occur approximately 10–100 times faster. Translocation rate was set to 50 s$^{-1}$, polymerization (attachment of nucleotide) was set to 500 s$^{-1}$ and loading rates were set to 600–2100 s$^{-1}$ (exact rates depend on NTP abundance).

#### Translation elongation

Translation rate is known to be around 20 residues/s. Loading was set as the limiting step of translation, other steps were set to occur approximately 10 times faster. Loading rates were set to approximately 20 s$^{-1}$ (depending on abundance of the different tRNAs), transpeptidation was set to 150 s$^{-1}$ and translocation was set to 150 s$^{-1}$.

### Gene expression models: initial conditions

Inputs of the simulator are given in number of molecules. For simplicity, the volume of the cell is set to be constant at a value of $1 \upmu {\mathrm{m}}^3 = 10^{-15}$L. The conversion from concentration in mole per liter to number of molecules is given by:

$$\begin{aligned} 1 {\mathrm{mM/L}} \simeq 10^{-3}.6.10^{23}.10^{-15} = 6.10^5{ \text { molecules}} \end{aligned}$$

Metabolite pools were set according to the literature (Supplementary Table 1). For simplicity dNTPs were provided in excess ($10^8$ molecules for each dNTP). Amino acids were also provided in excess. NTPs were kept constant to compensate consumption by transcription.

### Simulation of tetracycline injection

The tetracycline model was obtained by extending the ge_detailed model. We added a file describing how tetracycline interacts with the ribosome, as well as tetracycline injection and purification (Fig. 4b). We simulated the injection of 20,000 (injection1.in) and 1,000,000 (injection2.in) molecules of tetracycline. All other files of the ge_detailed model remained unchanged.

### Simulation of T-box regulation

The T-box regulation model was obtained by extending the ge_hybrid model. First, we removed the aggregate reactions describing the production of *tyrS* transcription units (U2301 and U2303), ensuring that their production is simulated nucleotide-by-nucleotide. We then added two files annotating aborted gene products and the T-box terminator site (tbox_regulation.in), the interaction between the transcribing RNA polymerase and tyrosine tRNAs (tbox_regulation.in), and the depletion and re-injection of tyrosine (tyrosine_depletion.in). All other files of the ge_hybrid model remained unchanged.

### Gupta and Mendes benchmark simulations

We started by downloading the benchmark results and SBML models provided by the authors in the original publication’s Supplementary File 1. We then converted the SBML models to BiPSim using custom Python scripts, converting reactants from the SBML models into FreeChemicals and reactions into ChemicalReactions (pool-based reactants and reactions representing freely diffusing chemicals). We followed the original publication’s methodology to generate all use cases (5 independent runs for different combinations of simulation times and initial number of molecules). We ran all BiPSim models and determined simulation times using Python’s time library. We used the results from the original study for the 16 popular simulation tools (available in Supplementary File 1 from the original publication). In the original benchmark, simulations were run in Mac OSX using a 2.9 GHz Intel core i7 processor with 16 GB of RAM. BiPSim simulations were run on a single-threaded CentOS 7 server using a 3.2GHz Intel(R) Xeon(R) Gold 6134 CPU processor with 188GB of RAM (BiPSim’s RAM usage never exceeded 16 GB).

### N-cascade benchmark simulations

We started by generating n-cascades in BiPSim format, SBML format and BNGL format using custom Python scripts. An n-cascade contains $n+1$ chemicals $A_{0}$ to $A_{n}$, with *n* reactions $A_{i} \rightarrow A_{i+1}$. The cascade starts with 1, 000, 000/*n* molecules of chemical $A_0$, such that there are exactly 1,000,000 reactions before all molecules are converted to chemical $A_n$.

The SBML model was converted to a COPASI model using the command line CopasiSE version 4.27. Due to memory issues in CopasiSE during this conversion step, we limited *n* to 50,000. We implemented a script that modified the COPASI model to implement a “Task” of type “timeCourse” with a “Method” of type “DirectMethod” (which corresponds to Gibson and Bruck’s next reaction method, as observed from a model generated by CopasiUI) and set the “Random Seed” parameter to 1. For BioNetGen’s sorted direct method, we ran the BNGL model in BioNetGen 2.5.0 with the function “simulate” with the “method” parameter set to “ssa”. Due to prohibitive run time, we limited *n* to 10,000. For NFSim, we ran the BNGL model in BioNetGen 2.5.0 with the function “simulate” with the “method” parameter set to “nf”. Due to prohibitive run time, we limited *n* to 100,000.

We measured simulation time using Python’s time library for COPASI and BiPSim. Note that simulation time did not include conversion time (conversion from SBML to COPASI model). For BioNetGen and NFSim, we extracted the number following “CPU TIME: simulate” from the standard output as the simulation time (to avoid including conversion time).

### Computer characteristics

All simulations were run on a server with an Intel(R) Xeon(R) Gold 6134 CPU @ 3.20 GHz processor and 188 GB of RAM. BiPSim was compiled under CentOS 7 using gcc 4.8.5 and BOOST 1.71. All simulations were run on a single thread and used less than 4 GB of RAM and could be reproduced on a standard laptop with similar performance (Intel Core Duo 3.00 GHz, 4 GB RAM, Ubuntu 16.04, gcc 5.4.0, BOOST 1.58).

## Supplementary Information

Supplementary Information 1.

Supplementary Information 2.

Supplementary Information 3.

## Data Availability

BiPSim software and documentation can be downloaded at https://github.com/SysBioInra/bipsim.
