# Supplementary material for: BiPSim: a flexible and generic stochastic simulator for polymerization processes
Source: Sci Rep. 2021 Jul 8;11:14112. doi: 10.1038/s41598-021-92833-5 (PMC8266833; doi:10.1038/s41598-021-92833-5)
Supplement: Supplementary file 1 — Supplementary Information 1. [file 41598_2021_92833_MOESM1_ESM.pdf]

BiPSim: a flexible and generic stochastic simulator for polymerization processes - Supplementary Figures and Tables

Stephan Fischer[1], Marc Dinh[1], Vincent Henry[1], Philippe Robert[2], Anne Goelzer[1], and Vincent Fromion[1,*]

[1]Université Paris-Saclay, INRAE, MaIAGE, Jouy-en-Josas, France
[2]INRIA Paris, Paris Cedex 12, France
[*]vincent.fromion@inrae.fr

Molecules	Base value	Number	Source
ATP	$7mM$	$4.2.10^6$	Personal communication
GTP	$5mM$	3.10^6	
CTP	$0.2mM$	$1.2.10^5$	
UTP	$0.5mM$	3.10^5	
active RNAP		1000	[1]
active ribosomes		18000	
tRNA		240000	
EF-Tu	5.4/ribosome	118000	
IF1	0.25/ribosome	5500	
IF2	0.3/ribosome	6600	
IF3	0.2/ribosome	4400	

Table 1: **Supplementary Table 1.** Initial conditions for metabolite and protein numbers.

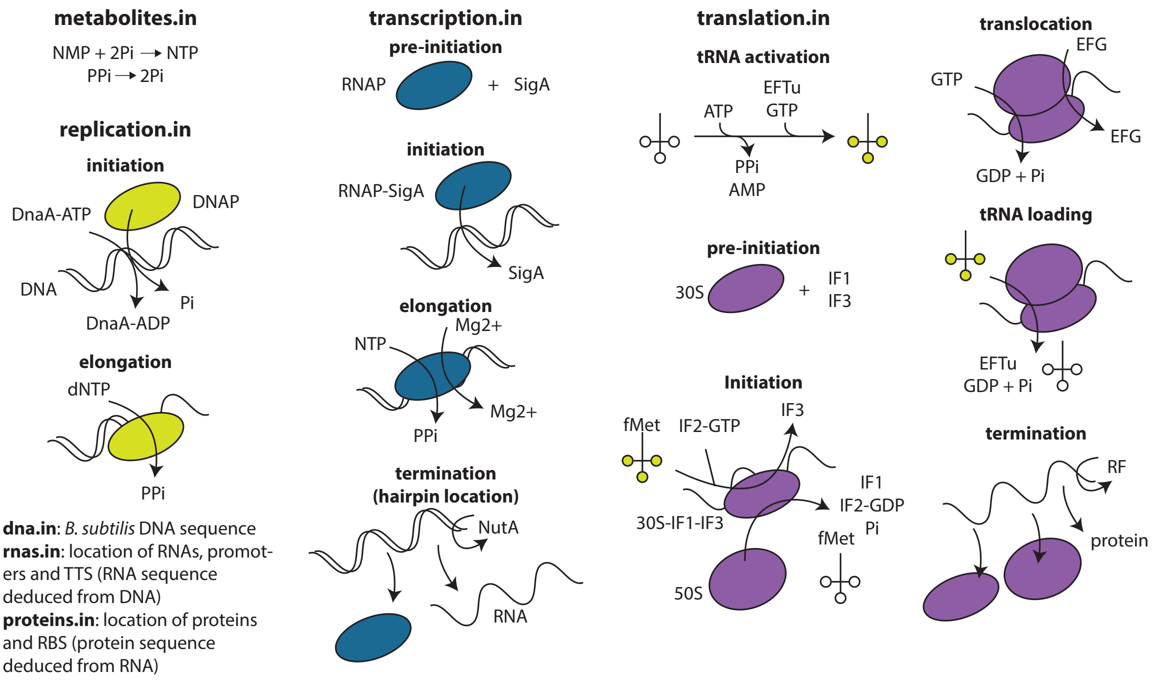

Figure 1: **Supplementary Figure 1.** Schematic of reactions used for the gene expression models (by input file). For simplicity, we only represented the main reactions and sometimes fused several reactions into a single cartoon.

References

[1] Patrick P. Dennis and Hans Bremer, *Modulation of Chemical Composition and Other Parameters of the Cell at Different Exponential Growth Rates*, EcoSal Plus **3** (2008), no. 1 (en).
