# Supplementary material for: BiPSim: a flexible and generic stochastic simulator for polymerization processes
Source: Sci Rep. 2021 Jul 8;11:14112. doi: 10.1038/s41598-021-92833-5 (PMC8266833; doi:10.1038/s41598-021-92833-5)
Supplement: Supplementary file 2 — Supplementary Information 2. [file 41598_2021_92833_MOESM2_ESM.pdf]

BiPSim: a flexible and generic stochastic simulator for polymerization processes - Supplementary Information (Supplementary File 1)

Stephan Fischer[1], Marc Dinh[1], Vincent Henry[1], Philippe Robert[2], Anne Goelzer[1], and Vincent Fromion[1,*]

[1]Université Paris-Saclay, INRAE, MaIAGE, Jouy-en-Josas, France
[2]INRIA Paris, Paris Cedex 12, France
[*]vincent.fromion@inrae.fr

Contents

1 Introduction **4**

2 BiPSim chemical species and reactions in a simple step-by-step example **4**

2.1 Basic chemical species and reactions: pool-based `FreeChemical` and `ChemicalReaction` 4
2.2 Polymer species: single-stranded and double-stranded biological sequences 5
2.3 Binding dynamics along a sequence: particle-based `BoundChemical`, `BindingSite`, and `SequenceBinding` . . . 7
2.4 Polymerization reactions: `ProductLoading` and `Translocation` . . . 9
2.5 Regulation of polymerization: `Switch`, `SwitchSite` and `Release` . . . 9
2.6 Expanding a model with sequence annotations . . . 12

3 Efficient integration of pool-based and particle-based reactions **13**

4 Implementation of reactions and reactants **17**

4.1 Global presentation of the components of the simulator . . . 17
4.2 Reactant hierarchy . . . 18
4.2.1 Reactant . . . 18
4.2.2 Chemical . . . 18
4.2.3 FreeChemical . . . 19
4.2.4 BoundChemical . . . 19
4.2.5 ChemicalSequence . . . 19
4.2.6 DoubleStrand . . . 21
4.2.7 BindingSiteFamily . . . 21
4.3 Reaction hierarchy . . . 22
4.3.1 Reaction . . . 23
4.3.2 ChemicalReaction . . . 23
4.3.3 SequenceBinding . . . 24
4.3.4 Translocation . . . 25
4.3.5 Loading . . . 26
4.3.6 DoubleStrandRecruitment . . . 27
4.3.7 Release . . . 28
4.3.8 Degradation . . . 30
4.3.9 Switches . . . 31
4.3.10 Solver loop . . . 31
4.3.11 Events . . . 32
4.3.12 Input/Output handling . . . 32
4.4 Detailed design . . . 33
4.4.1 Reactants . . . 33
4.4.2 Reactions . . . 37
4.4.3 Solver loop . . . 39
4.5 Formats and Conventions . . . 40
4.5.1 Input format description . . . 40

4.5.2 UML 41

5 Implementation of Gillespie's Stochatic Simulation Algorithm 41

5.1 SSA variants implemented in BiPSim 41

5.1.1 Direct method 41

5.1.2 Binary tree 42

5.1.3 Hybrid method 44

5.1.4 Summary 48

1 Introduction

In the main paper, we briefly present the chemical entities and reactions implemented by BiPSim. This document walks through the choices in design that we made while developing BiPSim (the code is documented using Doxygen, technical details are therefore best found in the Doxygen-generated manual). It highlights the central classes in BiPSim's architecture and how classes interact.

In Section 2, we explain each entity's role by progressively incorporating entities in a simple example. We show how BiPSim's formalism can be applied to multiple genes by first defining generic reactions, then including gene-specific sequence annotations (no further reactions needed). Section 3 provides a brief explanation of BiPSim's integration scheme, specifically how Gillespie's Stochastic Simulation Algorithm was expanded to include both pool-based and particle-based reactions, and how it differs from rule-based models. Section 4 provides a formal description of the base components of all reactants and reactions in BiPSim. Section 5 illustrates the three versions of Gillespie's Stochastic Simulation Algorithm (SSA) implemented in BiPsim.

2 BiPSim chemical species and reactions in a simple step-by-step example

2.1 Basic chemical species and reactions: pool-based `FreeChemical` and `ChemicalReaction`

`FreeChemical`s are the basic chemical species found in any simulator. They are hypothesized to be freely diffusing and interchangeable, thus forming a pool of molecules. When a reaction occurs, it does not matter which instance of the pool is affected, it only matters how many elements are affected. `ChemicalReactions` describe how `FreeChemical`s can be assembled and transformed and correspond to traditional SBML-type reactions.

We illustrate these two classes using a simple example inspired from the transcription process. In bacteria, the RNA polymerase (RNAP) binds to specific promoters by associating to a sigma factor. Both molecules are freely diffusing at this point, which is also the case of the RNAP-sigma complex (also called RNAP holoenzyme). To simulate this process in BiPSim, we first define the 3 chemical entities, as well as the number of initial molecules (Fig. 1). In this example, we will assume that there are 10 RNAPs and 20 sigma factors at the start of the simulation. Then, we define the reaction describing the RNAP-sigma complex formation. To define a reaction, we specify the chemical species involved along with their stoichiometry (negative for reactants, positive for products). Finally, after the `rates` keyword, we specify the rates of formation and dissociation of the complex (Fig. 1).

The second element needed to run BiPSim is a parameter file (Fig. 1). In this file, we specify the random seed, the initial and final time of simulation (0-10s here), the input files defining chemical entities and reactions (a single file at this stage), the output

```
<input/1_free_chemicals.in>
    # chemical entities
    FreeChemical RNAP 10
    FreeChemical sigma_factor 20
    FreeChemical RNAP_sigma

    # reactions
    ChemicalReaction RNAP -1 sigma_factor -1 RNAP_sigma 1 rates 1 1
```

```
<1_params_free_chemicals.in>
    SEED 0
    INITIAL_TIME 0
    FINAL_TIME 10
    INPUT_FILES input/1_free_chemicals.in
    OUTPUT_DIR output/free_chemicals
    OUTPUT_STEP 0.1
    OUTPUT_ENTITIES RNAP sigma_factor RNAP_sigma
    DRAWING_ALGORITHM hybrid
    HYBRID_BASE_RATE 1e-12
```

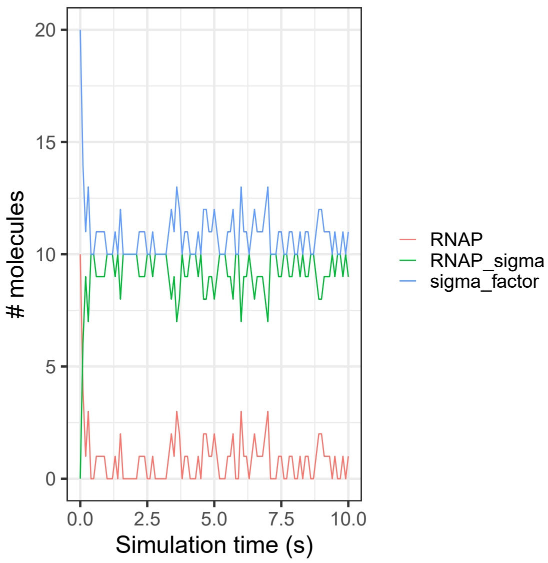

Figure 1: Step 1, complexation of the RNA polymerase (RNAP) and sigma factors. The top left panel shows the BiPSim input file, the bottom left panel shows the BiPSim parameter file, and the right panel shows the simulation output. The two reactants are represented as freely diffusing molecules comparable to species in SBML models.

directory, the output step (at which interval should molecule numbers be recorded?), the chemical entities to record, and the algorithm used for simulation.

At this stage, we are all set to simulate our example by running `bipsim params1_rnap_sigma.in`. BiPSim generates 3 output files: the time course of the molecules (`chemicals.out`), a snapshot of the top 10 reactions that occurred at each time step (`reactions.out`), and a copy of the parameter file used to generate the simulation (`params.out`). Here, we visualize the content of `chemicals.out`. We see that the RNAP-sigma complex forms very rapidly, leading to a steady-state where all RNAPs are in complex form (up to stochastic variability).

2.2 Polymer species: single-stranded and double-stranded biological sequences

The fundamental distinction in BiPSim is between freely diffusing molecules and bound molecules. But before we can explain this distinction, we need something to bind to, i.e. chemical sequences. There are two types of sequences in BiPSim: `DoubleStrandSequences` and `ChemicalSequences`. `DoubleStrandSequences` are modeled after DNA, the idea being that there is a complementary strand that can be deduced from the template strand. In contrast, `ChemicalSequences` only have a single strand, such as RNAs or proteins. Each type of sequence can be represented using a sequence of letters, which can later be used to create a mapping between related sequences (e.g., synthesis of RNA from DNA).

<input/2_sequences.in>

```
# Definition of linear pseudo-DNA
TransformationTable dna2dna A T, C G, G C, T A
DoubleStrandSequence chromosome sense_dna TAGAAAAAGC-
CGAAAAAAATCATAGGTGTCTCCAATAGCTGTTGTAAAAGTGTAATGGTTG-
GGGACACCTGTTCCAAAAAAGTTGTCCAACAGGCGAAACACCTATTCTAAC
ACTGTTGGTAACGTTCGAGAGCAAATAAAACCATAATATAAACACAAAATTG
AGAACTAATGATTAGGATGGAAAGGAGAAATAGGTGTTTCACACCTATTCAA
CACCTAACTAAAGTGTGTCGAACACATCTTCCAACAGGTGTT antisense_d-
na dna2dna LINEAR 1

# Definition of two pseudo-RNAs
TransformationTable dna2rna A U, C G, G C, T A
ProductTable rnas dna2rna
ChemicalSequence rna_1 product_of sense_dna 10 100 rnas
ChemicalSequence rna_2 product_of sense_dna 50 150 rnas 2
```

<2_params_sequences.in> (changes highlighted)

```
INPUT_FILES input/1_free_chemicals.in input/2_sequences.in
OUTPUT_DIR output/sequences
OUTPUT_ENTITIES RNAP sigma_factor RNAP_sigma rna_1 rna_2
sense_dna antisense_dna
# all other parameters identical
```

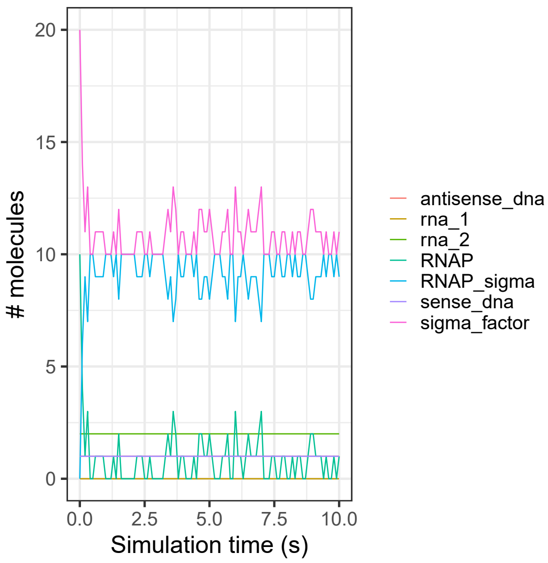

Figure 2: Step 2, definition of chemical sequences (DNA and RNAs). The top left panel shows the additional BiPSim input file (all files shown in previous figures are also part of the model), the bottom left panel shows changes in the BiPSim parameter file, and the right panel shows the simulation output. This example illustrates the two types of BiPSim sequences (double stranded and single stranded) and the two ways to define a sequence (from sequence or as the product of another sequence, using a conversion table).

In our transcription example, we will define a strand of DNA and two potential RNA products. DNA is double-stranded, we thus define it as a `DoubleStrandSequence`. To define it, we provide a name for the whole entity ("chromosome"), a name for the template strand ("sense_dna"), the sequence of this strand, a name for the complementary strand ("antisense_dna"), the identifier of a table which enables to deduce the complementary strand from the template strand ("dna2dna"), the topology of the sequence ("CIRCULAR" or "LINEAR"), and the number of initial molecules. Note the definition of a `TransformationTable`, which can be used to build the sequence for the complementary strand (e.g., "A" becomes "T")(Fig. 2).

Chemical sequences can be either defined by providing a sequence (as we just did for our chromosome), or as a product of another template sequence. For example, we will define two RNA molecules, using our chromosome as a template. First, we define a "dna2rna" `TransformationTable` which maps how the template sequence can be used to generate the RNA sequence (e.g., "A" becomes "U"). We then encapsulate this table in a `ProductTable`, which will later be used to list all the valid products for a given template molecule. Now, we define our two RNAs (Fig. 2), starting with a name ("rna_1"). Instead of writing the sequence of the RNA, we define it as a product ("product_of" keyword) and specify the template ("sense_dna"), the initial and final positions along the template. Finally we provide the `ProductTable` which is used to

compute the sequence of the RNA and list the RNAs as valid products of the sense DNA. Optionally, we can provide the initial number of molecules: here we start with no rna_1 molecule and 2 rna_2 molecules.

We can simulate our new system by extending the previously created parameter file. We add the newly created file as an input and add the new chemicals to the output. Without surprise, the time course of RNAP and sigma are unchanged, as our sequence chemicals are not participating in any reaction yet.

2.3 Binding dynamics along a sequence: particle-based `BoundChemical`, `BindingSite`, and `SequenceBinding`

Chemical sequences create a 1-D space along which molecules can bind. When this happens, molecules stop diffusing and stop being interchangeable, as they will bind to sequences at different locations. This is exactly what is captured by the `BoundChemical` class, which uses a particle-based formalism (`BoundUnits`) where each instance has two internal states: the `ChemicalSequence` it bound to and its current position along the `ChemicalSequence`. In order to initiate the binding, we need to specify what molecules can bind, and where they can bind: this is the role of the `BindingSite`.

We extend our transcription example by enabling the RNAP-sigma complex to bind (Fig. 3). First, we define one binding site (a promoter) for each of our RNA products. To define a binding site, we provide the type of the binding site ("promoter", used later in `SequenceBinding`), the location of the binding site ("sense_dna"), the initial and final position of the binding site (5-15), binding site specific binding and dissociation affinities (1, 0.1), and the "reading frame" of the binding site (10). The last parameter, the "reading frame", is essential to define valid products during a polymerization process, as we will see later. What matters here is that the reading frame should match the initial position of one of the products that we defined. For example, "rna_1" starts at position 10 of "sense_dna", so we define the reading frame for the first binding site as 10.

Once binding sites are defined, we create a reaction to specify which entities bind to the binding site (Fig. 3). This is done using the `SequenceBinding` reaction, starting with a `FreeChemical` ("RNAP_sigma"), the resulting `BoundChemical` ("bound_RNAP") and the type of binding sites ("promoter"). Note that a single `SequenceBinding` reaction will automatically apply to all binding sites of the same type. For example, if we wanted to add other RNA products, we would only need to annotate their binding sites, but we would not need to define any new entity or reaction (see Section 2.6).

We can simulate our current model by extending our parameter file and running BiPSim. Note how the number of bound RNAP never exceeds 2 (Fig. 3). In a `SequenceBinding` reaction, the propensity depends on the number of *available* binding sites. Since there is only one molecule of sense_dna and there are two binding sites, at most 2 RNAPs can bind at the same time. Internally, BiPSim maintains an "occupancy map" of each position along a chemical sequence (`SequenceOccupation`). As `ChemicalSequence`s are treated as a pool, BiPSim does not know to which instance a particular `BoundChemical` is bound, but it knows the aggregate occupancy and can deduce how many binding sites

<input/3_binding.in>

```
# annotation of binding site for each RNA
BindingSite promoter sense_dna 5 15 1 0.1 10
BindingSite promoter sense_dna 45 55 1 0.1 50

# binding reaction
BoundChemical bound_RNAP
SequenceBinding RNAP_sigma bound_RNAP promoter
```

<3_params_binding.in> (changes highlighted)

```
INPUT_FILES [...] input/3_binding.in
OUTPUT_DIR output/binding
OUTPUT_ENTITIES bound_RNAP RNAP_sigma
# all other parameters identical
```

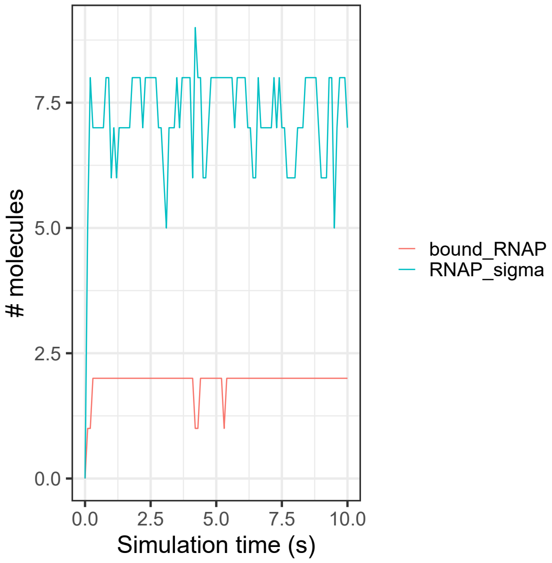

Figure 3: Step 3, definition of binding process. The top left panel shows the additional BiPSim input file (all files shown in previous figures are also part of the model), the bottom left panel shows changes in the BiPSim parameter file, and the right panel shows the simulation output. This example illustrates the annotation of binding sites on one of the template sequences, along with the binding process that allows freely diffusing molecules to become bound molecules (represented as particles). Since binding site occupancy is monitored in BiPSim, at most one molecule can bind at a time on each binding site.

are available for binding.

2.4 Polymerization reactions: `ProductLoading` and `Translocation`

In our current model, RNAPs can bind to the DNA, but they don't move. In this section we will explore how they can be moved along the sequence and used to perform template-dependent reactions.

First, we use the `ProductLoading` class to define template-dependent reactions. To define a `ProductLoading` reaction, we specify an enzyme responsible for the loading ("bound_RNAP") and a `LoadingTable`, which defines which chemical is loaded by the enzyme (Fig. 4). The `LoadingTable` definition starts with a name ("NTP_loading"), then a list of quadruplets defining a template sequence ("A"), the corresponding loaded chemical ("UTP"), the complex that is created by loading the chemical ("translocating_RNAP"), and the rate at which the loading occurs ("0.005"). In this example, the molecule that is consumed is indeed sequence-dependent, but the resulting complex is always the same ("translocating_RNAP"). The reason is that the ATP, CTP, GTP or UTP are polymerized into the nascent RNA, thus we don't consider them to be part of the resulting complex. What becomes of the nascent strand will be explained in the next section.

Next, we move the RNAP along the template sequence using the `Translocation` reaction (Fig. 4). The reaction specifies a moving `BoundChemical` ("translocating_RNAP"), the resulting `BoundChemical` if the translocation succeeds ("bound_RNAP"), the resulting `BoundChemical` if the translocation fails ("stalled_RNAP", e.g. if the end of the DNA is reached), the step size along the template (1 base at a time), and the translocation rate (50).

We simulate the new model by adjusting the parameter file and running BiPSim. As expected, we can see that RNAPs switch between the "bound" and "translocating" states as they move along the DNA template (Fig. 4). Note that the total number of bound RNAP progressively increases. In the previous simulation, only 2 RNAPs could bind simultaneously, as only 2 binding sites were available. Now that RNAPs are able to move along the DNA, binding sites become unoccupied eventually, enabling new RNAPs to bind. Consequently, the consumption of ATP, CTP, GTP and UTP progressively accelerates, as there are more and more nascent RNA strands being synthesized.

2.5 Regulation of polymerization: `Switch`, `SwitchSite` and `Release`

BiPSim allows to define sequence-related events with the implementation of `Switch`es. Contrary to reactions, switches occur instantaneously. In our example, we use a `Switch` to define transcription termination (Fig. 5). When a bound_RNAP reaches any site annotated as "hairpin", it instantly becomes a hairpin_RNAP. This change of state allows to exit the 2-step polymerization process we defined before (loading and translocation). Next, we define the location of the "hairpin" `SwitchSite`s. We add one hairpin site for each RNA, specifying the `ChemicalSequence` it is located on ("sense_dna"), its position ("101") and its type ("hairpin").

<input/4_polymerization.in>

```
# NTPs needed for polymerization
FreeChemical ATP 1000
FreeChemical CTP 1000
FreeChemical GTP 1000
FreeChemical UTP 1000
# possible RNAP states
BoundChemical stalled_RNAP
BoundChemical translocating_RNAP

# loading of NTPs into RNAP (depends on template sequence)
ProductLoading bound_RNAP NTP_loading
LoadingTable NTP_loading A UTP translocating_RNAP 0.005, C GTP
translocating_RNAP 0.005, G CTP translocating_RNAP 0.005, T ATP
translocating_RNAP 0.005

# translocation of RNAP (one bp at a time)
Translocation translocating_RNAP bound_RNAP stalled_RNAP 1 50
```

<4_params_polymerization.in> (changes highlighted)

```
INPUT_FILES [...] input/4_polymerization.in
OUTPUT_DIR output/polymerization
OUTPUT_ENTITIES ATP CTP GTP UTP bound_RNAP RNAP_sigma
translocating_RNAP
# all other parameters identical
```

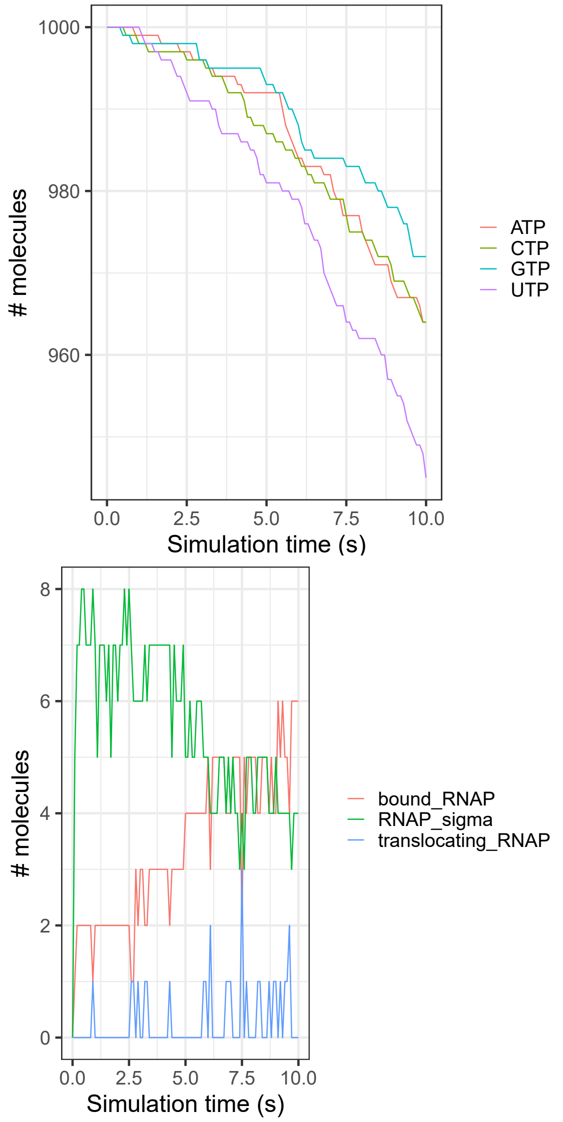

Figure 4: Step 4, definition of the polymerization process through translocation and loading. The top left panel shows the additional BiPSim input file (all files shown in previous figures are also part of the model), the bottom left panel shows changes in the BiPSim parameter file, and the right panels show the simulation output. This example illustrates a transcription-like process, where bound particles move forward one base at a time and load NTPs corresponding to the template they are currently reading (e.g., UTP when reading A). Several polymerases are able to bind to the same template as once: as soon as a binding site is available (because the polymerase translocated away from it), new polymerases are able to bind (as reflected by the increasing number of bound RNAPs).

<input/5_regulation.in>

```
# definition of termination process
BoundChemical hairpin_RNAP
Switch hairpin bound_RNAP hairpin_RNAP

# annotation of termination sites (+1 compared to last transcribed base)
#  - rna_1 goes from 10 to 100 -> hairpin at 101
#  - rna_2 goes from 50 to 150 -> hairpin at 151
SwitchSite sense_dna 101 hairpin
SwitchSite sense_dna 151 hairpin

# if the thermination site leads to a valid product, the product is
released
# and the RNAP enters termination, otherwise continue polymerization
BoundChemical terminating_RNAP
BoundChemical continuing_RNAP
Release hairpin_RNAP terminating_RNAP continuing_RNAP rnas 100
ChemicalReaction terminating_RNAP -1 RNAP_sigma 1 rates 100 0
ProductLoading continuing_RNAP NTP_loading
```

<5_params_regulation.in> (changes highlighted)

```
INPUT_FILES [...] input/5_regulation.in
OUTPUT_DIR output/regulation
OUTPUT_ENTITIES rna_1 rna_2 RNA_sigma terminating_RNAP
# all other parameters identical
```

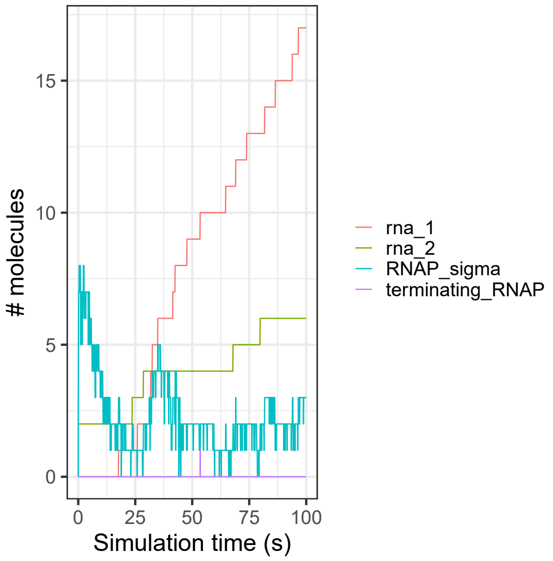

Figure 5: Step 5, definition of regulation sites through switches. The top left panel shows the additional BiPSim input file (all files shown in previous figures are also part of the model), the bottom left panel shows changes in the BiPSim parameter file, and the right panel shows the simulation output. This example illustrates the termination process, where nascent RNAs are released and polymerases are ejected from the template (become freely diffusing again and losing their particle status). The number of produced RNAs increases roughly linearly, as RNAPs that are ejected at the terminator are able to re-bind at the promoter. Since rna_1 and rna_2 overlap, RNAPs transcribing rna_1 temporarily block rna_2's binding site, resulting in lower transcription rates for rna_2.

Finally, we implement the behavior of the RNAP once it reaches a hairpin (Fig. 5). In theory, we could define any reaction, but here we are interested in releasing the nascent strand. This is done using the `Release` reaction, which will automatically determine which product has been generated. To define the `Release` reaction, we specify the input `BoundChemical` responsible for polymerization ("hairpin_RNAP"), the resulting `BoundChemical` if the release was successful ("terminating_RNAP"), the resulting `BoundChemical` if the release was unsuccessful ("continuing_RNAP"), a `ProductTable` used to determine which product has been polymerized ("rnas"), and finally a reaction rate (100). To determine whether the `Release` is successful, BiPSim queries the binding site and termination site of the bound_RNAP (e.g., 50-151), then tries to find a matching product in the `ProductTable` (e.g., rna_1). If a matching product is found, this product is generated, otherwise the RNAP enters a stalled state. We need an additional reaction to define what occurs in this stalled state. In our example, we resume the polymerization process with a `ProductLoading` reaction. Warning: if we had used bound_RNAP instead of continuing_RNAP, we would have created an endless loop! Indeed the switch condition would be met (bound_RNAP on a hairpin site), causing the RNAP to become hairpin_RNAP, then failing to release, becoming a bound_RNAP, switching to hairpin_RNAP, etc.

At this stage, we update the parameter file to include the new reactions and run BiPSim again. As expected, after a short latency period where the first RNAPs bind and start synthesizing, the number of RNAs goes up over time (Fig. 5). Of particular note, the number of copies of rna_1 goes up faster than rna_2. This may be a surprise since the binding rates are identical for the two promoters. However, remember that a binding site can be blocked by any bound chemical, this includes RNAPs in loading or translocation state. Since rna_1 and rna_2 overlap, RNAPs that transcribe rna_1 transiently block the promoter of rna_2, drastically reducing the ability of RNAPs to bind to it.

2.6 Expanding a model with sequence annotations

We defined rna_1 and rna_2 at the very beginning as the product of sense_dna, but they did not appear directly in any of the reactions that followed. They are used to define valid products of our transcription process, similar to simple sequence annotations. Now that the transcription process is fully defined, we can easily extend the set of potential products by providing new annotations, without changing any of the reactions. For each new annotation, we will need to provide 3 elements: the description of the new product, its binding site and its termination site.

As an example, we introduce two new RNAs: an alternative transcription start site (TSS) for rna_1 ("rna_1b") and another RNA on the anti-sense DNA ("rna_3")((Fig. 6)). Note that for rna_1b, the promoter overlaps with the promoter of rna_1, creating a competition between the two promoters. Also note that we do not need to specify the location of the hairpin as it is the same as rna_1. Regarding rna_3, we simply created a copy of rna_1 on the anti-sense strand. Since it is located on a different strand than the 3 remaining RNAs, there is no competition involved and it should be transcribed with

```
<input/6_annotations.in>
  # new RNA overlapping with rna1
  # note: hairpin is identical, does not need to be defined again
  ChemicalSequence rna_1b product_of sense_dna 15 100 rnas
  BindingSite promoter sense_dna 10 20 1 0.1 15

  # new RNA on antisense DNA
  ChemicalSequence rna_3 product_of antisense_dna 10 100 rnas
  BindingSite promoter antisense_dna 5 15 1 0.1 10
  SwitchSite antisense_dna 101 hairpin
```

```
<6_params_annotations.in> (changes highlighted)
  INPUT_FILES [...] input/6_annotations.in
  OUTPUT_DIR output/annotations
  OUTPUT_ENTITIES rna_1 rna_1b rna_2 rna_3
  # all other parameters identical
```

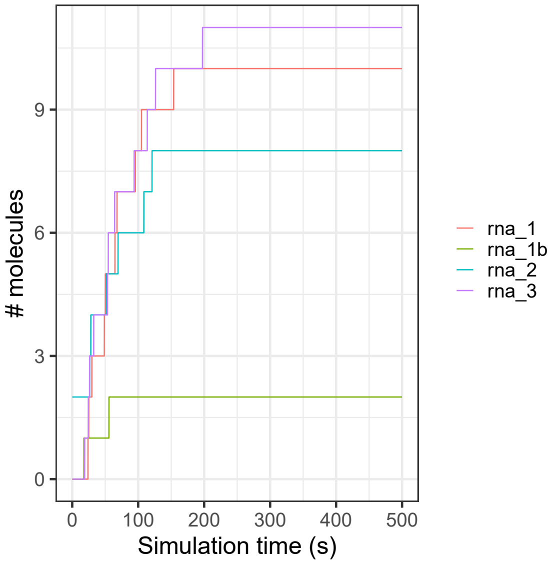

Figure 6: Step 6, model extension through sequence annotations. The top left panel shows the additional BiPSim input file (all files shown in previous figures are also part of the model), the bottom left panel shows changes in the BiPSim parameter file, and the right panel shows the simulation output. This example illustrates how models can be expanded without adding any new reaction. The new file defines annotations for two new RNAs, including promoters and terminators. The binding, polymerization and termination processes have been fully defined in the previous step and automatically apply to the newly annotated RNAs.

high efficiency.

We add our annotation file to the existing model in a new parameter file and run BiPSim. In agreement with our expectations, rna_3 and rna_1 are transcribed at very high levels (Fig. 6). In comparison, rna_1b and rna_2 suffer from the influx of RNAPs binding at the rna_1 promoter.

3 Efficient integration of pool-based and particle-based reactions

BiPSim's efficiency relies on the use of pool-based reactions wherever possible, and the grouping of similar reactions whenever particles (bound elements) are involved. In BiPSim, particles are called `BoundUnits`. Every time a `SequenceBinding` occur, a `BoundUnit` is created and stored under the right `BoundChemical` class (Fig. 7). Every time a reaction occurs, the `BoundUnit` simply transitions to another `BoundChemical` class, changing its "external" state. Internally, all `BoundUnits` stores the same information: their original binding site and their current location. All the complexity and

optimization reside in the reaction classes, which were programmed to aggregate and update the state of `BoundUnits` as efficiently as possible, enabling sequence-based reactions to be almost as fast as pool-based reactions.

The particle-based framework is reminiscent to rule-based modeling languages, such as BNGL (Faeder et al. (2009)) or Kappa (Danos et al. (2007)), in particular the associated simulators NFSim (Sneddon et al. (2011)) and KaSim. Compared to traditional SBML-based models, rule-based models allow to define internal states for each reactant. Figure 7a shows a schematic example of a rule-based model with two proteins A and R. Molecule A has two internal states: a tyrosine Y, which can be either unphosphorylated (U) or phosphorylated (P), and a site r, which can bind to the R molecules. Molecule R has only one internal state: a site A, which can bind to A molecules. The strength of rule-based models is that these states can be modeled independently. For example, the "A-R binding" rule specifies that A and R can bind through the a and r sites, independently of the phosphorylation state of the tyrosine. In an SBML-type model (including BiPSim), we would need to define two reactions: one for the phosphorylated A protein, one for the unphosphorylated A protein. The number of duplicated reaction increases exponentially with the number of internal states. Rules also applies to molecules that are part of a complex. For example, the phosphorylation reaction applies to all A molecules, independently of whether they are bound to R molecules, again avoiding the duplication of reactions.

While rule-based languages allow to define systems with combinatorial complexity using a small number of rules, the number of reactions considered during simulation depends on the simulation strategy. Indeed, rule-based models can be simulated using either a network-based or a network-free approach. In network-based approaches (such as BioNetGen's SSA solver (Faeder et al. (2009))), rules are expanded into SBML-style reactions, effectively duplicating reactions and reactants to list all combinations of internal and complexation states. In contrast, network-free approaches reason that it is simpler to represent individual reactants as particles. This strategy enables to consider only the states that are present at any simulation timepoint, instead of exhaustively enumerating all possible states. As a result, it is not necessary to duplicate rules, but to maintain a mapping between particles and the rules in which they are susceptible to participate. For example, the Kappa language uses a graph-based formalism to map rules and particles (Fig. 7). Each input of a rule and each particle is represented as a graph. A particle can undergo a given rule if there is a subgraph that matches one of the inputs of the rule. Based on this mapping, the simulator can compute the rule propensities, select a random rule, select a random particle eligible for this rule, then change the particle's state according to the rule. The mapping from rules to particles is then updated, allowing to re-compute propensities and select a new reaction to perform.

BiPSim's framework is based on particles, but is not based on rules (no pattern matching between reactions and particles). Instead of the matching process that connects rules and particles, BiPSim connects reactions and particles through new reactant types (Fig. 7). Rule-based models do not contain reaction types, instead they allow to define different particle types with great flexibility (varying number of internal states). In contrast, BiPSim has one particle type (two internal states), but defines several types of

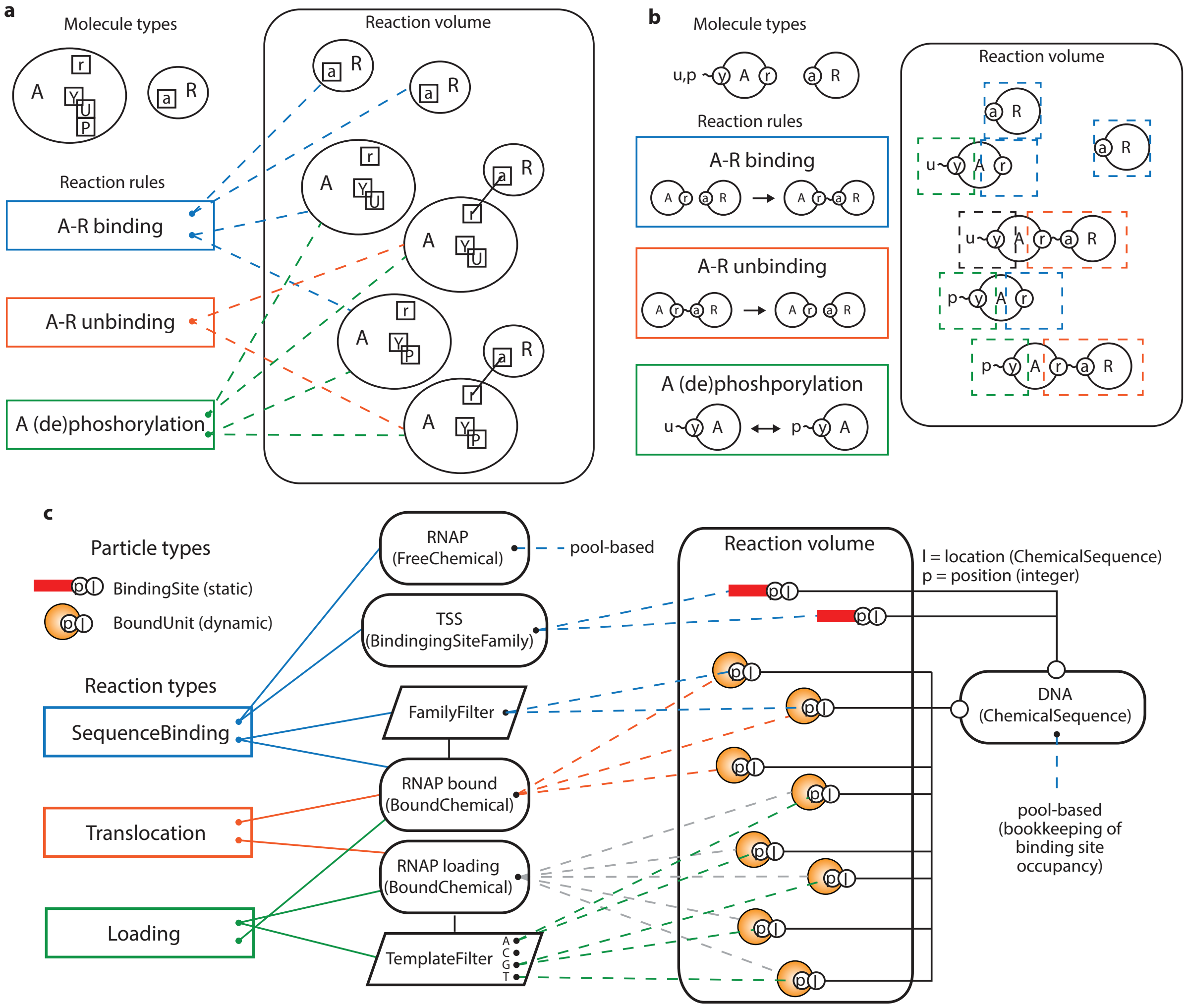

Figure 7: **a** Schematic representation of a rule-based models containing two molecules. Molecule A has two internal states (binding site and tyrosine phosphorylation), molecule R has one internal state (binding site). The model contains three rules: binding of A and R (independently from A's phosphorylation state), unbinding of A and R, and a reversible phosphorylation reaction (independently from). During simulation, molecules are dynamically mapped to the rules that are susceptible to apply to them (highlighted by dotted lines). **b** Schematic representation of Kappa's graph-based formalism to map particles with rules. The model is identical to a, but particles are represented in Kappa style to emphasize their graph structure. A particle is mapped to a rule if a subgraph (highlighted by dotted lines) matches one of the inputs of the rule. **c** Schematic representation of BiPSim's hybrid simulation scheme on a simplistic polymerization example. In contrast to the rule-based formalism, BiPSim defines new types of reactants used to efficiently map particles with reactions (no pattern matching involved). Each reaction is connected with a subset of reactants and filters (full lines), which are used to bookmark and organize particles (dotted lines).

reactants, which are used to contain and organize the particles. BiPSim's bookmarking system trades efficiency (bookmarking is simpler than graph matching) for genericity (BiPSim models do not contain rules and are susceptible to combinatorial explosion).

The `Translocation` reaction provides the most straightforward example of bookkeeping, as the position of individual particles has no influence on the translocation rate. For example, in the schematic transcription process shown in Figure 7, all particles of type "RNAP bound" are susceptible to undergo translocation. The "RNAP bound" maintains a list of all particles attached to it, which can be queried by the translocation reaction at any time point to compute the reaction propensity. As in rule-based models, if the translocation is selected by the Stochastic Simulation Algorithm (SSA), there is a second random selection where one of the candidate particles will be selected. The translocation is then applied to the particle, updating its position (internal change) and its `BoundChemical` type (external change, "RNAP loading" in the example shown).

For the `Loading` reaction, the reaction and reaction propensities depend on the template sequence read by a given particle. As a result, it is necessary to know which template motif particles are reading in order to compute the overall reaction propensity. We found that the easiest solution to this problem is to use filters. Every time a `BoundChemical` is defined as the input of a `Loading` reaction, a `TemplateFilter` is attached to the `BoundChemical`. For example, in Figure 7, "RNAP loading" has an associated `TemplateFilter` that reorganizes its particles depending on the template they are reading (A, C, G, or T). The usage of filters is simple: every time a particle is attached to "RNAP loading", its position is queried and used to deduce its template, then the particle is assigned to the correct slot in the `TemplateFilter`. To compute propensities, the loading reaction uses the TemplateFilter to compute the overall propensity per template, which are then summed to obtain the total propensity. When a loading reaction is performed, it first selects a random template (according to template propensity), then a random particle associated with that template.

For the `SequenceBinding` reactions, individual binding sites act as a static type of particles. All binding sites are created during the initiation of the simulation (according to the user's specification) and remain immutable during the simulation. Each binding site belongs to a family of binding sites, for example "TSS" in the transcription example (Fig. 7). The `BindingSiteFamily` is used to compute the overall propensity across all binding sites (depending on binding site availability, as discussed previously) and to select a random binding site when the reaction is performed (according to binding affinity). The unbinding process uses a `FamilyFilter` that is conceptually similar to `TemplateFilter`, except it is used to monitor particles that are still on their binding site. In the transcription example, there are 3 "RNAP bound" particles. Among these 3 particles, 2 are still on their original binding site, and thus susceptible to unbind. The remaining particle has previously translocated away from its binding site, and thus not susceptible to unbind.

There are additional improvements to ensure efficient handling of particles. We have found that the most costly operations was the handling of memory (creation and destruction of particles). To avoid memory operations, we store all unused `BoundUnits` (when a chemical detaches from a sequence) and recycle them, which greatly improved

BiPSim's scalability. Other important areas of improvement included fine management of housekeeping operations, such as updating reaction propensities only when strictly necessary or careful algorithms to retrieve a given `BoundUnit` from a `BoundChemical` class and pass it to the next one.

4 Implementation of reactions and reactants

4.1 Global presentation of the components of the simulator

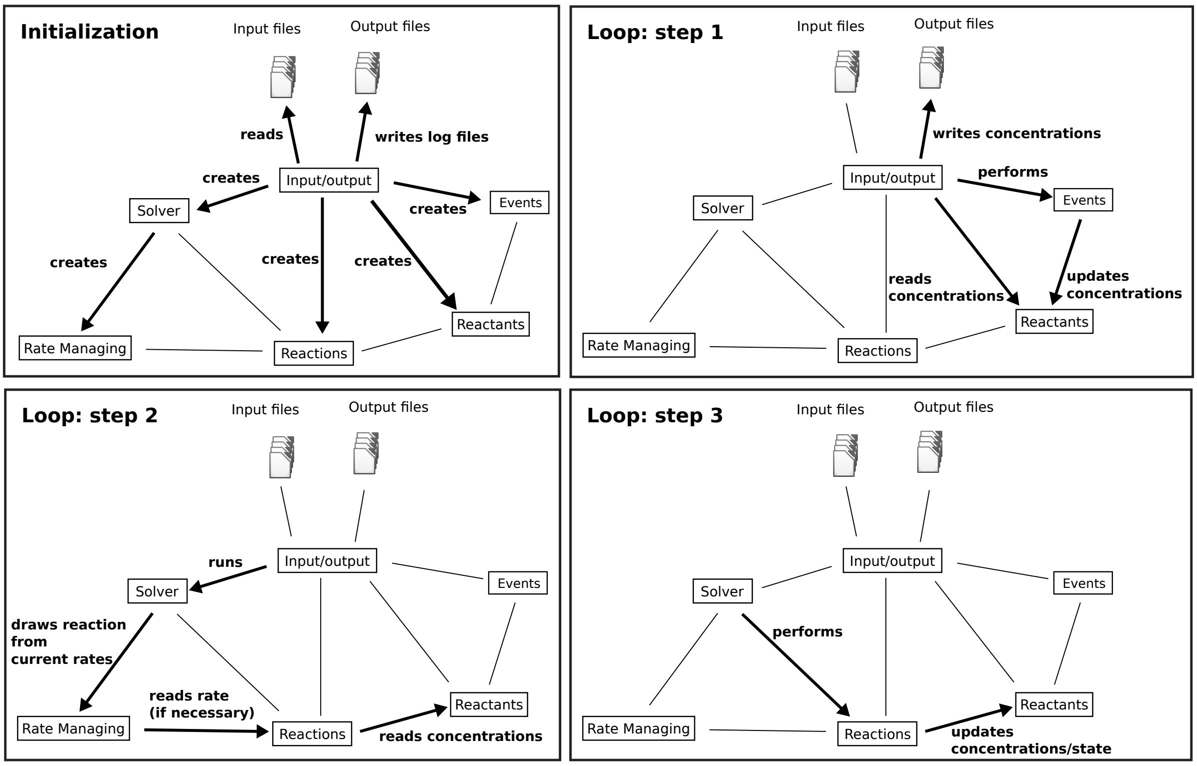

Figure 8: Schematical view of the simulator.

The simulator can be decomposed into several large modules that handle specific tasks during simulation (Fig. 8). First of all, there is an **input/output** module that creates everything that is needed for the simulation from an input file. **Reactants** and **reactions** are user-specified and need to be created on demand, as well as **events** happening throughout the simulations and more technical aspects about which algorithm to use to perform the integration. Once everything is set up, the **solver** follows a simple loop that can be decomposed in three steps. Integration occurs reaction by reaction, at each loop, we go forward one reaction, update the simulation time, concentrations and reaction rates.

1. At the beginning of the loop, the **input/output** process checks whether **events**

should occur at the current simulation time and whether it needs to write some concentrations to an output file.

2. It then hands control over to the **solver**, which is based on Gillespie's approach to integrate a network of chemical reactions. The Gillespie algorithm needs the current reaction rates of all **reactions** and draws a random reaction with a probability proportional to its rate. This task is delegated to a **rate manager**, which uses state-of-the-art methods to maintain the rate list updated and perform the drawing efficiently.

3. Once a **reaction** is drawn, it is performed *i.e.* the concentrations (and the state, see below) of its **reactants** is modified.

4.2 Reactant hierarchy

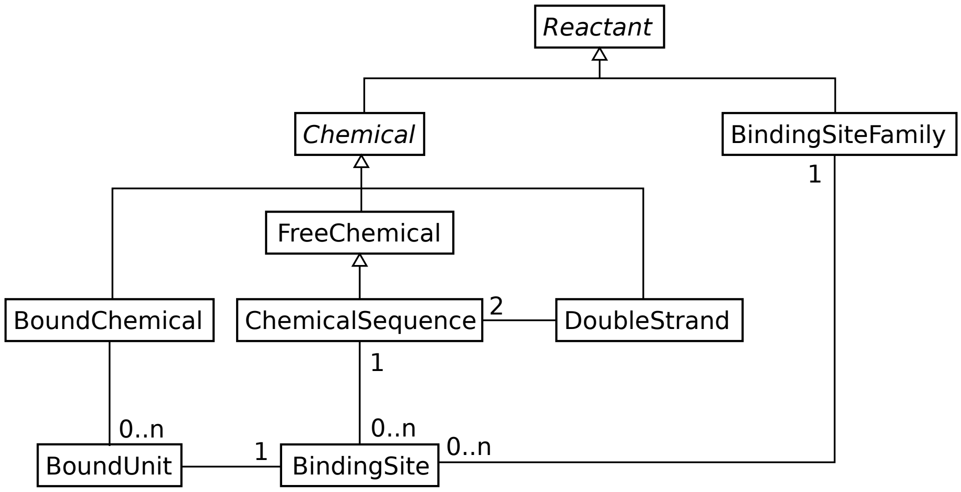

Figure 9: UML diagram of `Reactant` hierarchy

The following sections give a quick overview of the contents of the `Reactant` hirarchy (Fig. 9). More details about how reactants are implemented can be found later.

4.2.1 Reactant

`Reactant` is a global abstract interface. All entities that can participate in a reaction *must* inherit from it.

4.2.2 Chemical

`Chemical` is an abstract class (Fig. 10). It defines all standard chemical entities. `Chemical` represents a *pool* of a given chemical species, meaning that one may access its current number at any time.

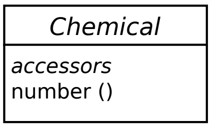

Figure 10: `Chemical` class

4.2.3 FreeChemical

Input format

```
FreeChemical <name> [<initial quantity>]
```

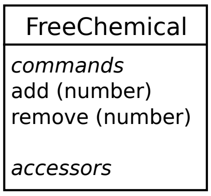

Figure 11: `FreeChemical` class

`FreeChemical` (Fig. 11) is a subclass of `Chemical` that represents free chemical (*e.g.* molecules diffusing in the cytosol or extracellular medium).

4.2.4 BoundChemical

Input format

```
BoundChemical <name>
```

`BoundChemical` (Fig. 12) is a subclass of `Chemical` that represents chemicals that are bound to a sequence. It is important to note it only represents molecules bound to the sequence, *not* the complex formed by the chemical and the sequence. Even though `BoundChemical` represents a pool of molecules, single elements are not interchangeable, they are defined by their position on a sequence. `BoundChemical` uses class `BoundUnit` to represent molecules individually. It uses `BoundUnitFilter` to organize bound units according to outside criteria needed for reactions (classify according to binding sites, motifs read, etc.). It also uses `Switch`es on specific switch sites that are sequence dependent (this will be explained in detail later).

4.2.5 ChemicalSequence

Input format

```
ChemicalSequence <name> sequence <sequence> [<initial quantity>]
TransformationTable <name> [<parent_letter> <product_letter>,]^{1..n}
```

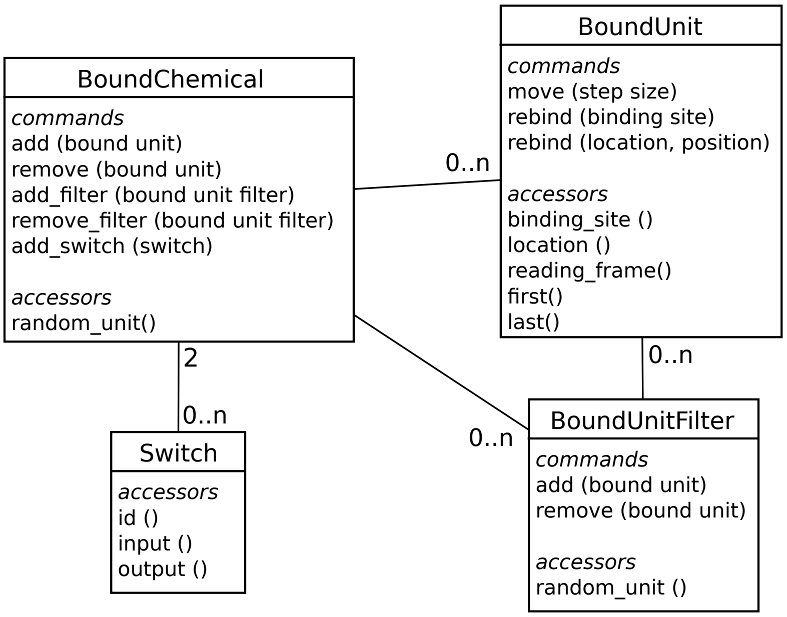

Figure 12: `BoundChemical` class

```
ProductTable <name> <transformation table>
ChemicalSequence <name> product_of <parent sequence> \
  <starting position> <ending position> <product table> [<initial quantity>]
```

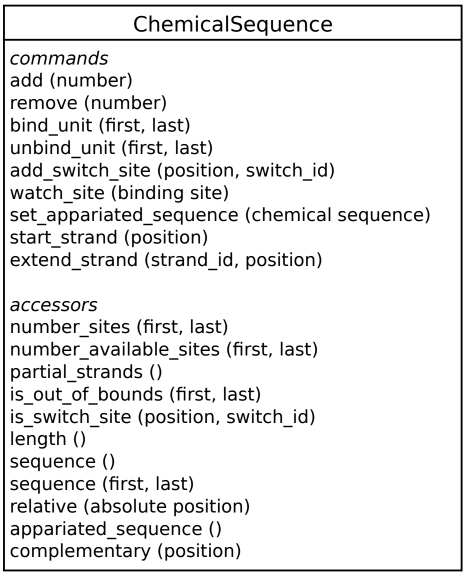

Figure 13: `ChemicalSequence` class

`ChemicalSequence` (Fig. 13) is a subclass of `FreeChemical`. It is defined by a sequence and the ability to bind elements. However, instances of a sequence are *not* treated individually, it is impossible to tell to which instance a given chemical bound. An

object called SequenceOccupation maintains occupation levels at sites of interest. For example, suppose the sequence is an mRNA carrying a ribosome binding site for the protein DnaA. The number of available sites is obtained by removing the number of bound chemicals occupying the site from the number of instances of the mRNA currently in the cell. A ChemicalSequence can be appariated to another ChemicalSequence. A ChemicalSequence can be created from a sequence or as a product of another sequence, in which case a TransformationTable is needed to generate the product's sequence from the parent's, and a ProductTable stores the parent/product relationship.

4.2.6 DoubleStrand

Input format

```
TransformationTable <name> [<letter> <complementary_letter>,]^{1..n}
DoubleStrandSequence <name> <name_sense_sequence> <sense_sequence> \
  <name_antisense_sequence> <transformation_table> [<initial quantity>]
```

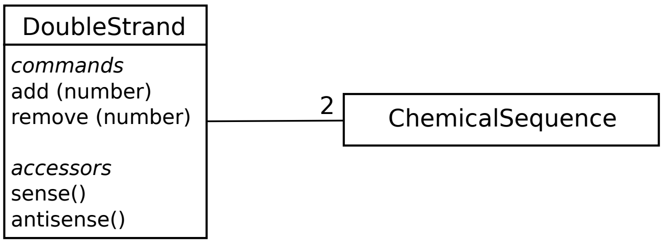

Figure 14: DoubleStrand class

DoubleStrand (Fig. 14) links two ChemicalSequence together that are biochemically linked (*e.g.* DNA), one sequence being complementary to the other. It enables segment extension on the appariated strand and free end binding (see interface of ChemicalSequence). A DoubleStrand is created from a sense sequence that is specified similarly to a ChemicalSequence. However, the complementary sequence is created from a TransformationTable that specifies how to transform the sense sequence into antisense sequence (*e.g.* for DNA, $A \to T$, $T \to A$, $C \to G$, $G \to C$).

4.2.7 BindingSiteFamily

BindingSiteFamily (Fig. 15) is a subclass of Reactant. Contrary to Chemical, it does not represent a countable pool of molecules. Each family contains a number of related instances of BindingSite (*e.g.* ribosome binding sites). BindingSiteFamily, BindingSite and ChemicalSequence use a notification pattern (via update methods) to dynamically maintain the number of available sites for each binding site as well as binding rates up to date. If a binding site is used to load polymerases, a reading frame should be provided to specify where a polymerase will start reading the sequence after binding.

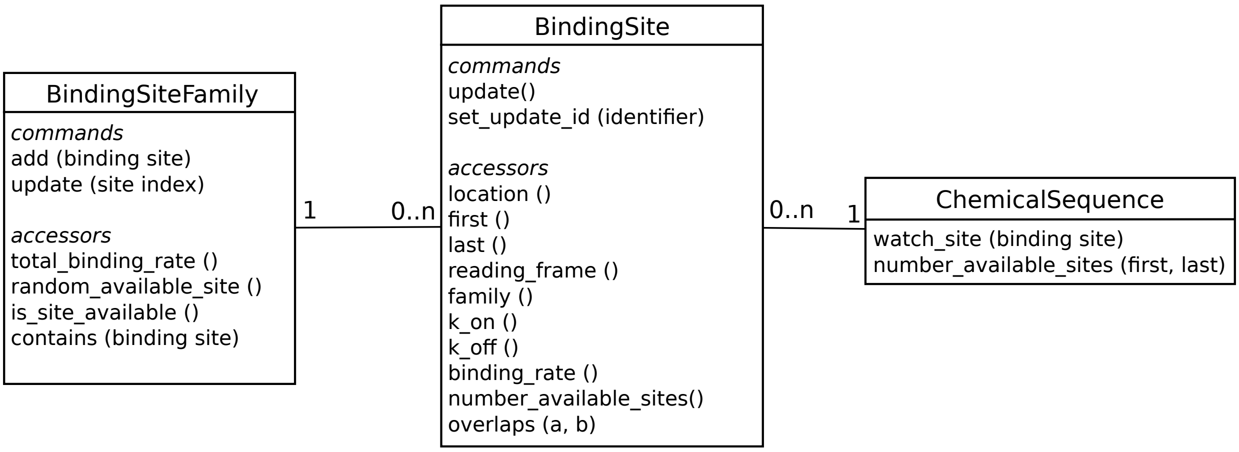

Figure 15: `BindingSiteFamily` class

Input format

```
BindingSite <binding site family name> <chemical sequence> \
  <start> <end> <k_on> <k_off> [<reading frame>]
```

4.3 Reaction hierarchy

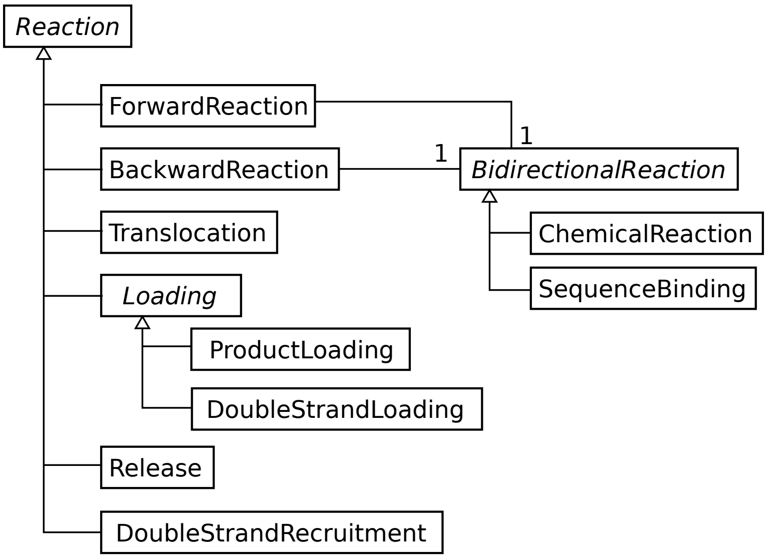

Figure 16: UML diagram of `Reaction` hierarchy.

The following sections gives a quick overview of the reaction hierarchy (Fig. 16). More details about how reactions are implemented can be found later.

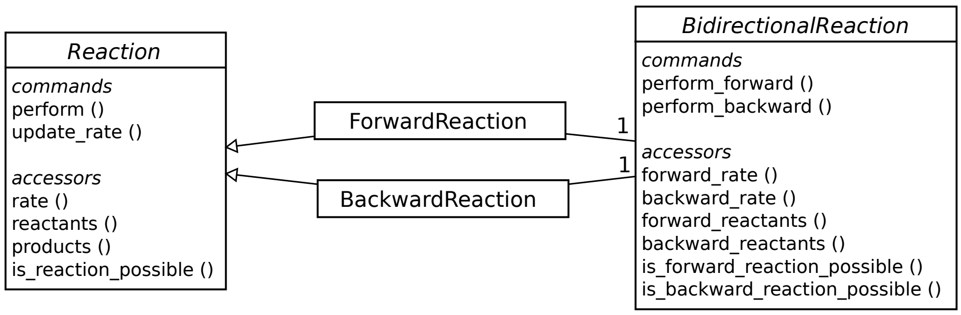

Figure 17: `Reaction` and `BidirectionalReaction` classes.

4.3.1 Reaction

There are two abstract classes used to define reactions: `Reaction` for one-way reactions and `BidirectionalReaction` for reversible reactions. Two adapter classes `ForwardReaction` and `BackwardReaction` split reversible reactions in two one-way reactions(Fig. 17). In the end, the solver only handles one-way reactions. A reaction can necessarily be performed, its rate updated and accessed and is composed of reactants and products.

4.3.2 ChemicalReaction

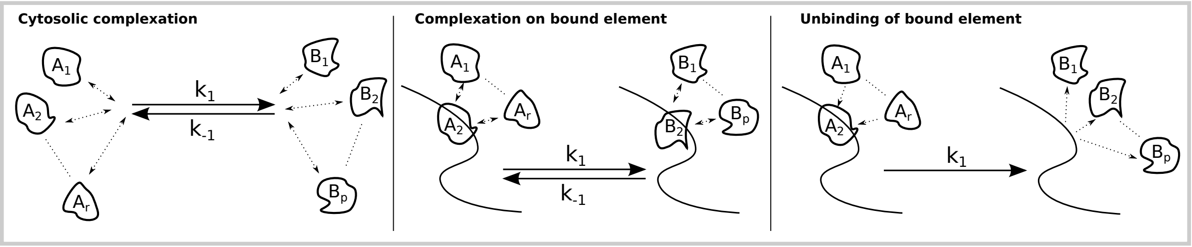

Figure 18: Schematic view of a `ChemicalReaction`.

Input format

```
ChemicalReaction [<chemical> <stoichiometry>]^{1..n} rates <k_1> <k_-1>
```

Formula A `ChemicalReaction` represents association/dissociation of an arbitrary number of elements (Fig. 18). It is defined by

$$a_1A_1 + a_2A_2 + ... + a_rA_r \underset{k_{-1}}{\overset{k_1}{\rightleftharpoons}} b_1B_1 + ... + b_pB_p$$

where

- A_i and B_i are of type `FreeChemical`. They can be of type `BoundChemical` in two cases: (i) a reaction containing a `BoundChemical` on each side, (ii) an *irreversible* reaction where a *reactant* is a `BoundChemical` and where there are no bound product. In both cases, the associated stoichiometric coefficient must be 1.
- a_i and b_i are stoichiometric coefficients.
- k_1 and k_{-1} are rate constants.

Action When the reaction is performed, the number of chemicals involved is changed according to their stoichiometric coefficient. If `BoundChemical` are involved on each side, the simulator will assume that the bound chemical that is consumed is replaced by the bound chemical on the other side of the equation (*i.e.* it will be bound at the location previously occupied by the precursor). If there is a `BoundChemical` on the reactant side of an irreversible reaction, the simulator will assume that the reaction describes the unbinding of this bound unit into the cytosol.

Rate The rates are given by

$$\lambda_{forward} = k_1 \prod_{i=1}^{r} [A_i]^{a_i}$$

$$\lambda_{backward} = k_{-1} \prod_{i=1}^{p} [B_i]^{b_i}$$

4.3.3 SequenceBinding

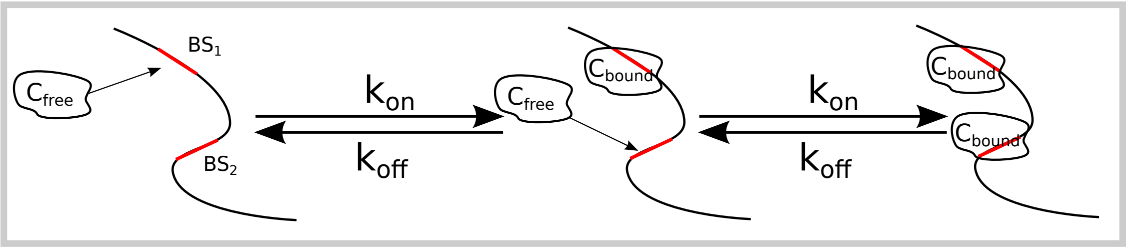

Figure 19: Schematic view of a `SequenceBinding`.

Input format

```
SequenceBinding <chemical> <bound form> <binding site family>
```

Formula A `SequenceBinding` represents binding of a free element on a binding site of a sequence (Fig. 19). It is defined by

$$C_{free} + BSF \rightleftharpoons C_{bound}$$

where

- C_{free} is of type `FreeChemical`.
- BSF is of type `BindingSiteFamily`.
- C_{bound} is of type `BoundChemical`.

Action When the forward reaction is performed, a random available binding site is drawn from the binding site family (drawing is weighted by affinity). A C_{free} molecule is removed from the pool and a C_{bound} added to the `ChemicalSequence` bearing the binding site. When the backward reaction is performed, a random molecule of C_{bound} is removed from the pool (and from its sequence) and a C_{free} molecule is added.

Rate The rates are given by

$$\lambda_{forward} = \frac{[C_{free}]}{V_c} \sum_{\text{sites } s \in BSF} (k_{on})_s \times \text{Number of sites } s \text{ available}$$

$$\lambda_{backward} = \frac{1}{V_c} \sum_{\text{molecules } m \in C_{bound}} (k_{off})_{\text{site on which } m \text{ is bound}}$$

- $(k_{on})_s$ is the association constant of C_{free} with binding site s.
- $(k_{off})_s$ is the dissociation constant of C_{bound} with binding site s.
- V_c is the volume of the cell.

4.3.4 Translocation

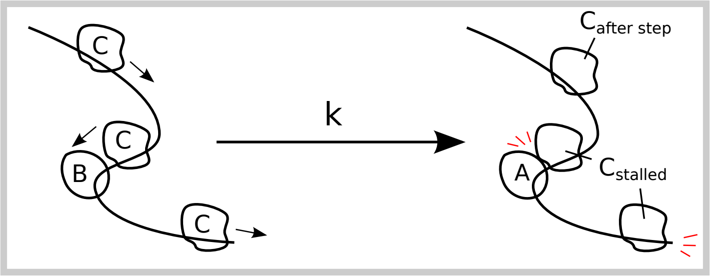

Figure 20: Schematic view of a `Translocation`.

Input format

```
TerminationSite <family name> <chemical sequence> <start> <end>
Translocation <bound chemical> <form after step> <stalled form> \
  <step size> <rate>
```

Formula A `Translocation` represents movement of a bound element along a sequence (Fig. 20). It is defined by

$$C \xrightarrow{k} C_{\text{after step}}$$

or

$$C \xrightarrow{k} C_{\text{stalled form}}$$

where

- C is of type `BoundChemical`.
- $C_{\text{after step}}$ is of type `BoundChemical`.
- $C_{\text{stalled form}}$ is of type `BoundChemical`.
- k is a rate constant.

Action When the reaction is performed, a random C is chosen. Generally, it is replaced by a $C_{\text{after step}}$, moved by a step of a given size along the sequence the original C is bound to. If the chemical cannot move because it reached the end of the sequence, it is replaced by $C_{\text{stalled form}}$.

Rate The rate is given by

$$\lambda = k[C]$$

4.3.5 Loading

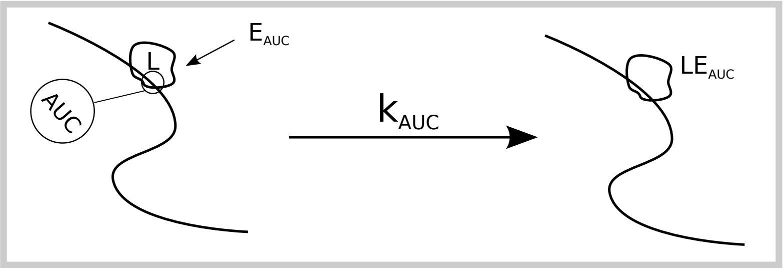

Figure 21: Schematic view of a `Loading`.

Input format

```
LoadingTable <name> \
  [<template> <element_to_load> <occupied_polymerase> <rate>,]^{1..n}
ProductLoading <bound chemical> <loading table>
DoubleStrandLoading <bound chemical> <loading table> <stalled form>
```

Formula A `Loading` typically represents loading of elements by a polymerase onto a template sequence (Fig. 21). It is defined by

$$L + E \longrightarrow LE$$

where

- L is of type `BoundChemical`.
- E is an element to load, of type `FreeChemical`. It is defined in a `LoadingTable` associated with the reaction.
- LE is the occupied form of the loader, of type `BoundChemical`. It is defined in a `LoadingTable` associated with the reaction.

Action Each instance of L reads a specific template. Using its `LoadingTable`, we know which E it tries to load, which LE is yielded if loading occurs and the loading rate associated with the template. When the reaction is performed, a random L is chosen according to loading rates. An element to load E is removed from the pool and L is replaced with LE. A `ProductLoading` assembles loaded elements into a product that will eventually be release in the cytosol (*e.g.* RNA synthesis), while `DoubleStrandLoading` extends segments along a `DoubleStrand` (*e.g.* DNA replication). In `DoubleStrandLoading`, loading may fail because the loader met a previously synthesized segment. In the latter case, it is replaced by a `BoundChemical` representing its stalled form.

Rate The rate is given by

$$\lambda = \sum_{t \in templates} k_t [L_t][E_t]$$

where

- k_t is the loading rate associated with template t.
- L_t corresponds to loaders L reading template t.
- E_t is the chemical to load onto template t.

4.3.6 DoubleStrandRecruitment

Input format

```
DoubleStrandRecruitment <BoundChemical> <FreeChemical> <bound form> <rate>
```

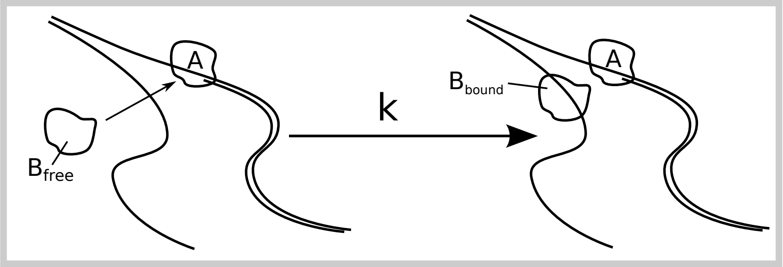

Figure 22: Schematic view of a `DoubleStrandRecruitment`.

Formula A `DoubleStrandRecruitment` typically represents recruitment of a DNA polymerase by the replication fork on the opposite strand (Fig. 22). It is defined by

$$A + B_{free} \xrightarrow{k} A + B_{bound}$$

where

- A is of type `BoundChemical`, bound to a `DoubleStrand`.
- B_{free} is of type `FreeChemical`.
- B_{bound} is a `BoundChemical` representing the bound form of B_{free}.
- k is a rate constant.

Action When the reaction is performed, a random A is chosen. If A is not bound to a `DoubleStrand`, the reaction is ignored. If the position opposite to a on the `DoubleStrand` is already occupied, the reaction is ignored. Else, a B_{free} is bound on the complementary `ChemicalSequence`, opposite to A as a B_{bound}.

Rate The rate is given by

$$\lambda = k[A][B_{free}]$$

4.3.7 Release

Input format

```
TransformationTable <name> [<parent_letter> <product_letter>,]^{1..n}
ProductTable <name> <transformation table>
Release <polymerase> <empty_polymerase> <fail_polymerase> \
  <product table> <rate>
```

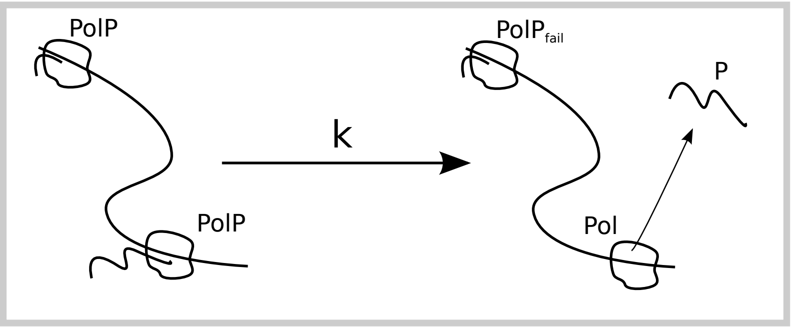

Figure 23: Schematic view of a `Release`.

Formula A `Release` represents release of a product from a polymerase (Fig. 23).

$$PolP \xrightarrow{k} Pol + P$$

or

$$PolP \xrightarrow{k} PolP_{fail}$$

where

- $PolP$ is a `BoundChemical` representing a polymerase-product complex.
- P is of type `ChemicalSequence`. It is a product that is released by $PolP$ defined in a `ProductTable` associated with reaction.
- Pol is a `BoundChemical` representing an empty polymerase.
- $PolP_{fail}$ is a `BoundChemical` representing the polymerase-product complex in case release failed because P was not a valid product defined in the `ProductTable` associated with reaction.
- k is a rate constant.

Action When the reaction is performed, a random $PolP$ is chosen. A `ProductTable` uses its binding and current position to determine what product P it has synthesized. If P is defined in the product table, it is released in the cytosol and $PolP$ is replaced by an empty version of the polymerase Pol. If there is no P corresponding to current $PolP$ position, the simulator assumes that $PolP$ has not reached its actual terminator and it is replaced by $PolP_{fail}$ to enable other treatments (*e.g.* abnormal termination or continuing synthesis).

Rate The rate is given by

$$\lambda = k[PolP]$$

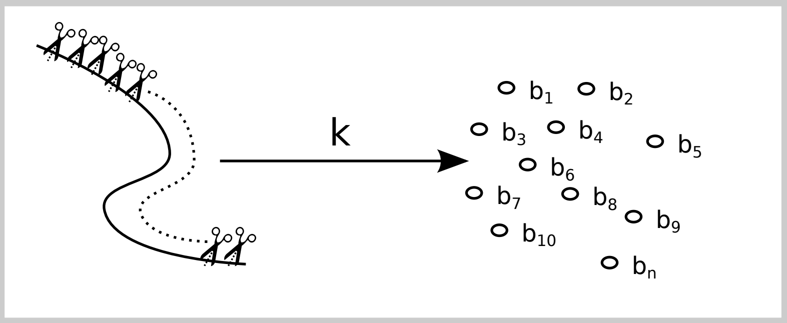

Figure 24: Schematic view of degradation reaction.

4.3.8 Degradation

Input format

```
CompositionTable <name> [<letter> [<chemical composing letter>]^{1..m}]^{1..n}
Degradation <chemical sequence> <composition table> <rate>
```

Formula A `Degradation` represents decomposition of a sequence into base components (Fig. 24). It is defined by

$$CS \xrightarrow{k} b_1 + b_2 + ... + b_N$$

where

- CS is of type `ChemicalSequence`.
- b_i are of type `FreeChemical`. They are found in a `CompositionTable` specified in the reaction.
- k is the degradation constant.

Action When the reaction is performed, a CS is removed from the pool. A `CompositionTable` is specified along the reaction. It allows base-by-base conversion of the sequence of CS into components yielded by degradation. The pools of base components is updated accordingly. In the simulator, a degradation reaction is effectively implemented as a `ChemicalReaction`.

Rate The rate is given by

$$\lambda = k[CS]$$

4.3.9 Switches

Input format

```
Switch <name> <input_bound_chemical> <output_bound_chemical>
SwitchSite <chemical_sequence> <position> <switch_name>
```

`Switch`es are intrinsically linked to `BoundChemical`s but apply to specific `BoundUnit`s through `SwitchSite`s located on `ChemicalSequence`s. Every time an instance of `input_bound_chemica` steps on a switch site, it *immediately* becomes an `output_bound_chemical`.

A `Switch` is not considered a reaction because there is no rate associated with it (the solver does not actually know anything about switches). We dedicate a section to these elements because they play a central role in the simulator's philosophy. The user can use generic reactions that apply in general (*e.g.* transcription of any gene based on its sequence) and use switches every time something more specific is needed. Typically, termination sites for transcription are expected to be *SwitchSite*s. Similarly, important regulation sites can be implemented using *SwitchSite*s.

4.3.10 Solver loop

Once `Reactions` and `Reactant`s are defined, they must be integrated properly. We use variants of the Gillespie algorithm to provide a framework where reactions are performed according to their current reaction rate. Roughly speaking, the main hypothesis of this framework is that reaction timings are distributed according to exponential distributions. This allows for many mathematical simplifications and harmonious integration of an arbitrary number of reactions. The central point of the algorithm is that the probability that a reaction will be the next reaction in the system is proportional to its rate (mathematically speaking, the reaction is obtained by multinomial drawing according to rates).

The solving loop is depicted in Figure 25. The Gillespie algorithm has many variants. We decided to implement it using three *abstract* classes. By using inheritance, variants can be combined for each step of the algorithm (how to update reactions, how to select a reaction). The three central classes are:

- `Solver`: Children of this class decide how and when rates should be updated, *e.g.* update rates after every reaction, only after a given time step, etc. Note that they do not perform any of these computations, they just organize how the algorithm should work.

- `RateManager`: Children of this class are responsible for updating reaction rates when prompted to by a `Solver` class. Recomputing all rates is generally inefficient, so various implementations of this task can be used to improve the global loop speed.

- `RateContainer`: Childern of this class are responsible for storing reaction rates in a specific structure *adapted* to multinomial drawing. Again many implementations exist, their efficiency depends on the system that is integrated.

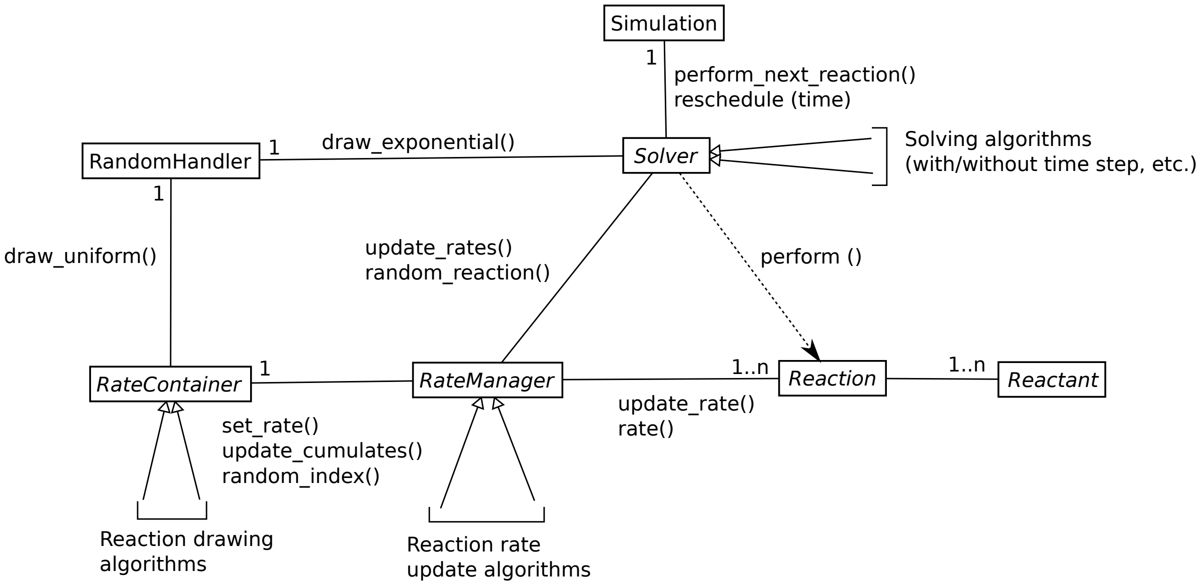

Figure 25: Solver loop. The loop is driven by the `Solver` class that defines how and when rates should be updated. The update task is performed by a `RateManager`. Once rates are known, multinomial drawing is delegated to a `RateContainer`. A central `RandomHandler` is used so that the solver only uses one seed, enabling simulation reproducibility.

The implementations of these three classes will be described later in the document.

4.3.11 Events

`Events` enable users to change molecule numbers outside of the solver loop at specific times (Fig. 26). A `Simulation` instance handles both a `Solver` instance and an `EventHandler` instance. Every time an event timing is reached, the solver loop is stopped, the event(s) is (are) performed, the solver is reinitialized and the simulation resumes. Different `Event` implementations are offered to modify molecule numbers in a convenient way.

4.3.12 Input/Output handling

Simulator Input The simulator needs the following to work:

- A general input file defining simulation parameters. A sample file is provided were all options are described (*e.g.* length of simulation, what to output, algorithm variants). One important parameter is the location of the files the simulator should open to read reactants, reactions and events.
- An arbitrary number of files where reactants, reactions and events are declared. The simulator solves dependencies across files, it is not necessary to declare reactants in the same file as or before reactions using them.

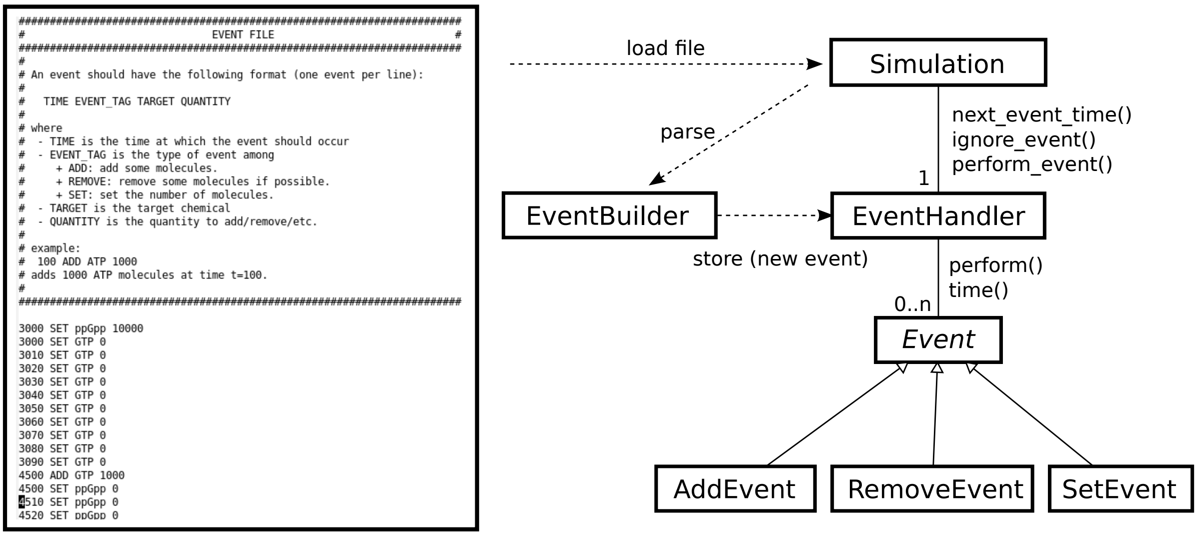

Figure 26: `Events`: another way to modify chemical concentrations aside from reactions, *e.g.* to simulate the injection of a chemical inside a cell.

Caution:

- All reactants must be declared in some file with their appropriate type (*e.g.* `FreeChemical` or `BoundChemical`).
- Multiple declarations are forbidden, a name cannot be reused.

Simulator Output Outputs provided by the simulator are:

- A general output file logging parametes used for simulation (input files used, random seed, algorithms used, etc.).
- A concentration file with the number of molecules over time (for the chemicals and at a time step defined in the parameter file).
- If a `DoubleStrand` was added in the chemicals to ouput, a replication file describing replication advancement of that `DoubleStrand`.

4.4 Detailed design

4.4.1 Reactants

`FreeChemical` `FreeChemical` simply represents a pool of interchangeable molecules distributed uniformly in the cell. Computationnally, only the number of molecules in the pool is relevant.

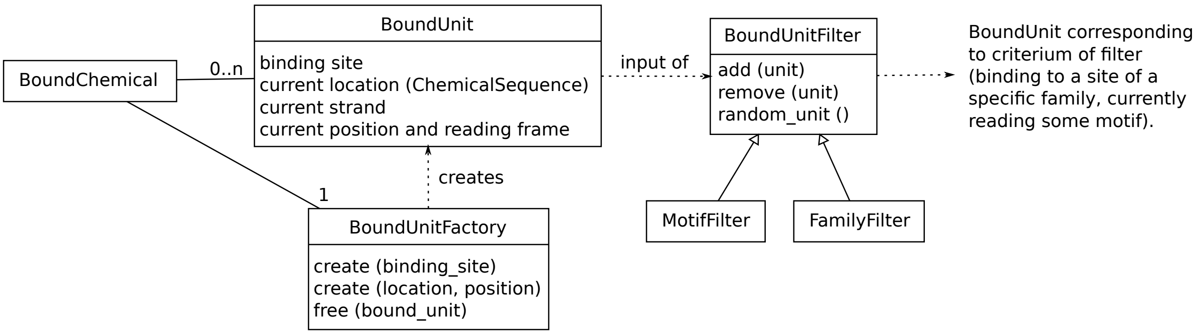

Figure 27: `BoundChemical` are in fact a pool of individual `BoundUnit` created using a `BoundUnitFactory`. A `BoundUnit` is characterized by the `ChemicalSequence` it bound to and its current position. Reaction then use `BoundUnitFilter` to sort `BoundUnit` according to some criterium of reference (*e.g.* `Loading` reactions sort `BoundUnit` according to the motif they read).

`BoundChemical` `BoundChemical` represents molecules of the same chemical species, but there are specifities for each unit of a `BoundChemical`, as all units are bound at different locations of different `ChemicalSequence` (Fig. 27). A `BoundUnitFactory` is used to recycle `BoundUnits`, avoiding memory reallocation throughout simulation. `BoundUnitFilters` are used to sort `BoundUnits` according to criteria useful for reactions (Fig. 27).

`BoundUnits` are passed from one `BoundChemical` species to another through reactions, their attributes are updated if needed. They are only destroyed once they are unbound from their `ChemicalSequence`.

`ChemicalSequence` `ChemicalSequence` handles a pool of polymers. A pool is defined by a *master sequence* describing what a typical polymer looks like (*e.g.* the sequence of DnaA protein) and the number of *instances* of the master sequence in the pool. For efficiency reason, we do the following assumptions.

Simplifying assumptions

- No deviation from master sequence, all instances are identical.
- `BoundUnits` are not assigned to a specific instance of the sequence, they are positioned on the master sequence.

Consequences

- No direct inference of collisions is possible.
- A chemical can bind on a partial strand, yet move along the whole sequence freely.
- Degradation of an instance does not cause unbinding.

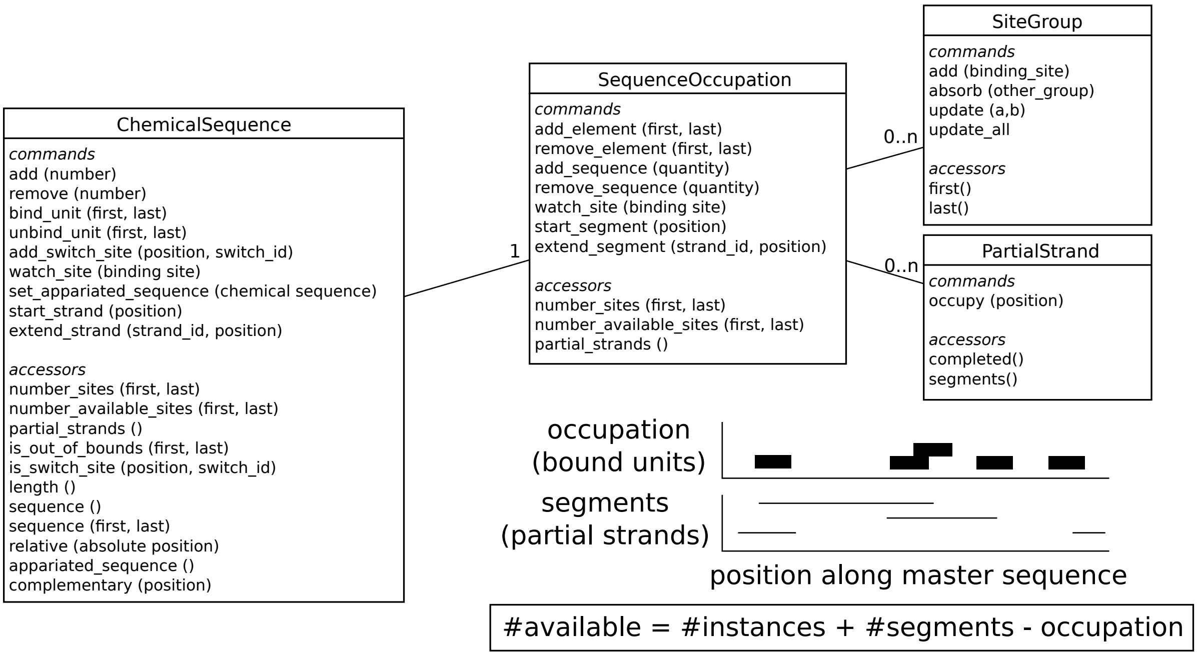

Figure 28: `ChemicalSequence` represents a pool of polymeres that can be elongated and on which `BoundUnits` bind through `BindingSites`. For binding to occur, availability of `BindingSite` is assessed using a utility class `SequenceOccupation` that records the number of instances of the polymer, the position of `BoundUnits` and elongation of `PartialStrands`. `SiteGroup` is used to notify sites of availability changes more efficiently.

Site availability Despite our simplifying assumptions it is still possible to provide an accurate description of site availability. Availability depends of the number of sequences, number and position of bound elements, number and position of newly polymerized sequence segments (Fig. 28).

`DoubleStrand`

Strand identification Because `DoubleStrand` typically reprensents DNA, we expect that the `DoubleStrand` will contain a lot of `PartialStrands`. For replication, it is important to know exactly which strand are opposite to one another for `DoubleStrandRecruitment` to work properly. We use strand identification as shown in Figure 29.

`BindingSiteFamily` The task of a `BindingSiteFamily` is to regroup all the binding sites that can participate in a same `SequenceBinding` reaction. To simplify the reaction, it stores the subrate associated with each binding site. In order to update the rate properly when availability of sites changes, an *observer pattern* is used (Fig. 30).

Every `BindingSite` is viewed as an *observer* by the `ChemicalSequence` it belongs to. Every time a change occurs on the site, the `BindingSite` is notified. The latter binding

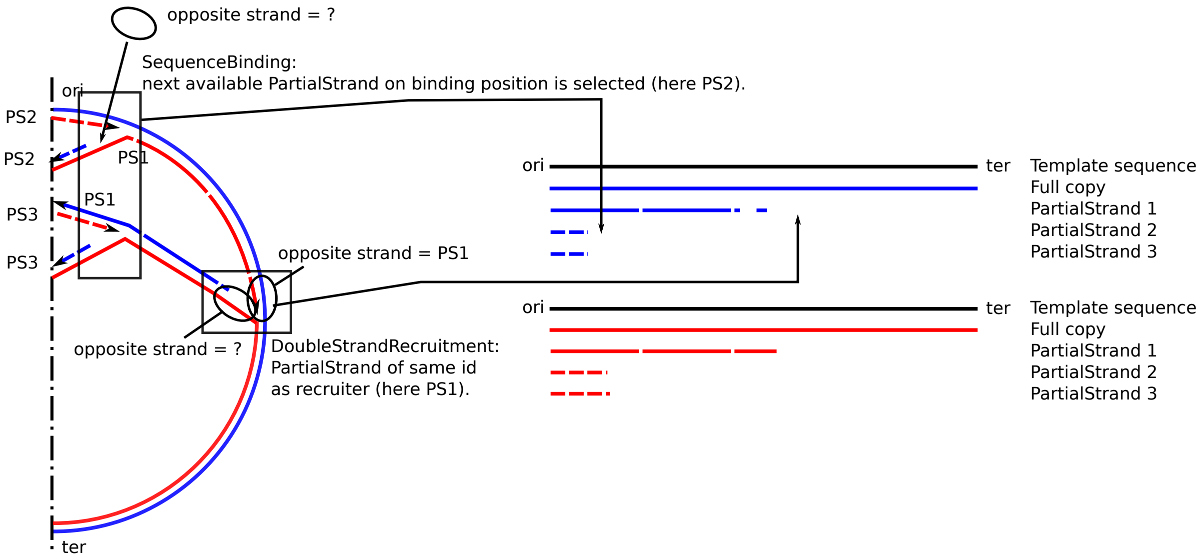

Figure 29: Strands of a `DoubleStrand` are identified according to creation order. Every time a new segment is polymerized, it is necessary to determine which `PartialStrand` is elongated. If a polymerase has been recruited on the complementary strand by `DoubleStrandRecruitment`, it is automatically assigned the same partial strand as the recruiter.

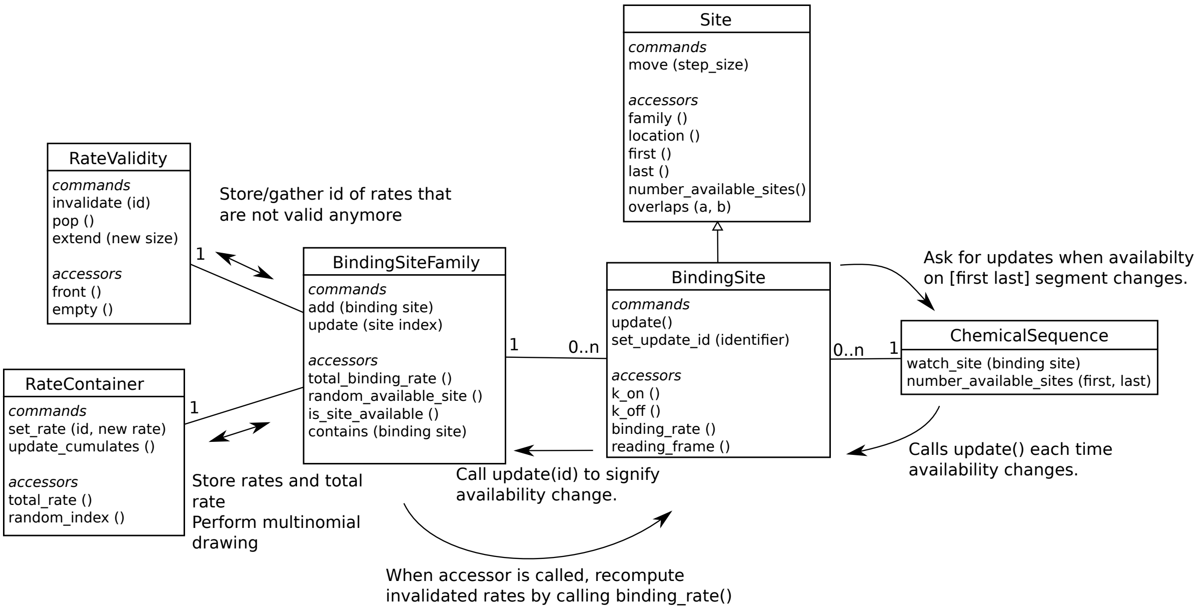

Figure 30: Schematical view of the Observer pattern used to keep availability of binding sites up to date for `SequenceBinding` reactions.

site notifies its `BindingSiteFamily` using a specific identifier, letting the family know which binding rate is out of date. This information is stored in a `RateValidity` class. It is only when it is really needed (*i.e.* when a `SequenceBinding` wants to access total rate

or a random site) that rates are recomputed. This avoids useless computations *e.g.* in the case of a translocation, where a bound unit is first unbound from its `ChemicalSequence` then rebound. If the bound unit does not move away from the site, two updates will be sent, but the rate will only be recomputed once at the end.

4.4.2 Reactions

`ChemicalReaction` Nothing particular.

`SequenceBinding`

Binding Because of the way `BindingSiteFamily` is implemented, the reaction can easily and efficiently access the binding rate at all times, no matter what reactions have occured previously and how site availability changed in the meantime.

Unbinding `SequenceBinding` uses a `FamilyFilter` (see detailed description of `BoundChemical`) to filter out all `BoundUnits` that are bound to a binding site of the `BindingSiteFamily` associated with the reaction. `BoundUnits` that have bound to sites of a different family or that have moved away from the binding site through `Translocation` are *not* candidates for unbiding.

`Translocation`

Collisions For now, `Translocation` ignores collisions, making its implementation straightforward.

Stalled form Translocation enters stalled form if a `BoundUnit` reached the end of a sequence.

`Loading`

Handling each polymerase individually The main challenge with `Loading` is to maintain the subrates associated with each motif up to date. It needs to maintain a list of all `BoundUnits` reading a specifing motif. To this end it uses a `TemplateFilter` (see detailed implementation of `BoundChemical`). Every time a `BoundUnit` becomes of the type of the `BoundChemical` associated with the reaction, the filter looks what motif defined in the `LoadingTable` it is currently reading. If the motif could not be found, an `UNKNOWN TEMPLATE` error message is displayed, the `BoundUnit` is not recorded in the filter and will not participate in the `Loading` reaction. The implementation is very similar to that used for `BindingSiteFamily` (Fig. 31).

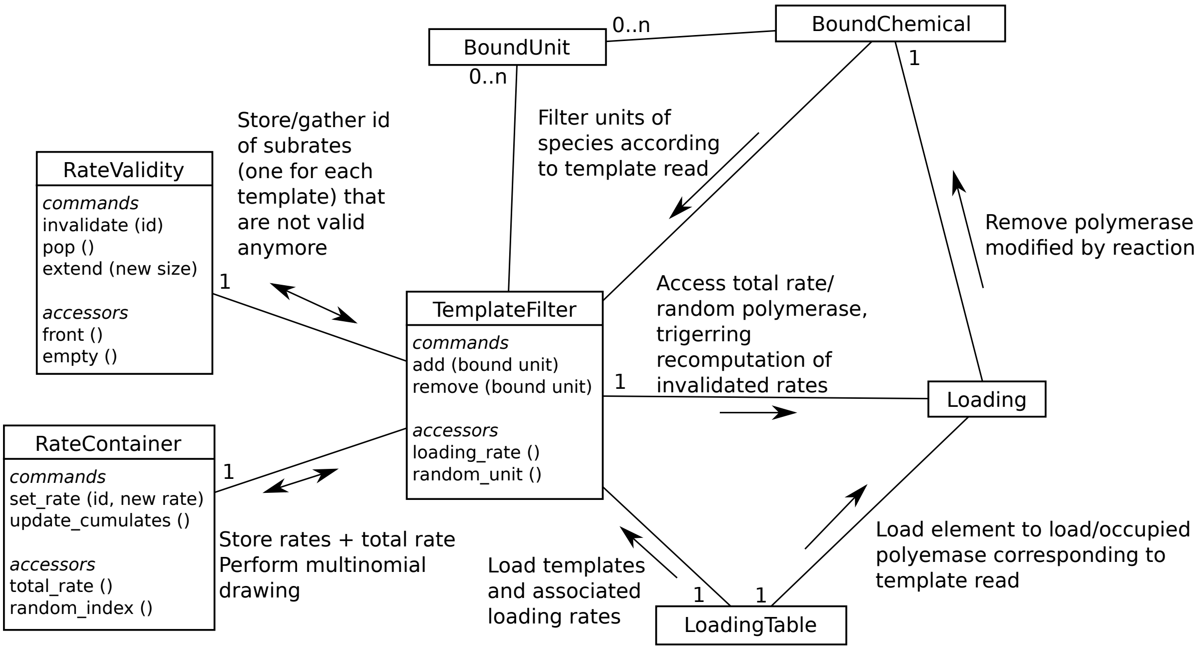

Figure 31: Schematical view of the pattern used to keep subrates associated with each template up to date in a `Loading` reaction.

`ProductLoading` vs `DoubleStrandLoading` There difference between the two processes is rather small. We just added a failure condition in the case of `DoubleStrandLoading` for convenience. Depending on what reactions are used to synthesize a `DoubleStrand` it might be possible that a polymerase arrives upon a position that has already been synthesized. In this case, the `DoubleStrandLoading` fails and the polymerase is replaced by the polymerase in its stalled form.

`Release`

Fail polymerase (unknown product) When a release is triggered, a `BoundUnit` from the `BoundChemical` associated with the `Release` reaction is randomly chosen. Because the `BoundUnit` knows its current position and its binding site, it will assume that product it has synthesized starts the *reading frame of the binding site* and ends *at the position directly preceding its current reading frame* (we assume that the polymerase translocates onto a terminating sequence which does not contribute to product synthesis). If the product is found in the `ProductTable`, everything works normally.

If the product is not found, we display a `Unknown Product` error message but keep the simulation alive. The fail polymerase in the reaction enables the user to define a rescue pathway. If the release competes with some other reaction for the original polymerase, the fail polymerase can be the original polymerase itself. If products overlap and the polymerase was stalled due to a termination site of another product, fail polymerase can be a polymerase in a sythesizing step (*e.g.* `ProductLoading`) so synthesis will resume until the next termination site is reached.

4.4.3 Solver loop

Here we describe the implementations provided for each step of the algorithm. Most of the details are explained in Section 5. We only give a quick overview here.

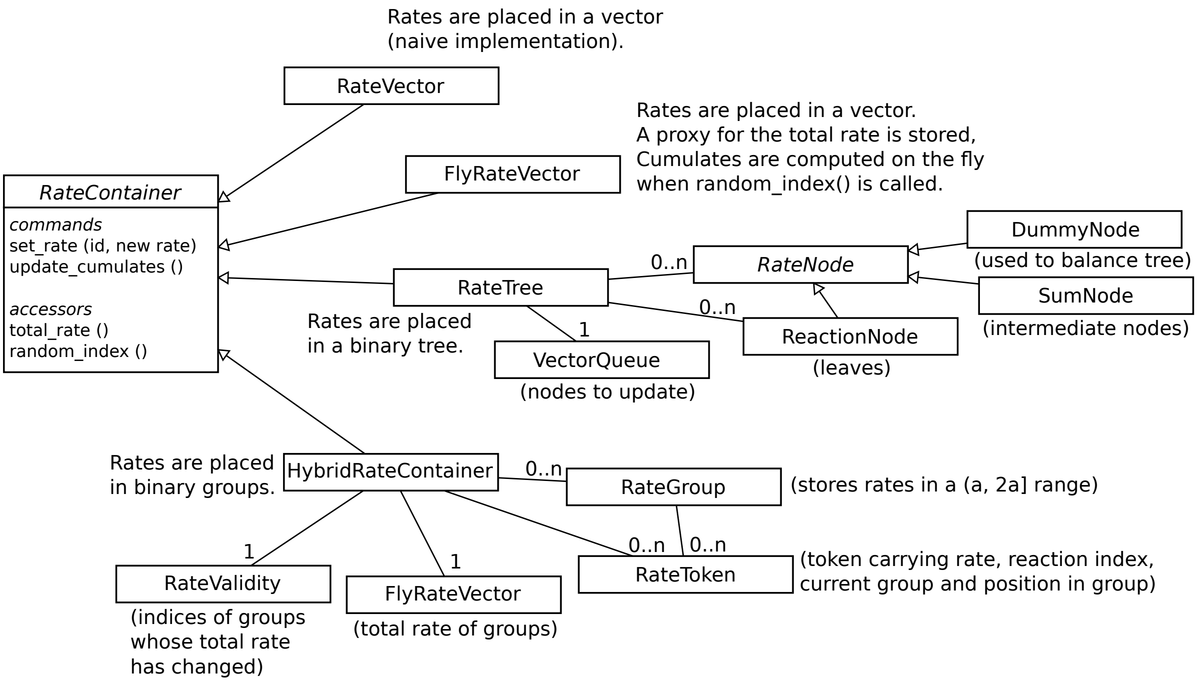

Figure 32: Implementations provided to store rates and perform a multinomial drawing. Implicitly, all theses classes use `RandomHandler` to perform their random drawings.

`RateContainer` **classes** We start with the lowest level classes, which perform one of the central tasks of the Gillespie algorithm: drawing a reaction from reaction rates. For efficiency reasons, we propopose several implementation of the algorithm (Fig. 32). Comparison and description of theses classes are given in Section 5.

Note that multinomial drawing occurs within the solver loop, but also within some reactions such as `Loading` or `SequenceBinding`, so these classes are used quite extensively throughout the simulation.

`RateManager` **classes** The second layer of the solver loop ensures that the rates are updated when needed to. Two implementations are proposed for this task (Fig. 33). The `NaiveRateManager` updates every rate. While it is inefficient, it can be used as a reference to test other managers. The `DependencyRateManager` uses an observer pattern to update only reactions for which a reactant concentration has changed (see Section 5 for further details).

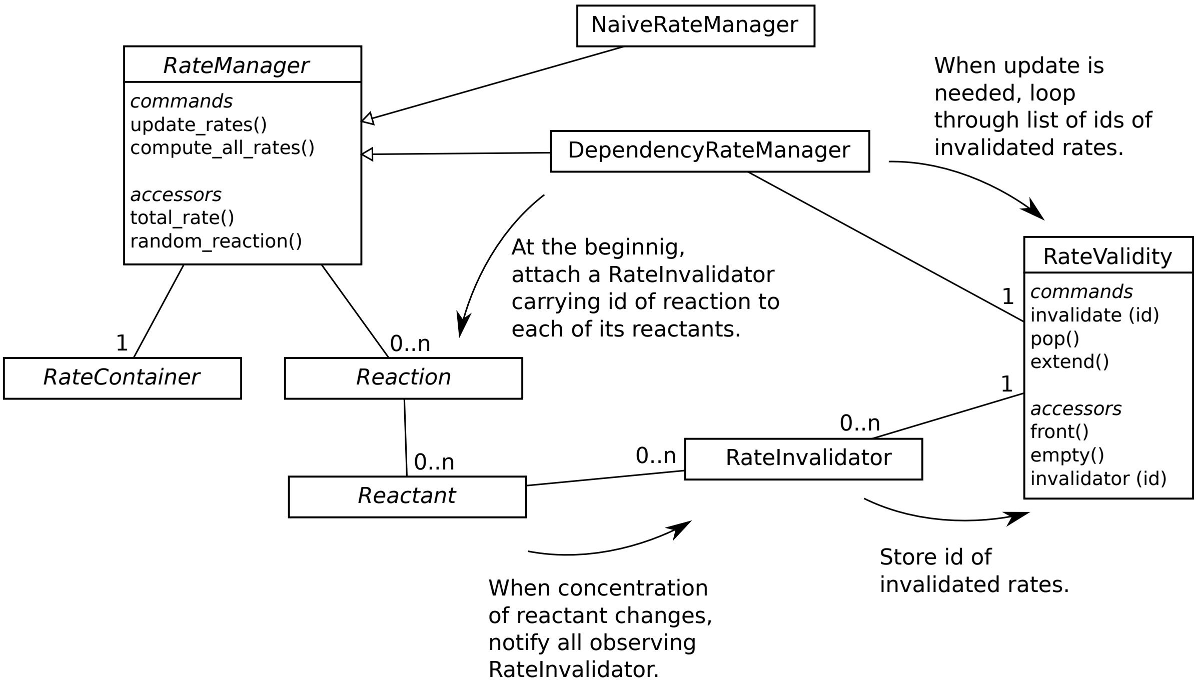

Figure 33: Implementations provided to update reaction rates. Note that the drawing part of the algorithm is always delegated to a `RateContainer`. `DependencyRateManager` uses an Observer pattern to monitor which rates have changed.

4.5 Formats and Conventions

4.5.1 Input format description

- A plain word indicates a tag, that needs to be written.
- `<...>` indicates a variable that has to be completed with an existent element of the specified type.
- `[...]` indicates an optional part.
- `[...]^{0..n}` indicates an optional part that can be repeated an arbitrary number of times.
- `[...]^{1..n}` indicates an part that can be repeated an arbitrary number of times, at least once.
- `[...,]^{0/1..n}` indicates a part that can be repeated an arbitrary number of times, each repetition being separated by a `,` (*but there is actually no* `,` *after the last repetition*).

4.5.2 UML

Figure 34 shows the conventions used in UML diagrams.

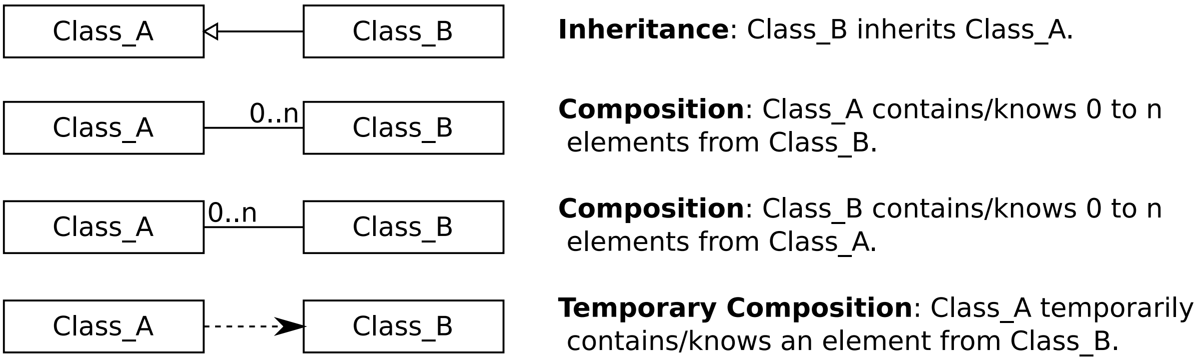

Figure 34: UML format used.

5 Implementation of Gillespie's Stochatic Simulation Algorithm

Gillespie's algorithm was designed to simulate chemical reaction networks, even in conditions where there are only a few molecules. Gillespie et al. (2013) propose a good review of the original algorithm and some of its variants. The original SSA algorithm that can be summarized as follows:

- STEP 1: Update propensity functions.
- STEP 2: Select reaction to perform and next reaction time, perform reaction.

5.1 SSA variants implemented in BiPSim

5.1.1 Direct method

Principle The first method that was used historically is staightforward and sometimes referred to as *biased wheel*. Schematically speaking, one could imagine a wheel similar to "wheel of fortune", except the size allowed to each index on the wheel is proportional to its propensity value, so that large value have a larger probability to be drawn when the wheel is spinned (Fig. 35).

Generally the wheel is seen as a segment subdivided into N subsegments of length r_1, ..., r_N. A value u is drawn on this $[0, \sum r_i)$ segment. We proceed iteratively to find to which subsegment u belongs. If $u < r_1$, it belongs to subsegment 1. If $r_1 \leq u < r_1 + r_2$, it belongs to subsegment 2, etc.

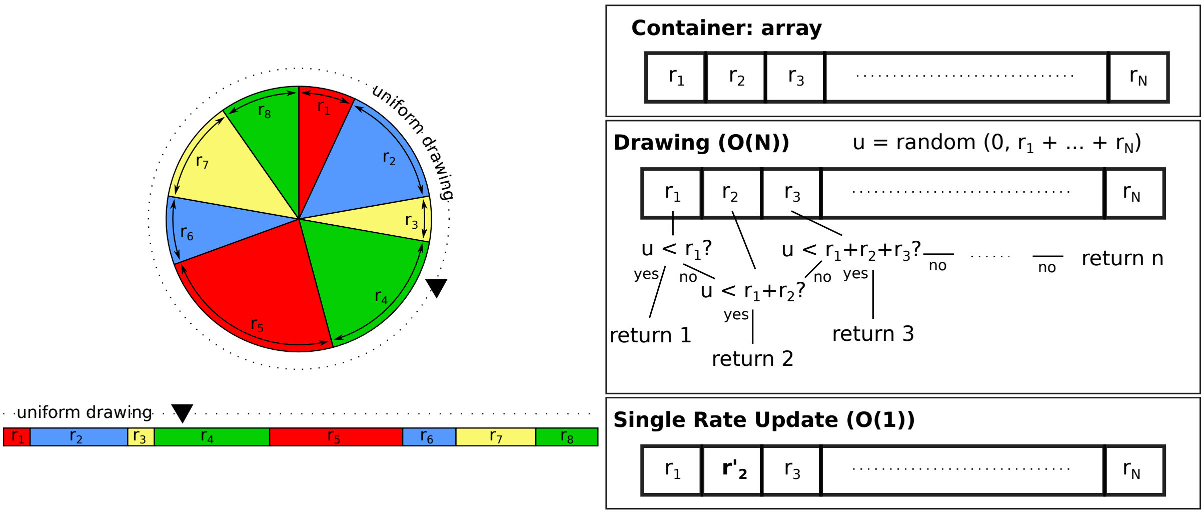

Figure 35: (Left) Illustration of drawing along a biased wheel and its equivalent representation as a segment. (Right) Container and algorithms used to maintain the structure.

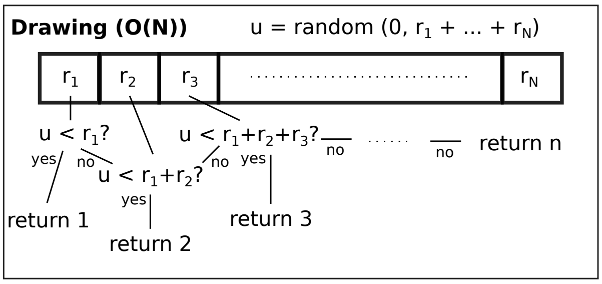

Data: Array r of size N, R = sum (r).
Result: Index drawn according to multinomial drawing.
u = uniform ([0, R));
index = 1;
cum_sum = r[1];
while $u \geq cum\_sum$ **do**
  index = index + 1;
  cum_sum = cum_sum + r [index];
end
return *index*

Figure 36: Direct drawing method

Sketch of algorithm and complexity Worst case of the drawing (Fig. 36) occurs when u is in the last subsegment, so the loop has to be iterated N times, yielding $O(N)$ complexity.

5.1.2 Binary tree

Principle In this approach, we organize propensities inside a tree. Propensities are placed in the leaves of the tree. Nodes are then assembled iteratively 2 by 2 to compute the sum of all propensities (Fig. 37).

The idea is that with the structure *in place*, finding the index that has been drawn

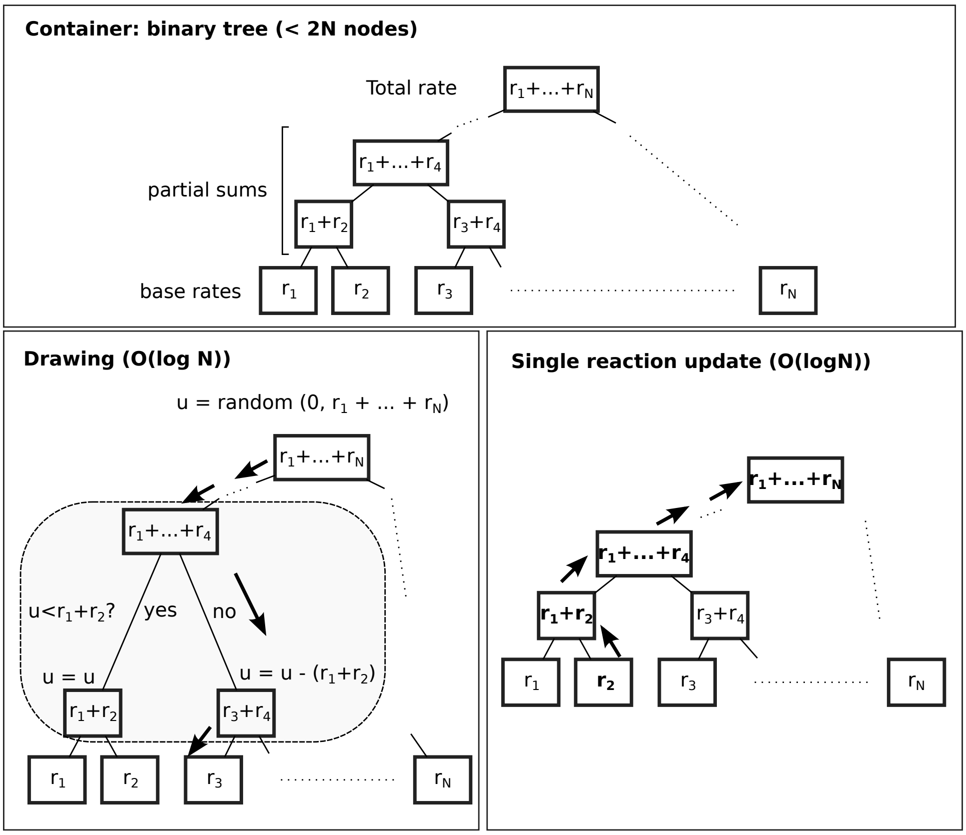

Figure 37: Binary tree containing propensities. Propensity values are found at the leaves of the tree. Intermediate nodes represent partial sums of nodes below, root holds the sum of all propensities.

is quicker. Similarly to the standard drawing, a value u is drawn on the $[0, R = \sum r_i)$ segment. We start from the root node and need to find on which side of the tree u lies. The two children nodes summarize how much weight there is on each side of the tree, say w_{left} and w_{right} respectively. If $u < w_{\text{left}}$, we descend to the left child node and proceed the same way until we reach a leaf. If $u \geq w_{\text{left}}$, we descend to the right child and we proceed iteratively with $u = u - w_{\text{left}}$.

This procedure is actually very similar to the biased wheel method, except we perform some kind of progressive zooming in on the subsegments delimited by the propensity values (Fig. 37).

Sketch of algorithm and complexity Complexity for performing the drawing (Fig. 38) is given by the depth of the tree, $\lceil \log_2 N \rceil$, which is $O(\log N)$.

Complexity for updating the tree (Fig. 39) is also given by depth of the tree, $O(\log N)$. Note that we need not update every node in the tree, only the parents of the updated leaf up to the root node.

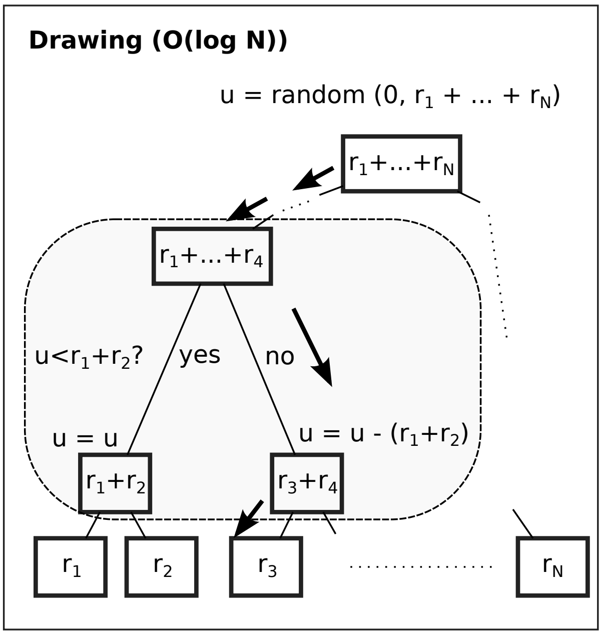

Data: Binary tree with propensities at its leaves.
Result: Index drawn according to multinomial drawing.
u = uniform ([0, tree.root.value));
node = tree.root;
while *node is not a leaf* **do**
  if *u < node.left_child.value* **then**
    node = node.left_child;
  else
    node = node.right_child;
    u = u - node.left_child.value;
  end
end
return *node.index*

Figure 38: Binary tree: drawing method.

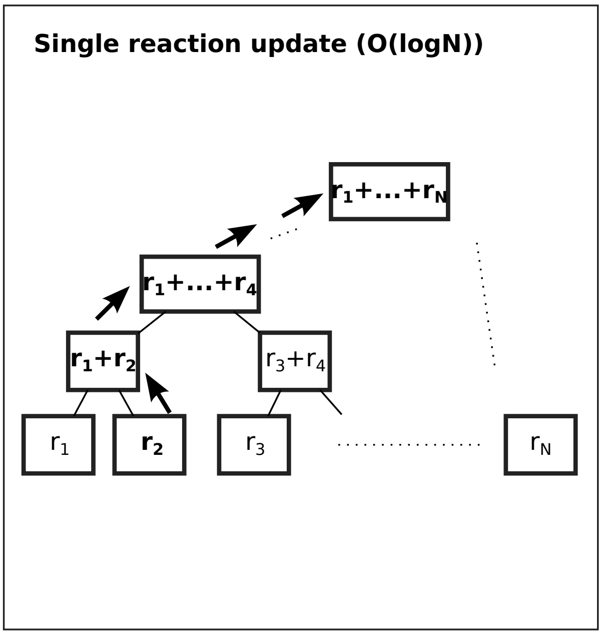

Data: Binary tree with propensities at its leaves. Index i_update of propensity to update, new propensity value p_update.
Result: Updated binary tree.
node = tree.leaf [i_update];
node.value = p_update;
while *node is not root* **do**
  node = node.parent;
  node.value = node.left_child.value + node.right_child.value;
end

Figure 39: Binary tree: update method.

5.1.3 Hybrid method

Principle In this approach, we organize propensities into groups and use a different drawing method: *rejection-base drawing.* This approach has been presented in Slepoy et al. (2008).

Rejection-based drawing The aim is to perform a multinomial drawing, similar to what is done by a biased wheel or a binary tree. The problem with the latter methods is that we need to iterate through some structure before finding the right value. With rejection-based drawing, we attempt to *jump* to the right solution. Let $\{r_i\}$ be the propensities of the N reactions in the system and $R = \sum_{1 \leq i \leq N} r_i$.

1. We choose a value r_M such that $\forall i, r_M \geq r_i$.
2. Until a good candidate is found.
   a) We draw a random number i between 1 and N (with replacement).
   b) We draw a random number u on the $[0, r_M]$ segment. If $u > r_i$, we reject i, else we keep it.

The probability of drawing index i is equal to r_i/R (Serebrinsky, 2011). Note that the choice of r_M is critical for the efficiency of the method (Fig. 40A). Formally, the probability to accept a candidate is $\sum_i \mathbb{P}\,(\text{draw } i)\ \mathbb{P}\,(\text{accept } i) = \sum_i 1/N \times r_i/r_M = R/(Nr_M)$. If applied naively, the number of candidates to loop through is a geometric law with parameter $R/(Nr_M)$. The expected number of candidates is thus Nr_M/R. For a uniform distribution, this value can be 1, but in general, it yields bad results (Fig. 40B).

Group method The idea behind the algorithm is to improve the acceptation probability by placing propensities in *groups*:

1. We draw a group index by using a classical method (biased wheel or binary tree).
2. We draw a propensity inside the group by using the rejection-based method.

Slepoy et al. (2008) propose placing propensities into binary groups. They choose a base rate b. Groups are of the form $(0, b]$, $(b, 2b]$, $(2b, 4b]$, *etc.* $(0, b]$ contains all propensities between 0 and b, and so on. When applying the rejection method to any of these groups (except $(0, b]$), the acceptation probability is $\geq 1/2$ (Fig. 40C). Note that the number of groups K does not generally depend on N, it only depends on the highest propensity value. In general, it remains relatively small.

Suppose the structure is already in place, *i.e.* propensities are placed in the right group and the total propensity for each group is known. Step 1 is at most $O(K)$, which is independent of N. Step 2 requires less than 2 candidates on average, so it is $O(1)$. This results in $O(1)$ globally, making it significantly more efficient than the two previous methods. However, we will see that its implementation is also trickier in order to preserve this theoretical complexity.

Sketch of algorithm and complexity Because of the group structure, the loop in Figure 42 is $O(1)$ (see above). The first multinomial drawing is at most $O(K)$, so the complexity is globally $O(K)$. Because K, the number of groups, does not naturally scale with N, the number of reactions, the complexity is overall $O(1)$, as N is the real variable of interest here.

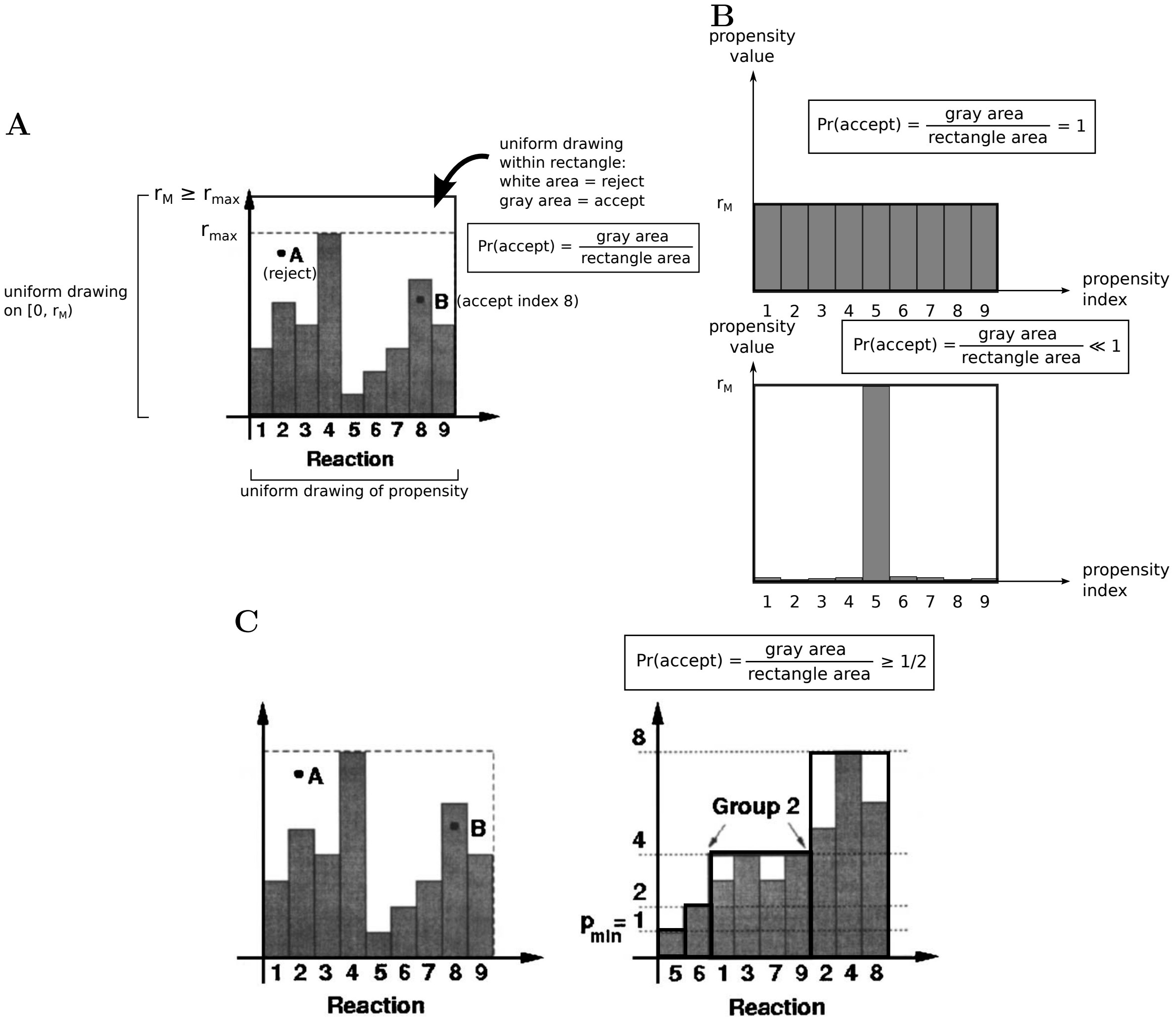

Figure 40: Rejection based drawing (adapted from Slepoy et al. (2008)). (A) Geometric illustration of rejection principle. Drawing occurs in a 2D space, with propensities aligned along the x axis and their value given by gray bars along the y axis. A drawing is accepted if it falls into the gray domain. Note that the probability to draw a propensity is proportional to its value, as an accepted drawing will be distributed uniformly across the gray domain. (B) Examples displaying efficiency of the technique (maximal for uniform propensities, minimal when some are very high and most are very low). (C) Sorting propensities into groups whose limits are powers of 2 ensures a minimal 1/2 acceptation probability *within a given group.*

At first sight, updating the group structure is also $O(1)$ (Fig. 43). However, the parts about removing or inserting a reaction into a group must be carefully implemented in order to achieve that result.

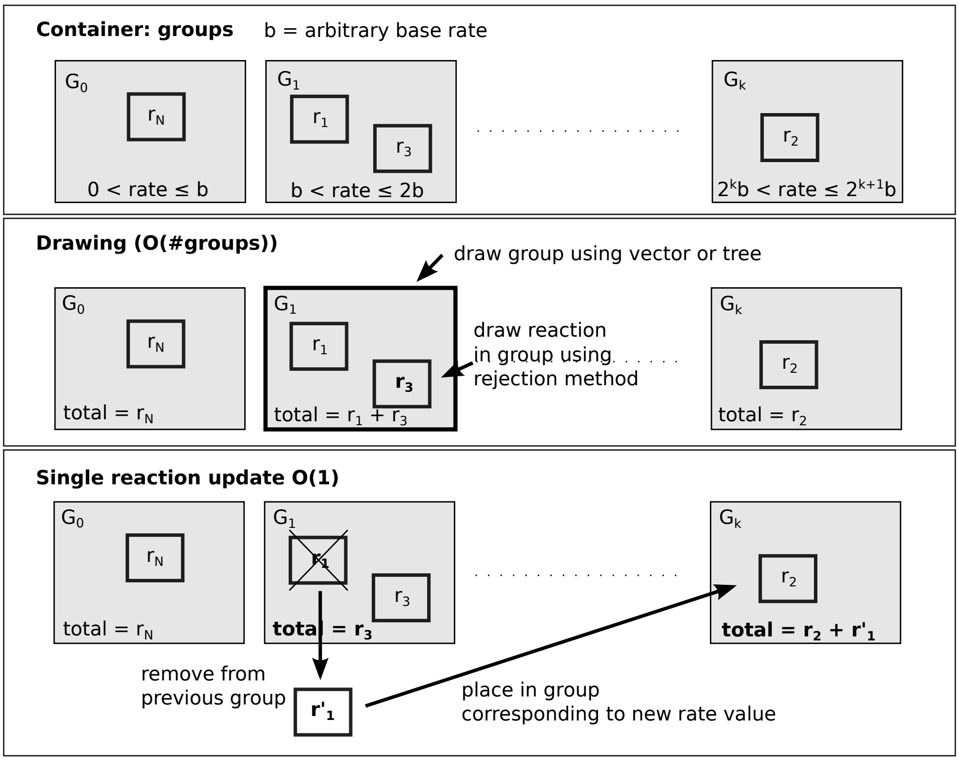

Figure 41: Hybrid method using group structure and rejection algorithm.

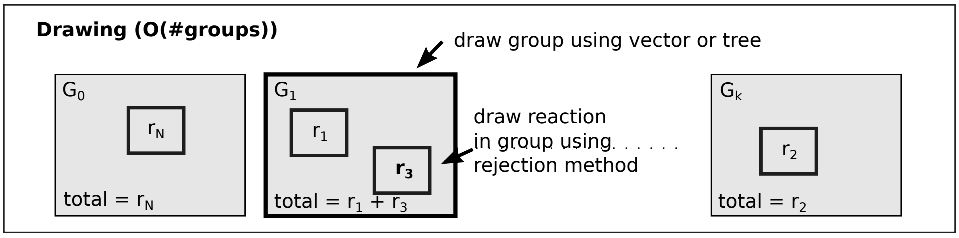

Data: $K+1$ groups, group k containing propensities whose value falls in the interval $(0, b]$ if k=0, $(2^{k-1}b, 2^k b]$ if k > 0. Propensities are stored as a couple containing their value and original index.
Result: Index drawn according to multinomial drawing.
`// drawing using a direct method like binary tree or biased wheel`
group = groups [multinomial (group[0].total_propensity, ..., group[K].total_propensity)];
repeat
  candidate = group.propensities [uniform (1, group.number_propensities)];
until *candidate.value > uniform (0, group.max_propensity)*;
return *candidate.index*

Figure 42: Hybrid method: drawing method.

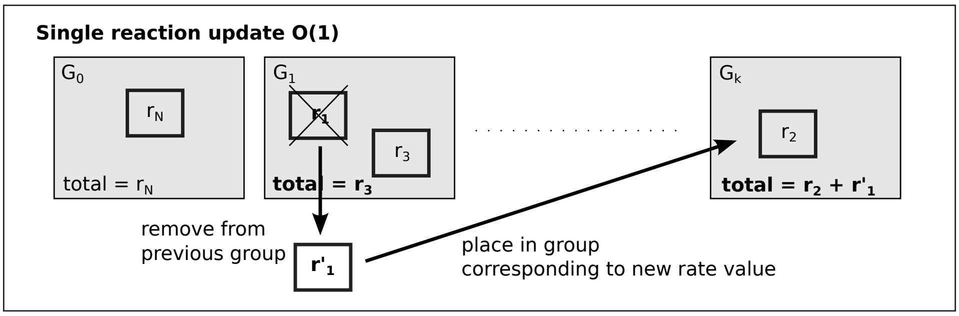

Data: $K+1$ groups, group k containing propensities whose value falls in the interval $(0, b]$ if k=0, $(2^{k-1}b, 2^k b]$ if k > 0. Propensities are stored as a couple containing their value and original index. Index i_update of propensity to update, new propensity value p_update.

Result: Updated group structure.

Function *group_index (propensity)*

  if *propensity* $\geq b$ **then**

    return $\lceil \log_2(\textit{reaction.propensity}/b) \rceil$

  else

    return *0*

  end

propensity = propensity corresponding to index i_update;

previous_group = groups [group_index (propensity.value)];

Remove propensity from previous_group and update group's total propensity;

new_group = groups [group_index (p_update)];

propensity.value = p_update;

Add propensity to new_group and update group's total propensity;

Figure 43: Hybrid method: update method.

5.1.4 Summary

Table 1 summarizes the worst case complexity of the three methods presented. Note that the update complexity was derived in the case where only one propensity needed to be updated. To obtain the overall complexity, we need to take into account the number of reactions U whose propensity needs to be updated. A naive analysis indicates that the binary tree could be less efficient than the direct method depending on U and N (Table 1).

Method	Drawing Complexity	Update Complexity (one propensity)	Total Complexity
Direct Method	$O(N)$	$O(1)$	$O(N)$?
Binary Tree	$O(\log N)$	$O(\log N)$	$O(U \log N)$?
Hybrid Method	$O(1)$	$O(1)$	$O(U)$?

Table 1: Comparison of worst-case complexities of methods implemented in BiPSim. N is the number of reactions in the system, $U \leq N$ the number of reactions whose propensity needs to be updated. The last column is a projection based on the first two columns, real total complexities are given in the next section.

References

Vincent Danos, Jérôme Feret, Walter Fontana, and Jean Krivine. Scalable simulation of cellular signaling networks. In *Lecture Notes in Computer Science (including subseries Lecture Notes in Artificial Intelligence and Lecture Notes in Bioinformatics)*, volume 4807 LNCS, pages 139–157, 2007. ISBN 9783540766360. doi: 10.1007/978-3-540-76637-7_10.

James R Faeder, Michael L Blinov, and William S Hlavacek. Rule-based modeling of biochemical systems with bionetgen. In *Systems biology*, pages 113–167. Springer, 2009.

Daniel T. Gillespie, Andreas Hellander, and Linda R. Petzold. Perspective: Stochastic algorithms for chemical kinetics. *J Chem Phys*, 138 (17), May 2013. ISSN 0021-9606. doi: 10.1063/1.4801941. URL `http://www.ncbi.nlm.nih.gov/pmc/articles/PMC3656953/`.

Santiago A. Serebrinsky. Physical time scale in kinetic Monte Carlo simulations of continuous-time Markov chains. *Phys Rev E*, 83(3), March 2011. ISSN 1539-3755, 1550-2376. doi: 10.1103/PhysRevE.83.037701. URL `http://link.aps.org/doi/10.1103/PhysRevE.83.037701`.

Alexander Slepoy, Aidan P. Thompson, and Steven J. Plimpton. A constant-time kinetic Monte Carlo algorithm for simulation of large biochemical reaction networks. *J Chem Phys*, 128(20):205101, 2008. ISSN 00219606. doi: 10.1063/1.2919546. URL `http://scitation.aip.org/content/aip/journal/jcp/128/20/10.1063/1.2919546`.

Michael W. Sneddon, James R. Faeder, and Thierry Emonet. Efficient modeling, simulation and coarse-graining of biological complexity with NFsim. *Nature Methods*, 8(2):177–183, February 2011. ISSN 1548-7105. doi: 10.1038/nmeth.1546. URL `https://www.nature.com/articles/nmeth.1546`. Number: 2 Publisher: Nature Publishing Group.

BiPSim: a flexible and generic stochastic simulator for polymerization processes - Statistical validation and extensions (Supplementary File 2)

Stephan Fischer[1], Marc Dinh[1], Vincent Henry[1], Philippe Robert[2], Anne Goelzer[1], and Vincent Fromion[1,*]

[1]Université Paris-Saclay, INRAE, MaIAGE, Jouy-en-Josas, France
[2]INRIA Paris, Paris Cedex 12, France
[*]vincent.fromion@inrae.fr

Contents

1 Introduction **3**

2 Statistical validation of BiPSim **3**

2.1 Model `paulsson_minimal` 3

2.2 Validation of gene expression models 4

3 Simulation of complex molecular mechanisms - Multiple DNA forks **6**

1 Introduction

In the main paper, we show the main characteristics of BiPSim, a flexible simulator combining Gillespie simulation with rule-based modeling to represent and efficiently simulate sequence-based reactions stochastically. While the overall trends of RNA and protein production in our simulations are consistent with known data, small mistakes in implementation could lead to subtle biases in BiPSim's output. In the first section, we validate that this is not the case by introduce a BiPSim model that matches Paulsson's theoretical model of RNA and protein production Paulsson (2005), showing that simulated values are consistent with theoretical predictions. In the second section, we show how BiPSim's reactions can be combined to generate more complex models including replication with cascading DNA forks.

2 Statistical validation of BiPSim

2.1 Model `paulsson_minimal`

In the main paper, we showed that the total number of proteins produced in one cell cycle of a gene expression model was consistent with known biology. Here, we are interested in more detailed statistics about the stochastic production of individual genes. To validate the statistics of the simulation algorithm, we implemented Paulsson's model of gene expression Paulsson (2005) (`paulsson_minimal`, Fig. 1a) where promoters are always active. Because `paulsson_minimal` is a strict implementation of Paulsson's model for an active promoter, we expect that the simulated and theoretical distributions of mRNAs and proteins over time match exactly. We start by expliciting the formula of the theoretical distributions, then discuss the agreement between BiPSim's output and theoretical predictions.

Theoretical formulas for the stochastic gene expression model `paulsson_minimal`. We adapted the theoretical formulas given in Paulsson (2005) in the case of an active promoter and using notations of Figure 1. mRNAs are created at a fixed rate λ_r. Existing mRNAs are degraded at rate given by the product between δ_r and its concentration. Proteins are created at rate given by the product between λ_p and the messenger concentration and degraded at rate given by the product between δ_p and the protein concentration. δ_p is assumed to be common to all the proteins since it takes into account the dilution effect due to the cell grows. Following the formula in Paulsson (2005), we have :

- the average of the number of mRNAs (noted n_m): $\langle n_m \rangle = \dfrac{\lambda_r}{\delta_r}$
- the stationary variance in the number of mRNAs: $\sigma_m^2 = \langle n_m \rangle$.
  Since the number of mRNAs follows a Poisson distribution, variance and average of the number of mRNAs are expected to be equal.

- the average of the number of proteins (noted n_p): $\langle n_p \rangle = \dfrac{\lambda_r \lambda_p}{\delta_r \delta_p}$
- the stationary variance in the number of proteins:

$$\sigma_p^2 = \langle n_p \rangle (1 + \frac{\lambda_p}{\delta_r + \delta_p}) = \frac{\lambda_r \lambda_p}{\delta_r \delta_p}(1 + \frac{\lambda_p}{\delta_r + \delta_p})$$

The set of model parameters associated to the i-th gene is denoted by $(\lambda_{ri}, \delta_{ri}, \lambda_{pi}, \delta_d)$.

Parameter estimation from omics data. Parameters (λ_{ri}, λ_{pi}, δ_{ri}, δ_p) of each gene were identified using available datasets of absolute protein abundances Goelzer et al. (2015), absolute messenger abundances Borkowski et al. (2016), known half-lifes of messengers Hambraeus et al. (2003). For the i-th gene, model parameters (λ_{ir}, λ_{ip}, δ_{ir}, δ_p) are related to the messenger abundance $\bar{m}_i$ and the protein abundance $\bar{P}_i$ by the following expressions Paulsson (2005):

$$\bar{m}_i = \frac{\lambda_{ir}}{\delta_{ir}} \quad \text{and} \quad \bar{P}_i = \frac{\lambda_{ip}\bar{m}_i}{\delta_p}. \tag{2.1}$$

Parameter δ_p corresponds to protein dilution, and set to $1/\tau$ with $\tau = 40$min the generation time. We computed $\lambda_{ip} = \delta_p \bar{P}_i / \bar{m}_i$ for genes where both abundances of $\bar{m}_i$ and $\bar{P}_i$ were available. When either $\bar{m}_i$ or $\bar{P}_i$ was missing within the dataset, the corresponding λ_{ip} was sampled from the empirical distribution of known λ_{ip}.Parameters δ_{ir} correspond to the inverse of the half-life of messengers and were sampled from a lognormal distribution of known half-life of messengers for *B. subtilis* given in Hambraeus et al. (2003), having a mean of 0.6 and a standard deviation of 0.8. Then we computed $\lambda_{ir} = \bar{m}_i \delta_{ir}$ for genes where messenger abundances were available in Borkowski et al. (2016). We sampled remaining λ_{ir} from the distribution of known λ_{ir}.We checked *a posteriori* the empirical cumulative distributions of mRNA and protein numbers per cell.

2.2 Validation of gene expression models

For the BiPSim model `paulsson_minimal`, we used the same sequence information as models `ge_detailed`, `ge_aggregated` and `ge_hybrid` in the main document, except for the organization of genes within transcription units. To match Paulsson's model, we replaced annotated TUs (which typically include polycistronic RNAs) by artifical TUs, where every TU contains exactly one gene. While not realistic, this modification is essential for comparison of simulations with theoretical values. In order to generate the diversity of protein production rates observed in real cells, we estimated reaction rates using available datasets of absolute protein abundances Goelzer et al. (2015), absolute messenger abundances Borkowski et al. (2016), and known half-lifes of messengers Hambraeus et al. (2003) as previously described. To avoid burn-in, we initiated mRNA and protein abundances to the expected theoretical values and ran the simulation of the model for 125 cycles.

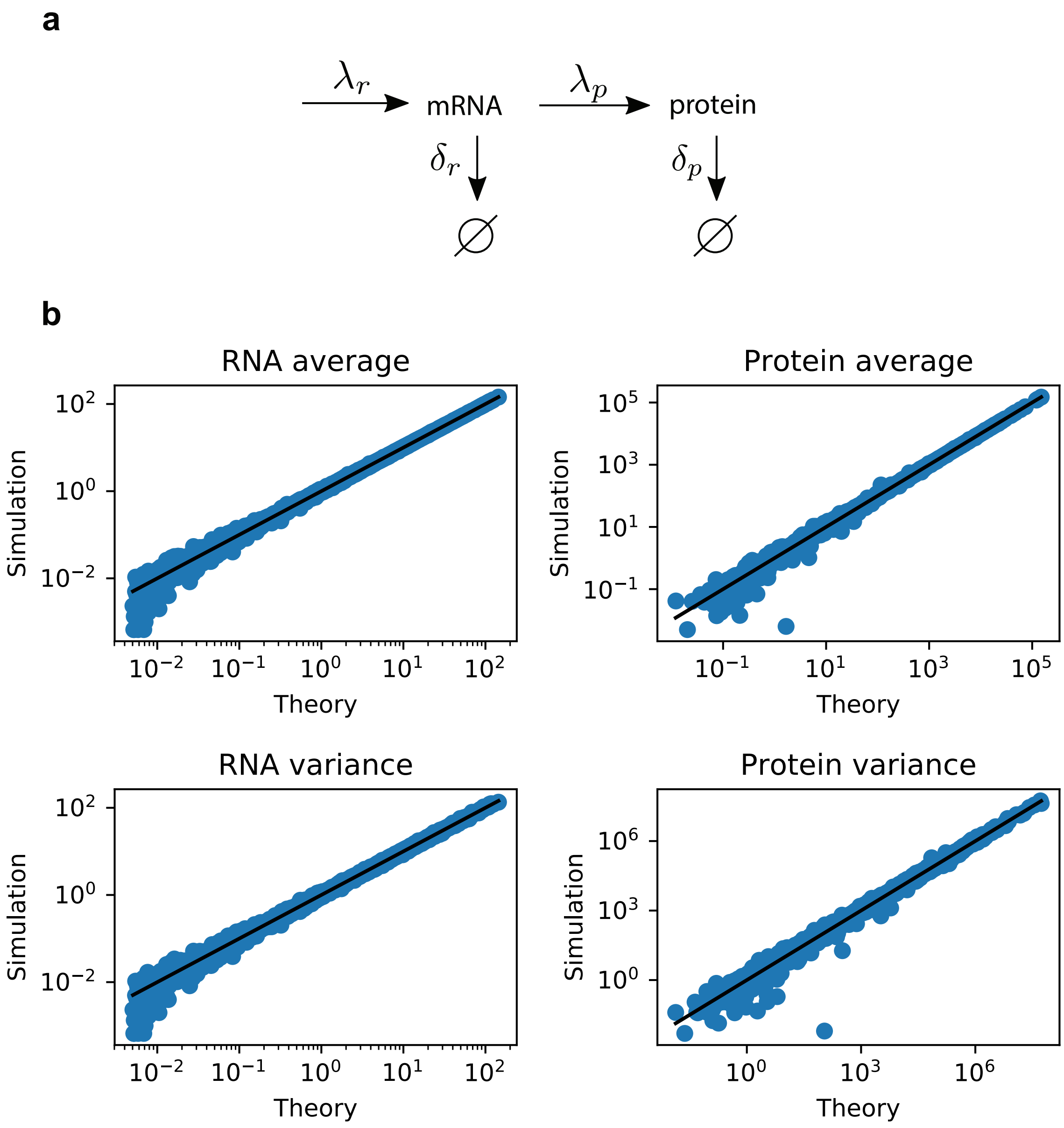

Figure 1: **a**, Standard model of gene expression `paulsson_minimal`. For every gene, four parameters are defined: mRNA production rate λ_r, mRNA degradation rate δ_r, protein production rate λ_p and protein degradation rate δ_p. **b**, Simulated statistics of the model `paulsson_minimal` plotted against theoretical values. Average numbers are number of molecules, variance is in number of molecules squared. Statistics of simulation match very well with theoretical predictions. Convergence of estimators is slow for some genes (with high λ_p.) Residuals are well-centered (log-scale is misleading here) and their scattering is consistent with estimators used.

Average values and variances match with theoretical predictions (Fig. 1). We did not observe any systematic over- or undervaluation (residuals centered around 0). For mRNAs, estimators are within 10% of theoretical values for populations that are expected to have more than 4 copies. For proteins, estimators are within 10% of theoretical values for populations that are expected to have more than 1000 copies. By running simulations longer, estimators converge further towards theoretical values without apparent biases, which validates the implementation of the simulation algorithm.

3 Simulation of complex molecular mechanisms - Multiple DNA forks

In this section, we display a simple model illustrating the multiple DNA fork feature of BiPSim. The model uses *exactly* the same set of reactions as the simulations shown in the main paper (listed in the BiPSim repository under `input/replication.in`). In all models, we assumed that a single molecule of DnaA binds to the origin of replication and recruits a DNA polymerase. Simulations start with two molecules of DnaA-ATP, triggering intiation of replication around the beginnig of the simulation. Binding of DnaA is fast compared to DNA polymerase recruitment, ensuring that both sense and antisense DNA at origin are bound by DnaA and start independent replicating forks. Furthemore, we suppose that DnaA is hydrolyzed, avoiding re-initiation.

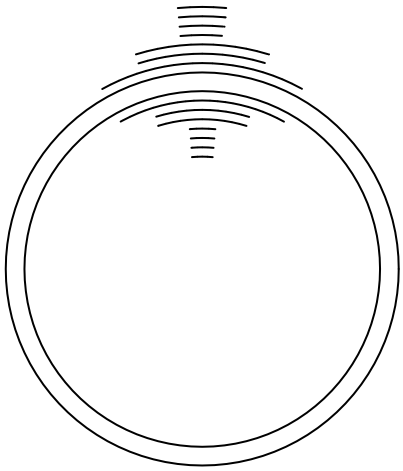

Figure 2: DNA strands at $t = 500s$. Rounds of replication were started at $t = 0s$, $t = 200s$ and $t = 400s$. Note that Okazaki fragments are not visible at this scale. Here we built a simple model to display the multiple DNA fork feature of BiPSim. In this model, we assume that a single molecule of DnaA-ATP binds to the origin of replication, then recruits a DNA polymerase and is further hydrolyzed to prevent reinitiation.

For the present model, we removed all reactions that are not related to replication (for illustration purposes) and added injections of DnaA-ATP to force replication re-initiation. At $t = 200s$, we add 4 molecules of DnaA-ATP. These molecules start 4 new replicating forks at the 2 origins. At $t = 400s$, we add 8 molecules of DnaA-ATP. These molecules start 8 new replicating forks at the 4 origins. At $t = 500s$, we have 14 independent replication forks (Fig. 2).

This simulation displays how an existing model can be modified to contain new features. Because BiPSim's formalism provides low-level descriptions, simple changes in input files can result in very different models.

References

O. Borkowski, A. Goelzer, M. Schaffer, M. Calabre, U. Mäder, S. Aymerich, M. Jules, and V. Fromion. Translation elicits a growth rate-dependent, genome-wide, differential protein production in *Bacillus subtilis*. *Mol. Sys. Biol.*, 12(5):870, 2016.

A. Goelzer, J. Muntel, V. Chubukov, M. Jules, E. Prestel, R. Nölker, M. Mariadassou, S. Aymerich, M. Hecker, P. Noirot, D. Becher, and V. Fromion. Quantitative prediction of genome-wide resource allocation in bacteria. *Metab. Eng.*, 32:232–243, 2015.

G. Hambraeus, C. von Wachenfeldt, and L. Hederstedt. Genome-wide survey of mrna half-lives in bacillus subtilis identifies extremely stable mrnas. *Molecular Genetics and Genomics*, 269(5):706–714, 2003.

J. Paulsson. Models of stochastic gene expression. *Phys Life Rev*, 2(2):157–175, June 2005. ISSN 15710645. doi: 10.1016/j.plrev.2005.03.003. URL `http://linkinghub.elsevier.com/retrieve/pii/S1571064505000138`.
