# Supplementary material for: BiPSim: a flexible and generic stochastic simulator for polymerization processes
Source: Sci Rep. 2021 Jul 8;11:14112. doi: 10.1038/s41598-021-92833-5 (PMC8266833; doi:10.1038/s41598-021-92833-5)
Supplement: Supplementary file 3 — Supplementary Information 3. [file 41598_2021_92833_MOESM3_ESM.pdf]

BiPSim: a flexible and generic stochastic simulator for polymerization processes - Statistical validation and extensions (Supplementary File 2)

Stephan Fischer[1], Marc Dinh[1], Vincent Henry[1], Philippe Robert[2], Anne Goelzer[1], and Vincent Fromion[1,*]

[1]Université Paris-Saclay, INRAE, MaIAGE, Jouy-en-Josas, France
[2]INRIA Paris, Paris Cedex 12, France
[*]vincent.fromion@inrae.fr

Contents

1 Introduction **3**

2 Statistical validation of BiPSim **3**

2.1 Model `paulsson_minimal` 3

2.2 Validation of gene expression models 4

3 Simulation of complex molecular mechanisms - Multiple DNA forks **6**

1 Introduction

In the main paper, we show the main characteristics of BiPSim, a flexible simulator combining Gillespie simulation with rule-based modeling to represent and efficiently simulate sequence-based reactions stochastically. While the overall trends of RNA and protein production in our simulations are consistent with known data, small mistakes in implementation could lead to subtle biases in BiPSim's output. In the first section, we validate that this is not the case by introduce a BiPSim model that matches Paulsson's theoretical model of RNA and protein production Paulsson (2005), showing that simulated values are consistent with theoretical predictions. In the second section, we show how BiPSim's reactions can be combined to generate more complex models including replication with cascading DNA forks.

2 Statistical validation of BiPSim

2.1 Model `paulsson_minimal`

In the main paper, we showed that the total number of proteins produced in one cell cycle of a gene expression model was consistent with known biology. Here, we are interested in more detailed statistics about the stochastic production of individual genes. To validate the statistics of the simulation algorithm, we implemented Paulsson's model of gene expression Paulsson (2005) (`paulsson_minimal`, Fig. 1a) where promoters are always active. Because `paulsson_minimal` is a strict implementation of Paulsson's model for an active promoter, we expect that the simulated and theoretical distributions of mRNAs and proteins over time match exactly. We start by expliciting the formula of the theoretical distributions, then discuss the agreement between BiPSim's output and theoretical predictions.

Theoretical formulas for the stochastic gene expression model `paulsson_minimal`. We adapted the theoretical formulas given in Paulsson (2005) in the case of an active promoter and using notations of Figure 1. mRNAs are created at a fixed rate λ_r. Existing mRNAs are degraded at rate given by the product between δ_r and its concentration. Proteins are created at rate given by the product between λ_p and the messenger concentration and degraded at rate given by the product between δ_p and the protein concentration. δ_p is assumed to be common to all the proteins since it takes into account the dilution effect due to the cell grows. Following the formula in Paulsson (2005), we have :

- the average of the number of mRNAs (noted n_m): $\langle n_m \rangle = \dfrac{\lambda_r}{\delta_r}$
- the stationary variance in the number of mRNAs: $\sigma_m^2 = \langle n_m \rangle$.
  Since the number of mRNAs follows a Poisson distribution, variance and average of the number of mRNAs are expected to be equal.

- the average of the number of proteins (noted n_p): $\langle n_p \rangle = \frac{\lambda_r \lambda_p}{\delta_r \delta_p}$
- the stationary variance in the number of proteins:

$$\sigma_p^2 = \langle n_p \rangle (1 + \frac{\lambda_p}{\delta_r + \delta_p}) = \frac{\lambda_r \lambda_p}{\delta_r \delta_p} (1 + \frac{\lambda_p}{\delta_r + \delta_p})$$

The set of model parameters associated to the i-th gene is denoted by $(\lambda_{ri}, \delta_{ri}, \lambda_{pi}, \delta_d)$.

Parameter estimation from omics data. Parameters (λ_{ri}, λ_{pi}, δ_{ri}, δ_p) of each gene were identified using available datasets of absolute protein abundances Goelzer et al. (2015), absolute messenger abundances Borkowski et al. (2016), known half-lifes of messengers Hambraeus et al. (2003). For the i-th gene, model parameters (λ_{ir}, λ_{ip}, δ_{ir}, δ_p) are related to the messenger abundance $\bar{m}_i$ and the protein abundance $\bar{P}_i$ by the following expressions Paulsson (2005):

$$\bar{m}_i = \frac{\lambda_{ir}}{\delta_{ir}} \quad \text{and} \quad \bar{P}_i = \frac{\lambda_{ip} \bar{m}_i}{\delta_p}. \tag{2.1}$$

Parameter δ_p corresponds to protein dilution, and set to $1/\tau$ with $\tau = 40$min the generation time. We computed $\lambda_{ip} = \delta_p \bar{P}_i / \bar{m}_i$ for genes where both abundances of $\bar{m}_i$ and $\bar{P}_i$ were available. When either $\bar{m}_i$ or $\bar{P}_i$ was missing within the dataset, the corresponding λ_{ip} was sampled from the empirical distribution of known λ_{ip}.Parameters δ_{ir} correspond to the inverse of the half-life of messengers and were sampled from a lognormal distribution of known half-life of messengers for *B. subtilis* given in Hambraeus et al. (2003), having a mean of 0.6 and a standard deviation of 0.8. Then we computed $\lambda_{ir} = \bar{m}_i \delta_{ir}$ for genes where messenger abundances were available in Borkowski et al. (2016). We sampled remaining λ_{ir} from the distribution of known λ_{ir}.We checked *a posteriori* the empirical cumulative distributions of mRNA and protein numbers per cell.

2.2 Validation of gene expression models

For the BiPSim model `paulsson_minimal`, we used the same sequence information as models `ge_detailed`, `ge_aggregated` and `ge_hybrid` in the main document, except for the organization of genes within transcription units. To match Paulsson's model, we replaced annotated TUs (which typically include polycistronic RNAs) by artifical TUs, where every TU contains exactly one gene. While not realistic, this modification is essential for comparison of simulations with theoretical values. In order to generate the diversity of protein production rates observed in real cells, we estimated reaction rates using available datasets of absolute protein abundances Goelzer et al. (2015), absolute messenger abundances Borkowski et al. (2016), and known half-lifes of messengers Hambraeus et al. (2003) as previously described. To avoid burn-in, we initiated mRNA and protein abundances to the expected theoretical values and ran the simulation of the model for 125 cycles.

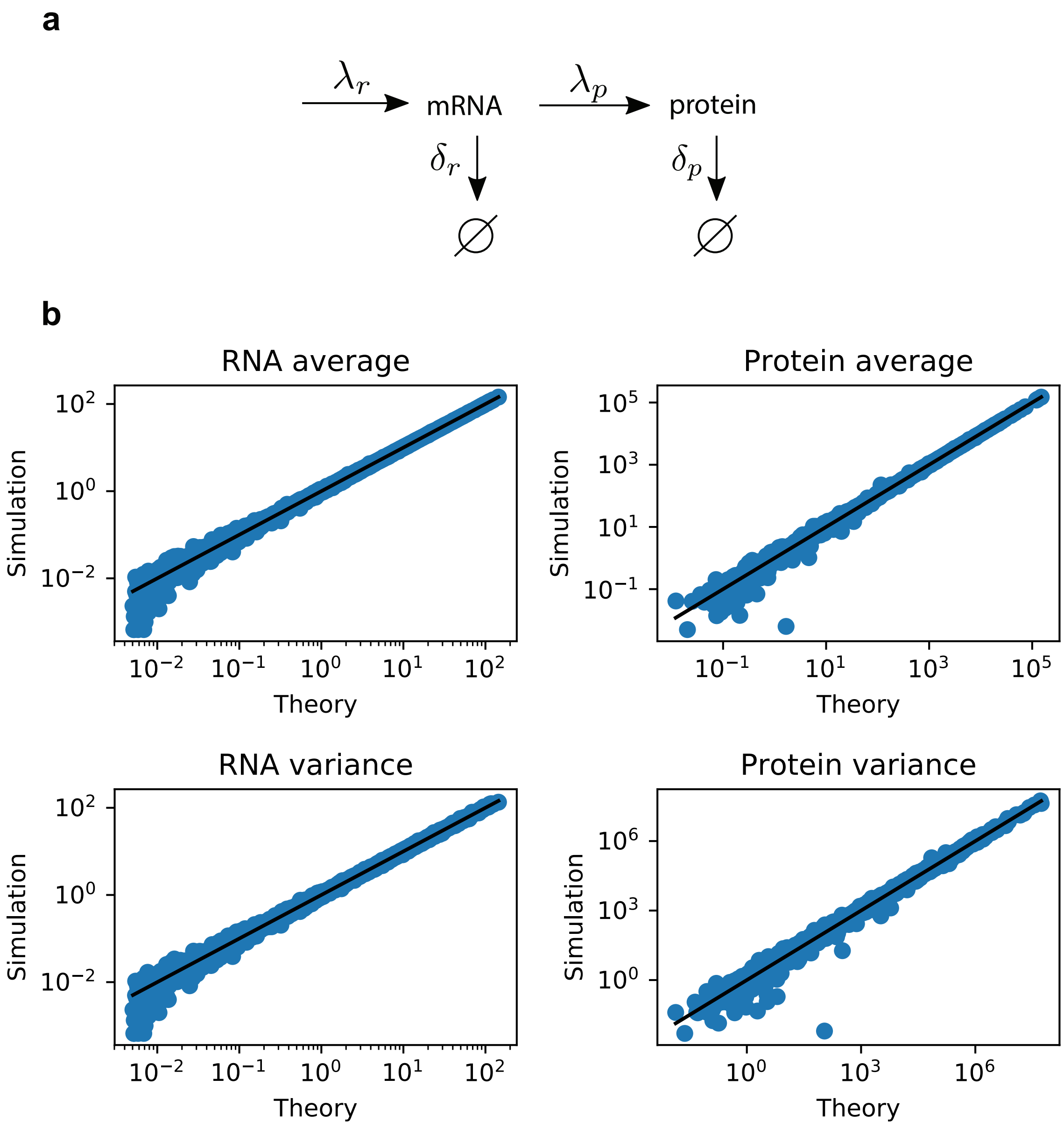

Figure 1: **a**, Standard model of gene expression `paulsson_minimal`. For every gene, four parameters are defined: mRNA production rate λ_r, mRNA degradation rate δ_r, protein production rate λ_p and protein degradation rate δ_p. **b**, Simulated statistics of the model `paulsson_minimal` plotted against theoretical values. Average numbers are number of molecules, variance is in number of molecules squared. Statistics of simulation match very well with theoretical predictions. Convergence of estimators is slow for some genes (with high λ_p.) Residuals are well-centered (log-scale is misleading here) and their scattering is consistent with estimators used.

Average values and variances match with theoretical predictions (Fig. 1). We did not observe any systematic over- or undervaluation (residuals centered around 0). For mRNAs, estimators are within 10% of theoretical values for populations that are expected to have more than 4 copies. For proteins, estimators are within 10% of theoretical values for populations that are expected to have more than 1000 copies. By running simulations longer, estimators converge further towards theoretical values without apparent biases, which validates the implementation of the simulation algorithm.

3 Simulation of complex molecular mechanisms - Multiple DNA forks

In this section, we display a simple model illustrating the multiple DNA fork feature of BiPSim. The model uses *exactly* the same set of reactions as the simulations shown in the main paper (listed in the BiPSim repository under `input/replication.in`). In all models, we assumed that a single molecule of DnaA binds to the origin of replication and recruits a DNA polymerase. Simulations start with two molecules of DnaA-ATP, triggering intiation of replication around the beginnig of the simulation. Binding of DnaA is fast compared to DNA polymerase recruitment, ensuring that both sense and antisense DNA at origin are bound by DnaA and start independent replicating forks. Furthemore, we suppose that DnaA is hydrolyzed, avoiding re-initiation.

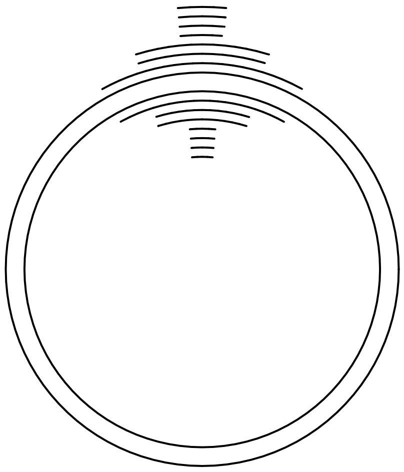

Figure 2: DNA strands at $t = 500s$. Rounds of replication were started at $t = 0s$, $t = 200s$ and $t = 400s$. Note that Okazaki fragments are not visible at this scale. Here we built a simple model to display the multiple DNA fork feature of BiPSim. In this model, we assume that a single molecule of DnaA-ATP binds to the origin of replication, then recruits a DNA polymerase and is further hydrolyzed to prevent reinitiation.

For the present model, we removed all reactions that are not related to replication (for illustration purposes) and added injections of DnaA-ATP to force replication re-initiation. At $t = 200s$, we add 4 molecules of DnaA-ATP. These molecules start 4 new replicating forks at the 2 origins. At $t = 400s$, we add 8 molecules of DnaA-ATP. These molecules start 8 new replicating forks at the 4 origins. At $t = 500s$, we have 14 independent replication forks (Fig. 2).

This simulation displays how an existing model can be modified to contain new features. Because BiPSim's formalism provides low-level descriptions, simple changes in input files can result in very different models.

References

O. Borkowski, A. Goelzer, M. Schaffer, M. Calabre, U. Mäder, S. Aymerich, M. Jules, and V. Fromion. Translation elicits a growth rate-dependent, genome-wide, differential protein production in *Bacillus subtilis*. *Mol. Sys. Biol.*, 12(5):870, 2016.

A. Goelzer, J. Muntel, V. Chubukov, M. Jules, E. Prestel, R. Nölker, M. Mariadassou, S. Aymerich, M. Hecker, P. Noirot, D. Becher, and V. Fromion. Quantitative prediction of genome-wide resource allocation in bacteria. *Metab. Eng.*, 32:232–243, 2015.

G. Hambraeus, C. von Wachenfeldt, and L. Hederstedt. Genome-wide survey of mrna half-lives in bacillus subtilis identifies extremely stable mrnas. *Molecular Genetics and Genomics*, 269(5):706–714, 2003.

J. Paulsson. Models of stochastic gene expression. *Phys Life Rev*, 2(2):157–175, June 2005. ISSN 15710645. doi: 10.1016/j.plrev.2005.03.003. URL `http://linkinghub.elsevier.com/retrieve/pii/S1571064505000138`.
